# Angiotensin II type-1 receptor-associated protein interacts with transferrin receptor-1 and promotes its internalization

**DOI:** 10.1038/s41598-022-22343-5

**Published:** 2022-10-17

**Authors:** Eriko Abe, Akio Yamashita, Keigo Hirota, Takahiro Yamaji, Kengo Azushima, Shingo Urate, Toru Suzuki, Shohei Tanaka, Shinya Taguchi, Shunichiro Tsukamoto, Tatsuki Uehara, Hiromichi Wakui, Kouichi Tamura, Hidehisa Takahashi

**Affiliations:** 1 Department of Medical Science and Cardiorenal Medicine, Yokohama City University Graduate School of Medicine, Yokohama, Japan; 2 Department of Molecular Biology, Yokohama City University Graduate School of Medicine, Yokohama, Japan; 3 Department of Investigative Medicine Graduate School of Medicine, University of the Ryukyus, Okinawa, Japan; 4 Cardiovascular and Metabolic Disorders Program, Duke-NUS Medical School, Singapore, Singapore

**Keywords:** Cell biology, Molecular biology, Nephrology

## Abstract

Kidney fibrosis is a common pathway that leads to chronic kidney disease. Angiotensin II type-1 receptor (AT1R)-associated protein (ATRAP) was originally identified as an AT1R-binding protein. Previously, we reported that systemic knockout of ATRAP exacerbates kidney fibrosis in aged mice. Although these effects of ATRAP appeared to be AT1R-independent actions, the molecular mechanism remains poorly understood. To elucidate the molecular mechanism of ATRAP independent of AT1R, we explored novel ATRAP-interacting proteins. Mass spectrometric analysis of the immunoprecipitants of a Flag-tagged ATRAP complex revealed 376 candidate proteins that potentially interact with ATRAP. Gene ontology analysis revealed that proteins related to vesicle trafficking, membrane transport, and many membrane proteins, including transferrin receptor 1 (TfR1), were enriched. Because TfR1 promotes cellular iron uptake and iron is a key factor involved in kidney fibrosis, we focused on TfR1 and confirmed that it interacts with ATRAP. In addition, our findings revealed that enhanced ATRAP expression decreased cell-surface TfR1 expression without altering the overall cellular TfR1 expression levels. Furthermore, enhanced ATRAP expression attenuated cellular iron levels. Together, our results highlight the role of ATRAP as a suppressor of TfR1 that functions by facilitating TfR1 internalization, which affects iron metabolism and oxidative stress signaling.

## Introduction

Chronic kidney disease (CKD) is a syndrome defined by persistent alterations in the structure and/or functions of the kidneys with clinical implications. CKD may affect approximately 10% of the worldwide population, and this percentage increases with aging^1^. In CKD, fibrosis is one of the main factors that affects the kidneys. The degree of fibrosis has been correlated with a corresponding subsequent decline in renal function^2^.

The angiotensin II type 1 receptor (AT1R) plays a critical role by signaling through the activated tissue renin–angiotensin system^3^. AT1R-associated protein (ATRAP) was originally identified as a specific binding protein of AT1R. ATRAP promotes angiotensin II-induced AT1R internalization, which inhibits the pathological consequences of its downstream signaling^4^.

Despite the lack of angiotensin II stimulation, we observed that the systemic knockout (KO) of ATRAP exacerbated aging-associated kidney fibrosis and led to increased reactive oxygen species (ROS) production and mitochondrial abnormalities in mice^5^. Although the mechanism whereby ATRAP deficiency induced a novel molecular mechanism of ATRAP involving AT1R-independent signaling was not determined, we hypothesized that uncharacterized ATRAP-associated proteins, other than AT1R, are involved in this phenotype. Therefore, it is critical to know the ATRAP associated factors involved in ROS repression.

ROS are critical factors related to the pathological mechanism of kidney fibrosis. ROS are unstable and highly reactive, making them capable of oxidizing DNA, proteins, and lipids, in turn leading to cellular injuries that induce the activation of pro-fibrotic factors^6–8^.

Iron can cause the production of ROS^9^. However, iron is also an important element for living organisms that participates in oxygen transport, enzymatic activities, and energy production^10^. Therefore, an understanding of the precise regulatory mechanisms of iron is needed to maintain proper iron metabolism.

TfR1 is a ubiquitously expressed plasma membrane receptor that endocytoses iron-bound transferrin (Tf), hereafter holo-Tf, and incorporates iron into cells, thereby help maintain cellular iron levels and iron-associated ROS production^11^.

In the kidneys, TfR1 plays critical roles in iron metabolism in proximal tubular cells^12^. In addition, the kidneys require proper iron levels to produce sufficient energy. Consistent with critical role of TfR1 in the kidneys, heterozygous *TfR1* KO reduced ROS levels and attenuated kidney fibrosis in a mouse model of unilateral ureteral obstruction (UUO) (used to model kidney fibrosis)^13^. However, the detailed regulatory mechanism of TfR1 remains unclear.

In this study, we explored a novel ATRAP-interacting protein in human embryonic kidney 293 (HEK293) cells to elucidate a molecular mechanism of ATRAP involving AT1R-independent signaling. We identified interesting candidate ATRAP-interacting proteins such as vesicle trafficking-related proteins and many membrane proteins including TfR1. As TfR1 is involved in iron-associated ROS production, we focused on TfR1 to resolve the function of ATRAP in the repression of cellular ROS levels. For this purpose, we examined molecular interactions between ATRAP and TfR1. In addition, we investigated the effects of the enhanced ATRAP expression on TfR1 expression levels and localization, as well as the cellular iron levels and oxidative stress signaling.

## Results

### Establishment of HEK293 cells capable of doxycycline (Dox)-induced Flag-ATRAP expression

**Figure 1:**
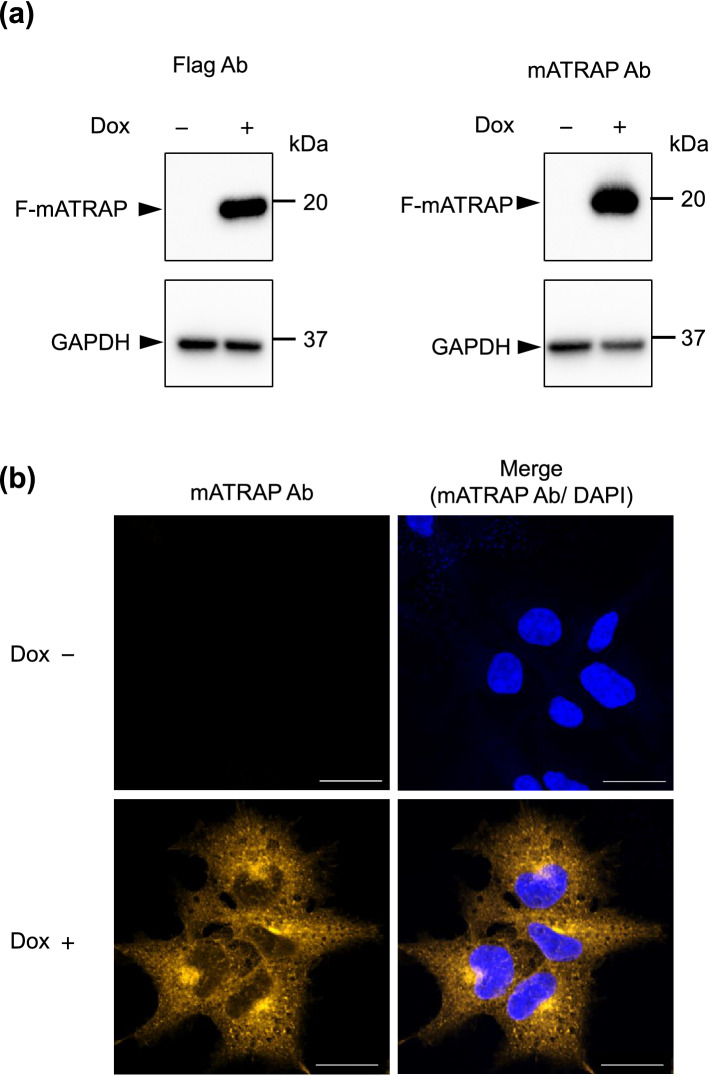
Establishment of HEK293 cells capable of Dox-dependent induction of F-mATRAP expression (HEK293_F-mATRAP cells). (**a**) Western blot analysis using an anti-Flag antibody (left) and an anti-mATRAP antibody (right), with or without Dox treatment (Dox+/Dox−). Glyceraldehyde 3-phosphate dehydrogenase (GAPDH) was probed as a loading control. (**b**) Immunofluorescence analysis using an anti-mATRAP antibody with or without Dox treatment (Dox+/Dox−). Scale bars: 20 µm. Blue: DAPI; Orange: anti-mATRAP antibody. *Ab*: antibody.

To identify ATRAP-interacting proteins, we established an HEK293 cell line that stably expressed a Flag-tagged variant of mouse ATRAP (F-mATRAP) in a Dox-dependent manner. As shown in Fig. 1a, Dox treatment induced F-mATRAP expression, which was detected using an anti-Flag antibody or a newly developed anti-mATRAP antibody. The mATRAP antibody specificity was confirmed by probing human (HEK293) and mouse (mDCT; murine distal convoluted tubule) cell lines, and kidney lysate from *ATRAP*-KO mice. The anti-mATRAP antibody detects endogenous mouse ATRAP but does not detect human ATRAP (Supplementary Fig. S1c,d). Because ATRAP is localized to endosomes^14,15^ and excess ATRAP expression generates aggregated cytoplasmic structures, we analyzed the cellular localization of exogenous F-mATRAP using our new antibody. The immunofluorescent staining results showed that the exogenous F-mATRAP protein localized to the cytoplasm (similar to a previous report showing cellular localization of endogenous mouse ATRAP in primary vascular smooth muscle cells^14^), and the F-mATRAP expression was Dox-dependent (Fig. 1b). The absence of aggregated F-mATRAP complexes suggested that proper localization of exogenous F-mATRAP occurred under our experimental conditions.

### Identification of ATRAP-binding proteins using mass spectrometry (MS)

**Figure 2:**
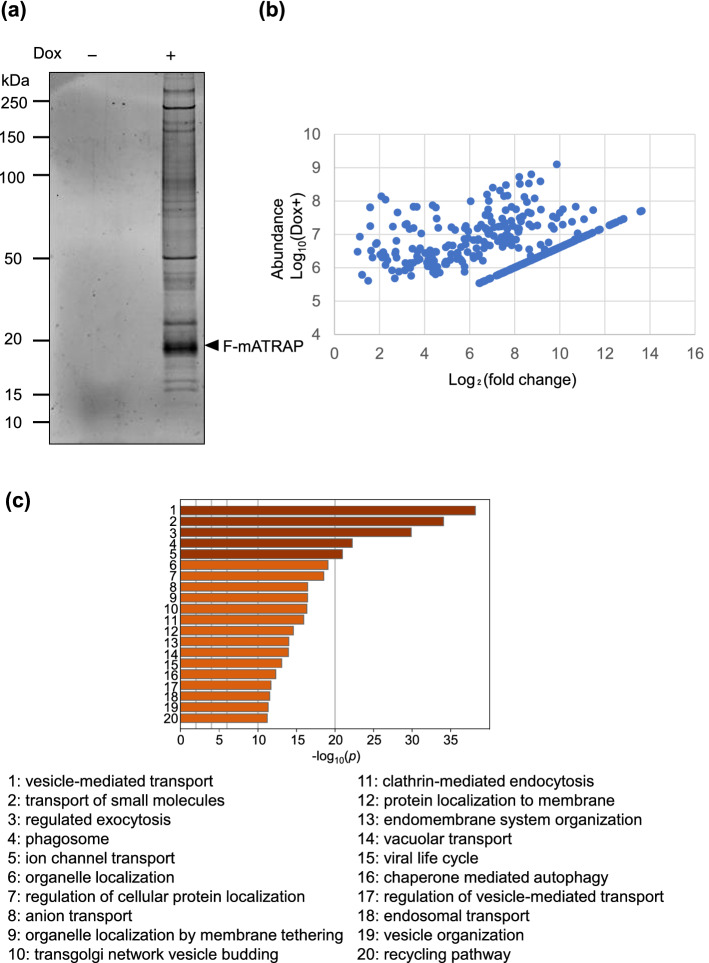
Identification of novel ATRAP-interacting proteins. (**a**) Oriole staining of affinity-purified F-mATRAP complexes. Dox-inducible HEK293_F-mATRAP cells were treated with or without Dox (Dox+/−). The cell lysates were affinity-purified with anti-DYKKKK magnetic beads, fractions eluted using a Flag peptide were separated by SDS-PAGE, and the proteins were visualized with Oriole Fluorescent Gel Stain. (**b**) Scatter plots were drawn to depict the abundance of candidate proteins. The horizontal line shows the log_2_-based fold-changes in abundance ratios of ATRAP-interacting proteins in Dox+ versus Dox− cells. The vertical line shows the corresponding abundances in Dox+ cells (log_10_). (**c**) GO analysis of 376 candidate proteins. Significantly enriched GO terms are shown along with their *p* values. The top 20 most enriched GO terms were visualized. “Log_10_ (*p*)” indicates the log_10_ of the *p*-value.

Next, we affinity-purified F-mATRAP complexes from the lysate of an established cell line using an anti-DYKKKK (an alternative name of the Flag epitope) antibody-conjugated magnetic beads. Many Oriole-stained proteins co-purified with F-mATRAP (Fig. 2a). To identify these proteins, we performed a shotgun proteomics experiment based on separation via gel electrophoresis and liquid–chromatography with tandem MS (LC–MS/MS). We identified 822 proteins as candidate F-mATRAP-associated proteins. After evaluating several cutoffs described in the methods section, we selected 376 proteins for further analysis (Fig. 2b). In addition to AT1R, SERCA2a^16,17^, CAML^18^, Rdgbβ^19,20^, and RACK1^21^ can interact with ATRAP. In this study, only SERCA2a was identified as an ATRAP-interacting protein using MS. This finding may reflect the expression levels and/or solubility of these proteins under our experimental conditions.

To analyze the cellular functions of F-mATRAP-associated proteins, we performed Gene Ontology (GO) analysis. The GO analysis indicated that proteins related to endocytosis and vesicle trafficking were enriched in F-mATRAP complexes, such as vesicle-mediated transport (rank 1), regulated exocytosis (rank 3), and clathrin-mediated endocytosis (rank 11) (Fig. 2c). In addition to proteins related to cellular trafficking, many membrane proteins, including TfR1, were identified (Supplementary Table S1). The abundance of TfR1 was the sixth highest among all candidate proteins. TfR1 is involved in cellular ROS production, mitochondrial functions, and renal fibrosis^13^; thus, we focused on TfR1 in further investigations.

### ATRAP interacted with TfR1

**Figure 3:**
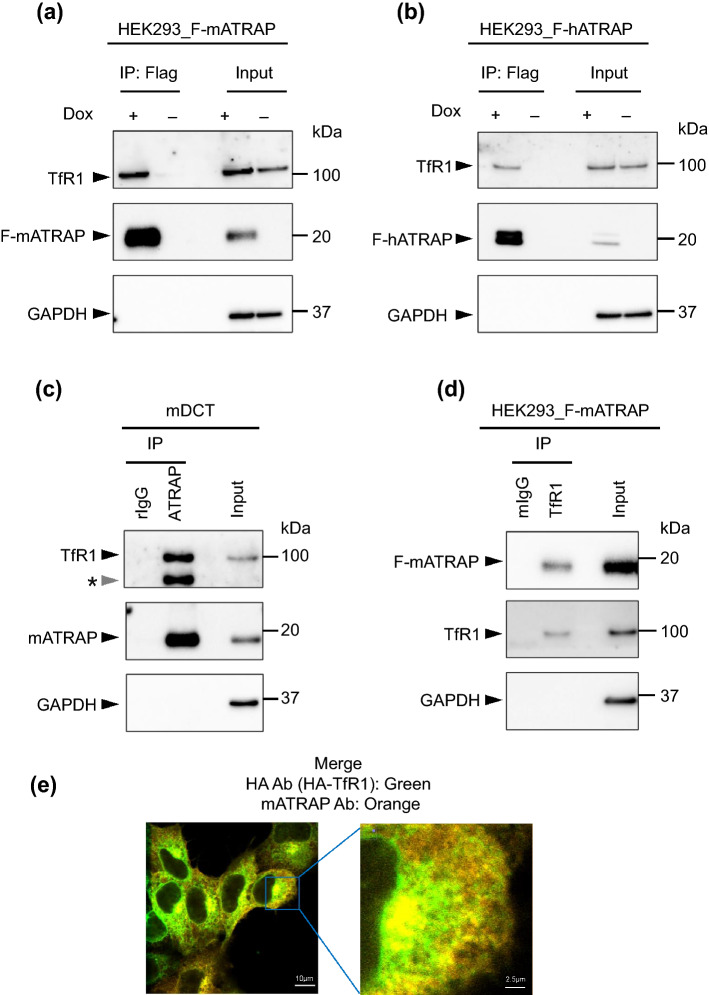
Interaction between ATRAP and TfR1. (**a**–**d**) Western blotting with (**a**) an anti-DYKKKK antibody (Flag) immunoprecipitates from Dox-inducible HEK293_F-mATRAP cells treated with or without Dox (Dox+/−), (**b**) an anti-DYKKKK antibody immunoprecipitates from Dox-inducible F-hATRAP-HEK293 cells (Dox+/−), (**c**) an anti-mATRAP antibody (mATRAP) and normal rabbit IgG (rIgG) immunoprecipitates from mDCT cells, and (**d**) an anti-TfR1 antibody (TfR1) and normal mouse IgG (mIgG) immunoprecipitates from HEK293_F-mATRAP cells (Dox+/−). The immunoprecipitates were analyzed using the indicated antibodies. Asterisk (*) indicates unexpected signal. (**e**) Immunofluorescence staining analysis in HA-TfR1 expressing Dox-inducible HEK293_F-mATRAP cells using anti-HA and -mATRAP antibody with Dox treatment. Merged data is presented. Scale bars: 10 or 2.5 µm. Green: anti-HA (HA-TfR1); Orange: anti-mATRAP. *Ab:* antibody.

To further analyze the association between ATRAP and TfR1, we immunoprecipitated exogenous F-mATRAP from established HEK293 cell line lysates and analyzed the presence of TfR1. As shown in Fig. 3a, endogenous TfR1 co-immunoprecipitated with F-mATRAP when using lysates from Dox-treated (Dox+) cells, but not when using lysates from un-induced (Dox−) cells.

To investigate the evolutionary conservation of the ATRAP–TfR1 association, we performed a similar experiment using Flag human ATRAP (F-hATRAP). The results showed that F-hATRAP also immunoprecipitated with endogenous TfR1 (Fig. 3b). In addition, the endogenous mATRAP co-immunoprecipitated with endogenous mouse TfR1 in mDCT and mouse kidney lysates (Fig. 3c and Supplementary Fig. S2b).

To further characterize this association, endogenous TfR1 was immunoprecipitated using an anti-TfR1 antibody, and the presence of F-mATRAP was analyzed using an anti-mATRAP antibody. Endogenous TfR1 was successfully immunoprecipitated using the anti-TfR1 antibody, but not with control mouse normal IgG, and exogenous F-mATRAP was only co-precipitated and detected following immunoprecipitation with the anti-TfR1 antibody (Fig. 3d).

We examined the cellular colocalization of ATRAP and TfR1. For this purpose, we stably expressed a hemagglutinin (HA)-tagged variant of TfR1 (HA-TfR1) in Dox-treated HEK293_F-mATRAP cells. To this end, Dox-treated HA-TfR1-expressing HEK293_FmATRAP cells were fixed and permeabilized with detergent, and co-stained with an anti-HA antibody and anti-mATRAP antibody. Confocal dissection of immunofluorescent stained HEK293_F-mATRAP showed that F-mATRAP was colocalized with HA-TfR1 in the cytoplasm (Fig. 3e and Supplementary Fig. S2a).

### ATRAP altered intracellular iron levels

**Figure 4:**
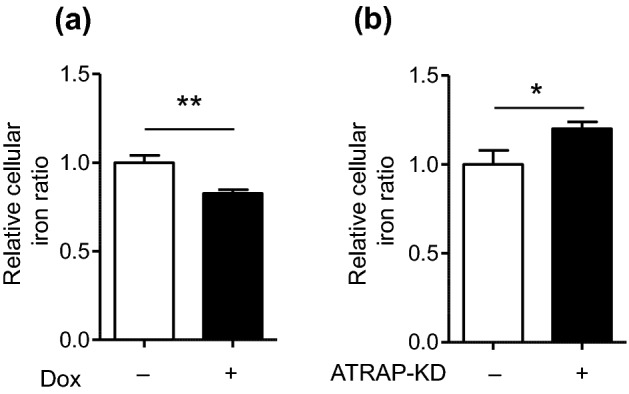
Effects of ATRAP expression on cellular iron levels. Cellular iron levels were assessed by detecting red fluorescence generated using the FerroOrange assay. (**a**) in Dox-inducible HEK293_F-mATRAP cells, with or without Dox treatment (Dox + /-). Unpaired *t*-test: **p < 0.01 vs. Dox+ cells (n = 6). (**b**) in HEK293 cells treated with siRNA targeted ATRAP (ATRAP-KD) or control siRNA (control). Unpaired *t*-test: *p < 0.05 vs. ATRAP-KD cells (n = 6). The data shown are presented as the mean ± SEM.

ATRAP was initially identified as a repressor of pathogenic activation of AT1R. If this phenomenon is a common feature of ATRAP partner molecules, then ATRAP might affect the function of TfR1. Considering that TfR1 is a major receptor of holo-Tf, enhanced-ATRAP expression can be expected to affect cellular iron levels. To test this hypothesis, we analyzed intracellular iron concentrations in the presence or absence of enhanced-F-mATRAP expression. For this, we assessed the relative cellular iron levels using FerroOrange, a cell-permeable fluorescent probe for iron. As shown in Fig. 4a, Dox-inducing enhanced F-mATRAP expression decreased the cellular iron content. In contrast, siRNA-induced ATRAP-knockdown increased iron levels in HEK293 cells (Fig. 4b). Dox treatment did not affect the iron level in original HEK293 cells (Supplementary Fig. S3).

**Figure 5:**
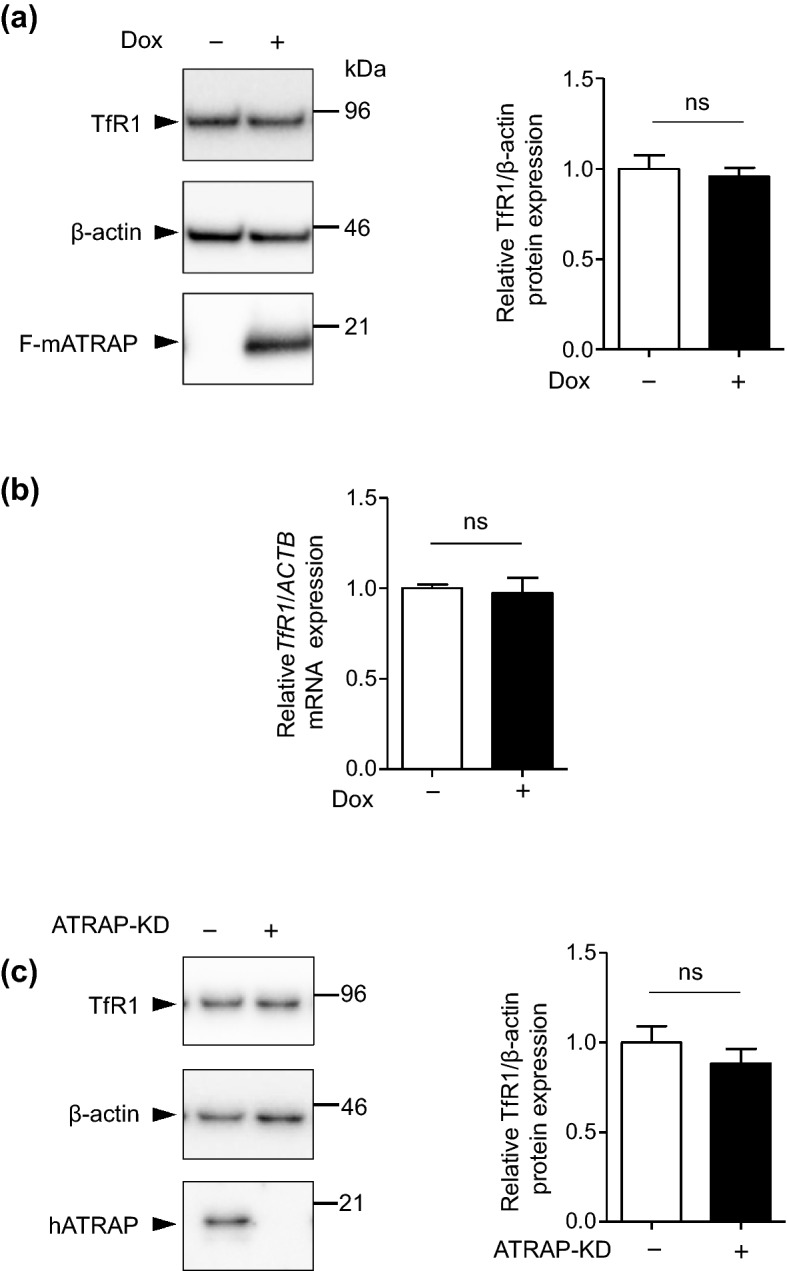
Effects of ATRAP expression on TfR1 expression. (**a**, **c**) Western blot analysis of TfR1 protein expression (**b**) and RT-qPCR analysis of TfR1 mRNA expression (**a**) in Dox-inducible HEK293_F-mATRAP cells, with or without Dox treatment (Dox+/−). (**c**) in HEK293 cells treated with siRNA targeted ATRAP (ATRAP-KD) or control siRNA (control). (**a**, **c**) Representative western blot probed with indicated antibodies are shown in the left panel. Quantitative results are plotted in the right panel. Unpaired *t*-test, (**a**) n = 11 or (**c**) n = 6. The data shown are presented as the mean ± SEM. (**b**) Quantitative results of RT-qPCR are shown. Unpaired *t*-test, n = 3. The data shown are presented as the mean ± SEM.

Because enhanced-F-mATRAP expression decreased the cellular iron levels, we next assessed the cellular expression levels of TfR1 protein and mRNA by immunoblotting and reverse transcription-quantitative polymerase chain reaction (RT-qPCR) analysis, respectively. Unexpectedly, the protein and mRNA abundance of TfR1 was not significantly altered by enhanced-F-mATRAP expression, compared with the corresponding levels in control cells (Fig. 5a,b). Similarly, ATRAP knockdown did not significantly alter TfR1 expression (Fig. 5c).

### ATRAP altered the intracellular localization of TfR1

Because enhanced-F-mATRAP expression decreased cellular iron levels but did not alter TfR1 expression, we hypothesized that the enhanced F-mATRAP expression decreased TfR1 expression on the cell surface.

**Figure 6:**
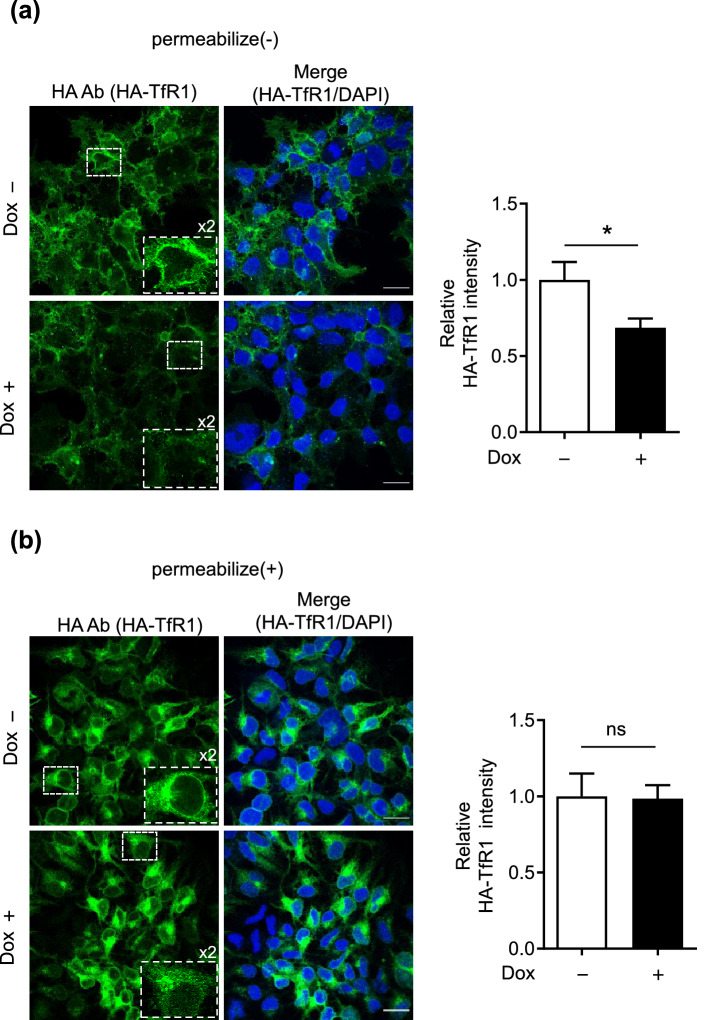
Effects of ATRAP expression on the cell-surface abundance of the TfR1 protein. Immunofluorescence staining analysis of (**a**) cell-surface or (**b**) total HA-TfR1 expression in HA-TfR1 expressing Dox-inducible HEK293_F-mATRAP cells with an anti-HA antibody, with or without Dox treatment (Dox+/−). The cells were fixed with 4% paraformaldehyde without permeabilization (**a**: cell-surface TfR1) or with permeabilization (**b**: total TfR1). Green: HA-TfR1, Blue: DAPI. Scale bars: 20 μm. Quantitative analysis was performed based on the sum of the HA-TfR1-staining intensity/DAPI counts. The data shown in graphs are presented as the mean ± SEM. Unpaired *t*-test: *p < 0.05 vs. Dox + cells (n = 7–8).

To test this hypothesis, we investigated the cell-surface localization of HA-TfR1 by performing immunofluorescence staining. For this purpose, the HA-TfR1 expressing HEK293_F-mATRAP cells treated with or without Dox were fixed under a non-permeabilizing condition (4% paraformaldehyde) and stained with an anti-HA antibody. Our results indicated that Dox-induced F-mATRAP upregulation decreased HA-TfR1 expression on the cell surface (Fig. 6a). However, enhanced F-mATRAP expression showed no apparent alternation of the immunofluorescence signal for HA-TfR1 in detergent-permeabilized cells fixed with 4% paraformaldehyde (Fig. 6b). Together with the result that there was no significant effect of enhanced ATRAP expression on the total TfR1 expression as analyzed using western blotting (Fig. 5a), these results indicate that enhanced F-mATRAP expression decreased the cell-surface expression of TfR1 without altering its overall expression level.

**Figure 7:**
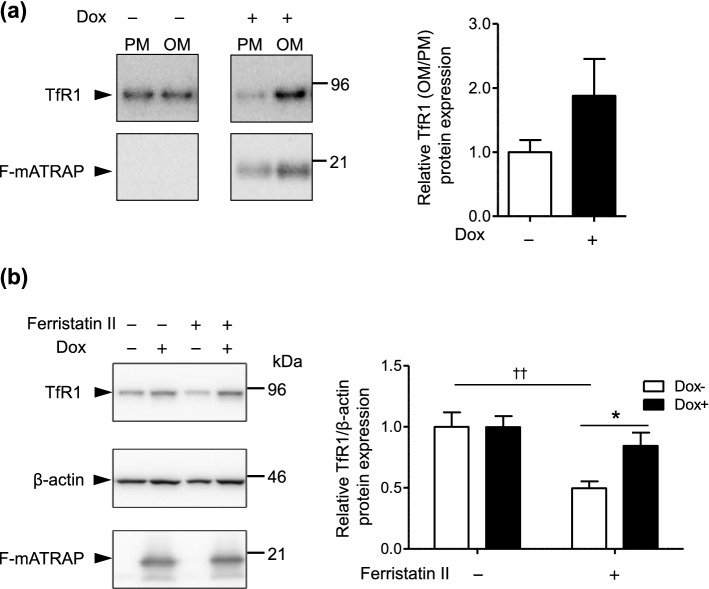
Effects of ATRAP expression on the membrane fractions expression of TfR1 and ferristatin II-induced TfR1 degradation. (**a**) Cell fractionation analysis of the expression of TfR1 in membrane fractions. Plasma membrane (PM) and organelle membrane (OM) fractions were analyzed by western blotting using the indicated anitbodies. Quantitative results are shown in the right panel. n = 2. (**b**) Western blot analysis of TfR1 protein expression following ferristatin II treatment (0 or 10 μM) for 4 h. The HEK293_F-mATRAP cells were cultured with or without Dox (Dox+/−). (n = 5) Total cell lysates were analyzed with the indicated antibodies. Representative western blot results are shown in the top panel. Quantitative results are shown in the right panel. *p < 0.05 vs. ferristatin II; ^††^*p* < 0.01 vs. Dox+ cells, as determined by two-way ANOVA with Bonferroni’s post-hoc test. The data shown are presented as the mean ± SEM.

To confirm these results, we performed a cell fractionation experiment. Appropriate fractionation of the cytoplasm, plasma membrane, and organelle membrane was confirmed by probing samples using antibodies against marker proteins (Supplementary Fig. S6). Then, we analyzed the expression of TfR1 in the plasma membrane and organelle membrane fraction. Consistent with results shown in Fig. 6, enhanced F-mATRAP expression decreased TfR1 expression in the plasma membrane but increased it in the organelle membrane (Fig. 7a).

To further support our hypothesis, we analyzed the effect of Dox-induced F-mATRAP upregulation on ferristatin II-induced TfR1 downregulation. Ferristatin II is a small molecule that acts upon TfR1 at the cell surface and induces TfR1 degradation through the endocytosis–lysosome pathway^22,23^ (Fig. 7b, lanes 1 and 3). Reducing the cell-surface expression of TfR1 by enhanced ATRAP expression would attenuate the effect of ferristatin II in HEK293_F-mATRAP cells. Our results indicate that enhanced F-mATRAP expression inducing a reduction in cell surface TfR1 expression attenuated ferristatin II-induced TfR1 degradation (Fig. 7b, lanes 2 and 4; Supplementary Fig. S7).

Taken together, our results indicate that enhanced ATRAP expression restricted the cell-surface expression of TfR1.

### ATRAP enhancement reduces oxidative stress signaling

**Figure 8:**
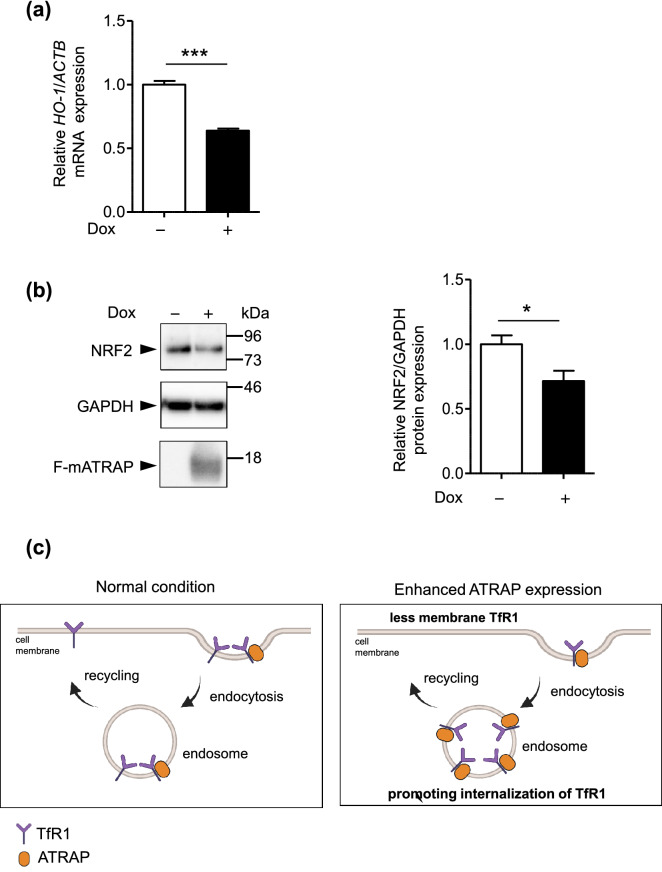
Effects of enhanced ATRAP expression on the oxidative stress signaling. (**a**) RT-qPCR analysis of *HO-1* mRNA expression and (**b**) western blot analysis of NRF2 protein expression in Dox-inducible HEK293_F-mATRAP cells, with or without Dox treatment (Dox+/−). Quantitative results of RT-qPCR are shown. Unpaired t-test: ***p < 0.0001 vs. Dox+ cells. n = 3. (**b**) Representative western blot probed with indicated antibodies are shown. Quantitative results are shown in the right panel. *p < 0.05 vs. Dox+ cells. n = 6. (**a**, **b**) The data shown are presented as the mean ± SEM. (**c**) schematic representation of a model to explain TfR1 cellular trafficking under normal conditions or in the presence of enhanced ATRAP expression. This figure was created at biorender.com.

As described above, enhanced ATRAP expression reduced cellular iron levels, likely through the reduction in cell surface TfR1 expression. The TfR1-mediated cellular iron intake increases ROS levels and induces transcription factor (nuclear factor-erythroid 2-related factor 2, NRF-2) accumulation, which result in the transcription of oxidative stress response genes (heme oxygenase-1, HO-1)^24^. To examine the effect of enhanced F-mATRAP expression on oxidative stress signaling, we analyzed *HO-1* mRNA expression and NRF2 protein expression. The results indicated that enhanced F-mATRAP expression decreased both *HO-1* mRNA and NRF2 protein expression, respectively (Fig. 8a,b).

## Discussion

The results of this study revealed molecular and functional links between ATRAP and TfR1. ATRAP may regulate TfR1 by restricting cell-surface expression of TfR1 by stimulating its internalization, as proposed in the model illustrated in Fig. 8c. Several lines of evidence support this conclusion. Firstly, ATRAP associated with TfR1. Second, enhanced ATRAP expression decreased TfR1 cell-surface expression without altering the overall TfR1-expression levels. Third, enhanced ATRAP expression suppressed the effect of ferristatin II, which targets TfR1 at the cell surface. Consistent with a function of TfR1 in cellular iron uptake, enhanced ATRAP expression reduced the cell-surface expression of TfR1, which corresponded with reduced cellular iron levels and oxidative stress signaling. Taken together, these results suggest that ATRAP most likely promotes TfR1 internalization, resulting in decreased cellular iron levels and oxidative stress signaling.

Our results revealed the functional regulation mechanism of TfR1-mediated iron entry into cells described above. This novel description of the post-translational regulation of TfR1 function, together with the well-characterized iron-inducing translational control of TfR1^11,25^, might maintain iron homeostasis in the kidneys. Iron contributes to kidney fibrosis, which causes CKD through ROS accumulation and mitochondrial damage^26–29^.

Previously, we reported that kidney fibrosis in aged *ATRAP*-KO mice was exacerbated by ROS accumulation and mitochondrial damage^5^. In addition, heterozygous *TfR1*-KO reduced ROS levels and attenuated kidney fibrosis in a mouse model of UUO^13^. However, no significant alternation in iron and TfR1 protein levels was observed in young ATRAP-transgenic (Tg) mouse kidney (Supplementary Figs. S4 and S5). As a limitation of the present study, it is unknown whether the ATRAP–TfR1 interaction is involved in kidney fibrosis, ROS accumulation, and/or mitochondrial damage in aged mice. Hence, we intend to address these questions in future investigations.

An interesting question is how ATRAP promotes TfR1 internalization. TfR1 continuously accepts holo-Tf in the cell culture condition. Briefly, complexes between holo-Tf and TfR1 are internalized into cells through clathrin-mediated endocytosis. Subsequently, the internalized TfR1 is trafficked to early endosomes. After the release of iron in late endosomes, Tf and TfR1 are trafficked to recycling endosomes and recycled back to the cell surface (Fig. 8c left)^30,31^. However, some TfR1 degrades during time spent in the endosome–lysosome pathway^31,32^. Based on the ATRAP-associating proteins identified in this study, our results suggest that clathrin-mediated endocytosis (GO rank 11) and recycling endosome-related factors (GO rank 3 and rank 20) are involved in the endocytic-recycling pathway of TfR1. It is possible that ATRAP may promote endocytosis and/or the inhibited recycling of TfR1 through these ATRAP-associated proteins. We could not rule out the possibility that ATRAP may affect the lysosome-mediated degradation of TfR1. Future analysis is needed to uncover the detailed mechanism of action involved in ATRAP-mediated TfR1 internalization (Fig. 8c right). As we cannot rule out the possibility of an indirect association between ATRAP and TfR1, it will also be important to resolve which proteins directly interact with ATRAP among the identified proteins. Our results related to ATRAP-binding proteins suggest a mechanism whereby ATRAP promotes angiotensin II-induced AT1R internalization. Similar mechanisms related to TfR1 internalization might also exist. As AT1R and TfR1 are functionally relevant in various situations^33–35^, it would be intriguing to resolve the relationship between AT1R and TfR1 together with ATRAP in future studies.

In conclusion, our results revealed that TfR1 is a novel ATRAP-interacting protein. ATRAP may act as a suppressor of TfR1 by facilitating its internalization, which could affect iron metabolism and oxidative stress signaling. This novel ATRAP–TfR1 axis might be a mechanism relevant to the ROS/mitochondrial dysfunction-mediated process of kidney fibrosis.

## Methods

### Constructing an HEK293 cell line that stably expresses mATRAP (HEK293_F-mATRAP)

We constructed pLenti_TetOn_flag-mouse-ATRAP (F-mATRAP, containing blasticidin-resistance gene), flag-human-ATRAP (F-hATRAP, containing blasticidin-resistance gene), and pLenti_HA-human-TfR1 (HA-TfR1, containing puromycin-resistance gene) plasmids. With the TetOn system, Dox can be used to induce the expression of target genes^36^. In this study, dox was used to induce F-mATRAP or F-hATRAP based on the TetOn system. The Flag tag was inserted at the N-terminal of mouse and human ATRAP. An HA tag was inserted between amino acids 111 and 112 of TfR1 (Supplementary Fig. S8). Detailed plasmids maps are available upon request. The Flag epitope was selected because of the availability of a high-affinity monoclonal anti-Flag antibody that enabled sensitive immunoprecipitation and because Flag peptides enable efficient elution of protein complexes^37^.

Lentiviral supernatants were produced as described elsewhere^38^. Briefly, pLenti_TetOn_F-mATRAP or pLenti_HA-TfR1 plasmid (3 µg), pLP1, 3 µg of pLP2/VSVG (3 µg; Thermo Fisher Scientific), and pAdvantage (1.3 µg; Promega) were mixed in Opti-MEM medium (1.5 mL; Thermo Fisher Scientific) and added to Lipofectamine2000 (39.9 μL; Thermo Fisher Scientific) in Opti-MEM (1.5 mL). The resulting solution was mixed and incubated for 20 min at room temperature. While incubating the DNA/Lipofectamine mixture, HEK293FT cells (Thermo Fisher Scientific) (5 × 10^6^) were seeded in a poly-l-lysine-coated 10 cm tissue culture plate. After the incubation, the DNA/Lipofectamine mixtures were added to the HEK293FT cells. At 8 h post-transfection, the medium was exchanged with Dulbecco’s modified Eagle’s medium (DMEM) containing 10% fetal bovine serum (FBS) and 10 µM forskolin. After 24 h, the culture supernatants were collected and filtered through 0.22 µm Steriflip filters (Millipore) to generate the lentiviral supernatants. For the lentiviral infections, the lentiviral supernatant (2 mL) was incubated with HEK293 cells (American Type Culture Collection) or HEK293_F-mATRAP cells (2 × 10^6^) for 24 h. Thereafter, the lentiviral supernatants were discarded followed by the addition of DMEM containing blasticidin (10 μg/mL) (for F-mATRAP) or puromycin (1.5 μg/mL) (for HA-TfR1).

### siRNA and transfection

The following siRNA sequence was used: ATRAP siRNA #1: UACGGUCCUGAGAAGACCC. For the non-silencing control, AllStars Negative Control siRNA (Qiagen) was used. siRNA transfections were performed in a 12-well plate with Lipofectamine RNAiMax Reagent (Thermo Fisher Scientific) according to the manufacturer’s protocol. siRNA-treated HEK293 cells were used in the subsequent experiments after 48 h.

### Immunoprecipitation

HEK293_F-mATRAP cells or transiently transfected HEK293 cells with the F-hATRAP plasmid were treated with or without Dox (0.5 μg/mL; 631311, Clontech Laboratories) and incubated overnight. Cells not treated with Dox served as a negative control. The cells were lysed with T-lysis buffer (20 mM HEPES NaOH, 150 mM NaCl, 0.05% Tween-20, 2.5 mM MgCl_2_, 1 mM dithiothreitol [DTT], protease inhibitor cocktail [Nakarai], and phosphatase inhibitor cocktail [Nakarai]). After centrifugation at 15,000×*g* for 10 min at 4 °C, the supernatants were incubated for 2 h at 4 °C with anti-DDDDK tag Ab-magnetic beads (clone FLA-1, M185-11, MBL). After the incubation, the beads were washed three times with T-wash buffer (20 mM HEPES NaOH, 150 mM NaCl, 0.05% Tween-20, 2.5 mM MgCl_2_, and 1 mM DTT), and the bound proteins were eluted from the beads with wash buffer containing Flag peptide (0.1 mg/mL; DYKDDDDK, F3290, Sigma-Aldrich). The eluates were subjected to sodium dodecyl sulfate–polyacrylamide gel electrophoresis (SDS-PAGE) for Oriole staining, LC–MS/MS analysis, and western blot analysis.

For endogenous mATRAP or human TfR1, mDCT cells, provided by Dr. Peter A. Friedman (University of Pittsburgh School of Medicine) or HEK293_F-mATRAP cells (treated with Dox (1 μg/mL) overnight) were lysed with T-lysis buffer, and the lysates were incubated for 1 h at 4 °C with anti-mATRAP antibody or normal rabbit IgG (negative control for mATRAP) (1.5 μg; 2027, Santa Cruz Biotechnology), anti-TfR1 antibody (3 μg; 13–6800, Thermo Fisher Scientific) or normal mouse IgG (negative control for TfR1) (3 μg; 2025, Santa Cruz Biotechnology). Thereafter, the lysates were incubated with Protein G-Dynabeads (12 μL; 10003D, Thermo Fisher Scientific) for 1 h or 40 min at 4 °C. The beads were washed three times and eluted with SDS sample buffer containing DTT (50 mM) and subjected to SDS-PAGE and western blot analysis.

All experiments were performed at least three times, and representative results are shown.

### LC–MS/MS analysis

LC–MS/MS was performed by Medical Proteoscope. The eluates were subjected to SDS-PAGE (5–20% SuperSep Ace, Fujifilm Wako Pure Chemical), and the proteins were detected using silver staining (299-58901, Fujifilm Wako Pure Chemical). Next, the protein bands were excised from the silver-stained gel, subjected to trypsin hydrolysis^39^, and sequenced by MS using an Ultimate 3000 RSLCnano and Q Exactive Orbitrap (Thermo Fisher Scientific). All the MS/MS samples were analyzed using Mascot software (Matrix Science), which identified 822 proteins. Then, we excluded proteins with a peptide count of < 1 and protein contaminants. In addition, the identified proteins that did not show an increased abundance after inducing ATRAP expression were considered unlikely to interact with ATRAP; therefore, those with an expression ratio of ≤ 2 were excluded. In this analysis, proteins with an expression level of 0 were converted to 4000, which was less than the minimum abundance of control precipitates to take the ratio. GO analysis was performed using the Metascape database, which was a web-based portal that provides comprehensive gene-list annotation and analysis resources^40^.

### Ferristatin II-induced TfR1-degradation assay

HEK293_F-mATRAP cells were seeded at a density of 1.5 × 10^5^ cells/well in a 12-well plate in 1 mL of DMEM with 10% FBS, with or without Dox (3 μg/mL). The next day, the cells were washed with FBS-free medium and treated with ferristatin II (0, 10, or 20 μM; C1144, Sigma-Aldrich). After a 4 h or 24 h incubation, we collected the lysate proteins using sample buffer containing SDS (1%).

### Iron assay

HEK293_F-mATRAP cells or siRNA-treated HEK293 cells were seeded at a density of 1 × 10^4^ cells/well in a 96-well plate in DMEM (100 μL) with FBS (10%), with or without Dox (3 μg/mL). The next day, the cells were washed with HBSS three times, and FerroOrange working solution (1 μmol/L) was added to the cells. Next, the cells were incubated at 37 °C for 30 min, and the fluorescence was measured using excitation wavelengths of 530 and 590 nm with a synergyLX instrument (Biotek Instruments).

### Western blot analysis

Western blot analysis was performed as described elsewhere^38^. Briefly, total protein was extracted from cells using a sample buffer containing SDS (1%). Then, the protein concentration in each sample was measured with a Qubit 2.0 Fluorometer (Thermo Fisher Scientific). An equal amount of each protein extract was resolved on a 5–20% polyacrylamide gel (ATTO Corporation) and electrophoresed. After separation, the proteins were transferred to a polyvinylidene fluoride membrane. The membranes were blocked for over 1 h at room temperature with Tris-buffered saline with Tween (TBST) containing skim milk (5%) and probed overnight at 4 °C with specific primary antibodies. Antibodies against the following proteins were used: mATRAP and hATRAP (1:1000–3000 dilution, rabbit, developed in this study; Supplementary Fig. S1), Flag M2-horseradish peroxidase (HRP) (1:2000 dilution, A8592, mouse, Sigma-Aldrich), β-actin (1:5000 dilution, A5441, mouse, Sigma-Aldrich), glyceraldehyde 3-phosphate dehydrogenase (GAPDH) (1:5,000 dilution, 2118, rabbit, Cell Signaling Technology), TfR1 (1:3000 dilution, 13–6800, mouse, Thermo Fisher Scientific), and NRF2 (1:2000 dilution, GTX103322, rabbit, GeneTex),TBP (1:2000 dilution, ab63766, Abcam), Rab4 (1:2,000 dilution, BD51-9002091, BD Biosciences). The membranes were washed and further incubated with an appropriate secondary antibody for 60 min at room temperature. When detecting ATRAP, GAPDH, NRF2 or TBP, anti-Rabbit IgG, HRP Linked Whole Ab (NA934-1ML, donkey, GE Healthcare), was diluted 1:2000–5000 with TBST containing skim milk (5%). When detecting TfR1, β-actin or Rab4, anti-Mouse IgG, HRP Linked Whole Ab (NA931-1ML, sheep, GE Healthcare) was diluted 1:1000–5000. When performing western blot analysis of the immunoprecipitated samples, we used TrueBlot anti-rabbit IgG HRP (eb182, mouse, Rockland Immunochemicals) and light-chain-specific anti-mouse IgG (115-005-174, goat, Jackson ImmunoResearch) as secondary antibodies. The bands were visualized using Luminata classico/forte (Merck) or ImmunoStar LD (Fujifilm Wako Pure Chemical) as the enhanced chemiluminescence substrate. The resulting images were quantitatively analyzed using a LAS-4000 Image Analyzer (Fujifilm) or a Chemidoc Touch Imager (Bio-Rad Laboratories). All experiments were performed at least three times, and representative results are shown.

### Immunofluorescence

For immunofluorescence analysis, HEK293_F-mATRAP or HEK293_F-mATRAP_HA-TfR1 cells (1.5 × 10^5^) were cultured on glass coverslips in a 12-well plate with or without Dox (3 μg/mL) for 2 days. The cells were fixed with paraformaldehyde (4%) for 15 min at room temperature, with or without permeabilization with phosphate-buffered saline containing Triton X-100 (0.02%) for 15 min, and then they were blocked with bovine serum albumin (3%) for 2 h at room temperature. The cells were incubated with antibodies against mATRAP (1:600 dilution, rabbit, developed in this study) and the HA epitope (11867423001, 1:600 dilution, clone 3F10, rat, Sigma-Aldrich) at 4 °C overnight. For visualization, the cells were incubated with a secondary Alexa 647-conjugated anti-rabbit IgG antibody (1:1000 dilution, A21245, goat, Thermo Fisher Scientific), a secondary Alexa 488-conjugated anti-rat IgG antibody (1:1000 dilution, A11006, goat, Thermo Fisher Scientific), and 4,6-diamidino-2-phenylindole (DAPI, 1:1000 dilution, Thermo Fisher Scientific) for 2 h at room temperature. The cells were then mounted with ProLong Gold (Thermo Fisher Scientific) and photographed with a ZEISS LSM 700 Laser Scanning Microscope. All images were generated using a single confocal section. All experiments were performed at least three times, and representative results are shown.

### RT-qPCR analysis

Total RNA was extracted from HEK293 cells using the NucleoSpin RNA Plus Kit (Takara Bio), and complementary DNA was produced using the SuperScript III First-Strand Synthesis System (Thermo Fisher Scientific). RT-qPCR was performed with a Bio-Rad CFX96 Touch Real-Time PCR Detection System (Bio-Rad Laboratories), and the reverse-transcribed products were incubated with THUNDERBIRD Next SYBR qPCR Mix (Toyobo). The *TfR1* and *HO-1* mRNA levels were normalized to those of *ACTB*. The following primers were used: *TfR1* (forward: 5′-GCTGCTGCAGAAAAGCTGTT-3′, reverse: 5′-CTGGGCCCCAACTACAACAT-3′), *HO-1* (forward: 5′-GTGCCACCAAGTTCAAGCAG-3′, reverse: 5′-CAGCTCCTGCAACTCCTCAA-3′), *ACTB* (forward: 5′-GCCGCCAGCTCACCAT-3′, reverse: 5′-TCGTCGCCCACATAGGAATC-3′).

### Cell fractionation assay

The cell fractionation assay was performed according to the protocol of the Minute Plasma Membrane Protein Isolation Kit (SM-005, Invent Biotechnologies). In brief, HEK293_F-mATRAP cells were seeded in a 10 cm plate in DMEM with 10% FBS, with or without Dox (3 μg/mL). The next day, cultured cells were lysed in *buffer A*, and the protein concentration was measured with a Qubit 2.0 Fluorometer. An equal amount of each protein extract (partially collected as the whole cell lysate, W) was placed in a filter cartridge. After centrifugation, the supernatant was collected as the cytoplasmic fraction (C) and the pellet was resuspended in *buffer B* and centrifuged. The pellet was collected separately as the organelle membrane protein fraction (OM). The supernatant was then centrifuged again, and the pellet was collected as the plasma membrane protein fraction (PM). Each sample was subjected to SDS-PAGE and western blot analysis.

### Animals

This study was performed in accordance with the National Institutes of Health Guidelines for the Use of Experimental Animals. The animal experiments were approved by the Animal Studies Committee of Yokohama City University (approval number FA20-027) and were conducted in compliance with the ARRIVE guidelines. The mice were housed in a controlled environment under a 12-h light–12-h dark cycle at 25 °C. The mice had free access to food and water. Systemic ATRAP-KO mice and HA-tagged-mATRAP Tg mice in the C57BL/6J background were generated as previously described^41–43^. We used 10–18-week-old male mice.

### Statistical analysis

Statistical analyses were performed with GraphPad Prism (GraphPad Software). All data are shown as the mean ± standard error of the mean (SEM). Differences were analyzed using the following statistical tests. Two-way analysis of variance (ANOVA), followed by Bonferroni’s post-hoc analysis, was performed to determine differences between the control- and Dox-treated groups (Fig. 7b). An unpaired t-test was used to analyze differences between two groups (Figs. 4a,b, 5a–c, 6a,b, 7a, 8a,b, Supplementary Figs. S3, S4, S5). P values < 0.05 were considered statistically significant.

## Supplementary Information

Supplementary Table S1.

Supplementary Information.

## Data Availability

The data presented in this study are available on request from the corresponding author.
