# Author Correction: Angiotensin II type-1 receptor-associated protein interacts with transferrin receptor-1 and promotes its internalization

**DOI:** 10.1038/s41598-022-25880-1

**Published:** 2022-12-09

**Authors:** Eriko Abe, Akio Yamashita, Keigo Hirota, Takahiro Yamaji, Kengo Azushima, Shingo Urate, Toru Suzuki, Shohei Tanaka, Shinya Taguchi, Shunichiro Tsukamoto, Tatsuki Uehara, Hiromichi Wakui, Kouichi Tamura, Hidehisa Takahashi

**Affiliations:** 1 Department of Medical Science and Cardiorenal Medicine, Yokohama City University Graduate School of Medicine, Yokohama, Japan; 2 Department of Molecular Biology, Yokohama City University Graduate School of Medicine, Yokohama, Japan; 3 Department of Investigative Medicine Graduate School of Medicine, University of the Ryukyus, Okinawa, Japan; 4 Cardiovascular and Metabolic Disorders Program, Duke-NUS Medical School, Singapore, Singapore

Correction to: *Scientific Reports* https://doi.org/10.1038/s41598-022-22343-5, published online 17 October 2022

The original version of this Article contained an error in Figure 7, where the label of the Y-axis ‘Relative TfR1 (OM/PM) protein expression’ in panel (a) was incorrectly given as ‘Relative TfR1 (PM/OM) protein expression’. The original Figure 7 and accompanying legend appear below.

**Figure 7:**
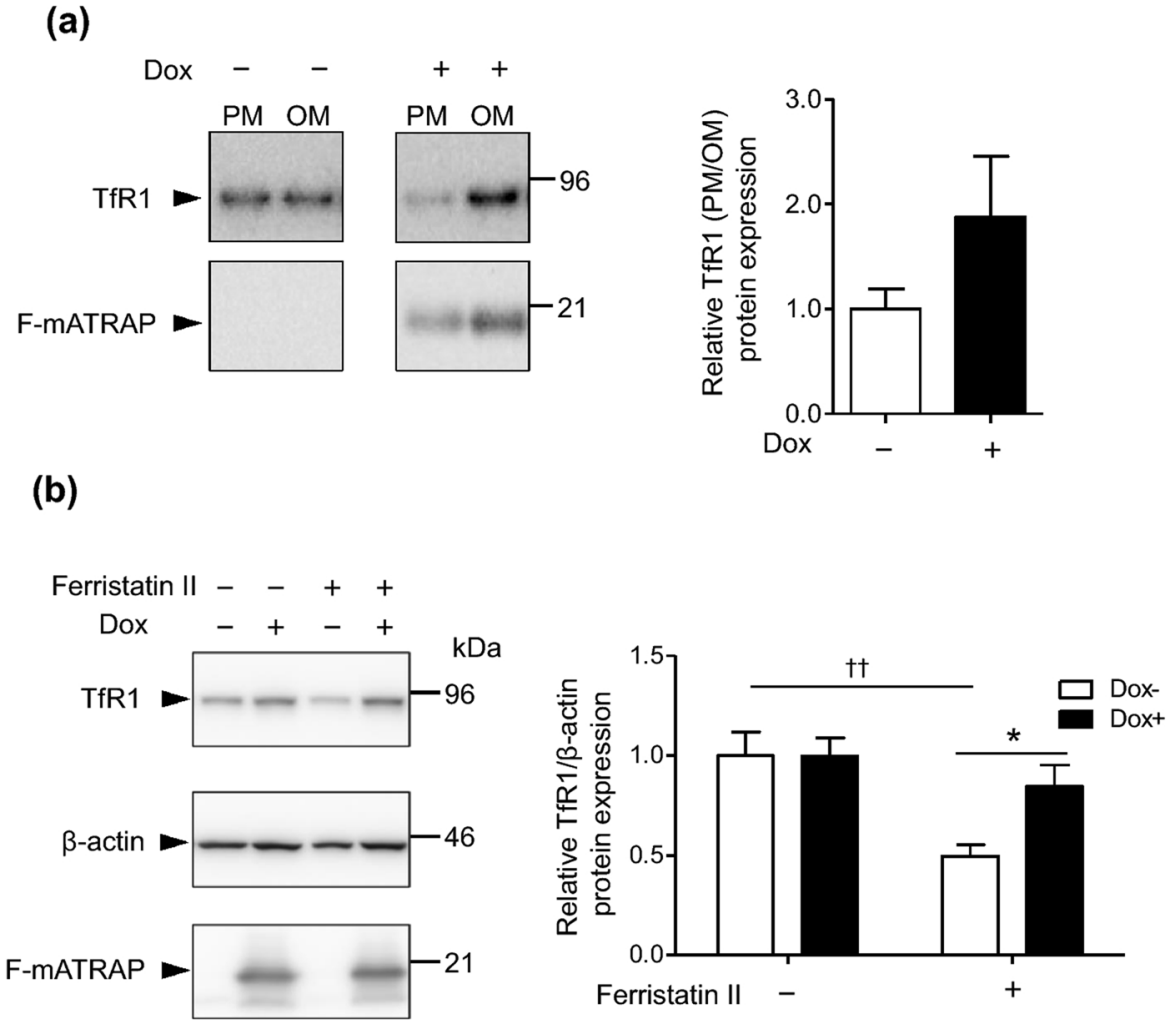
Effects of ATRAP expression on the membrane fractions expression of TfR1 and ferristatin II-induced TfR1 degradation. (**a**) Cell fractionation analysis of the expression of TfR1 in membrane fractions. Plasma membrane (PM) and organelle membrane (OM) fractions were analyzed by western blotting using the indicated anitbodies. Quantitative results are shown in the right panel. n = 2. (**b**) Western blot analysis of TfR1 protein expression following ferristatin II treatment (0 or 10 μM) for 4 h. The HEK293_F-mATRAP cells were cultured with or without Dox (Dox + / −). (n = 5) Total cell lysates were analyzed with the indicated antibodies. Representative western blot results are shown in the top panel. Quantitative results are shown in the right panel. *p < 0.05 vs. ferristatin II; ^††^*p* < 0.01 vs. Dox + cells, as determined by two-way ANOVA with Bonferroni’s post-hoc test. The data shown are presented as the mean ± SEM.

The original Article has been corrected.

