# Supplementary material for: Angiotensin II type-1 receptor-associated protein interacts with transferrin receptor-1 and promotes its internalization
Source: Sci Rep. 2022 Oct 17;12:17376. doi: 10.1038/s41598-022-22343-5 (PMC9576747; doi:10.1038/s41598-022-22343-5)

Supplementary methods

Immunohistochemical analysis. Mouse kidney tissues were fixed overnight in 4% paraformaldehyde. Paraffin-embedded kidney tissues were cut at a thickness of 4 μm. The sections were incubated with an anti-mATRAP antibody, and detection was achieved using the Histofine Kit (Nichirei Biosciences) and staining with 3,3′-diaminobenzidine.

Immunoprecipitation. For endogenous mATRAP or mouse TfR1, mouse kidney tissues were lysed with T-lysis buffer, and the lysates were incubated for overnight at 4°C with anti-mATRAP antibody or normal rabbit IgG (negative control) (3 μg; 2027, Santa Cruz Biotechnology). Thereafter, the lysates were incubated with Protein G-Dynabeads (50 μL; 10003D, Thermo Fisher Scientific) for 1 h at 4°C. The beads were washed three times and eluted with SDS sample buffer containing DTT (50 mM) and subjected to SDS-PAGE and western blot analysis.

Iron level assay (in vivo). To measure iron level in mouse kidney tissues, we used an iron assay kit (Sigma MAK025). The mouse kidney tissue (20 mg) was mechanically homogenized in iron assay buffer and centrifuged at 16,000 × g for 10 min at 4°C. After adding iron probe and incubating for 60 min at room temperature, we measured the absorbance of the samples at 593 nm with a Synergy LX instrument (Biotek Instruments). Iron standard was used for analysis.

Supplementary FigureS1

(a)

Human ATRAP
MELPAVNLKV ILLGHWLLTT WGCIVFSGSY AWANFTILAL GVWAVAQRDS IDAISMFLGG **60**
LLATIFLDIV HISIFYPRVS LTDTGRFGVG MAILSLLLKP LSCCFVYHMY RERGGELLVH **120**
TGFLGSSQD RSAYQTIDSA EA**PADPFAVP EGR**SQDARGY **159**

Mouse ATRAP
MELPAVNLKV ILLVHWLLTT WGCLVFSSSY AWGNFTILAL GVWAVAQRDS IDAIGMFLGG **60**
LVATIFLDII YISIFYSSVA TGDTGRFGAG MAILSLLLKP FSCCLVYHMH RERGGELPLR **120**
PDFFGPSQEH SAYQTIDSSS DAAADP**FASL ENKGQAVPRG Y** **161**

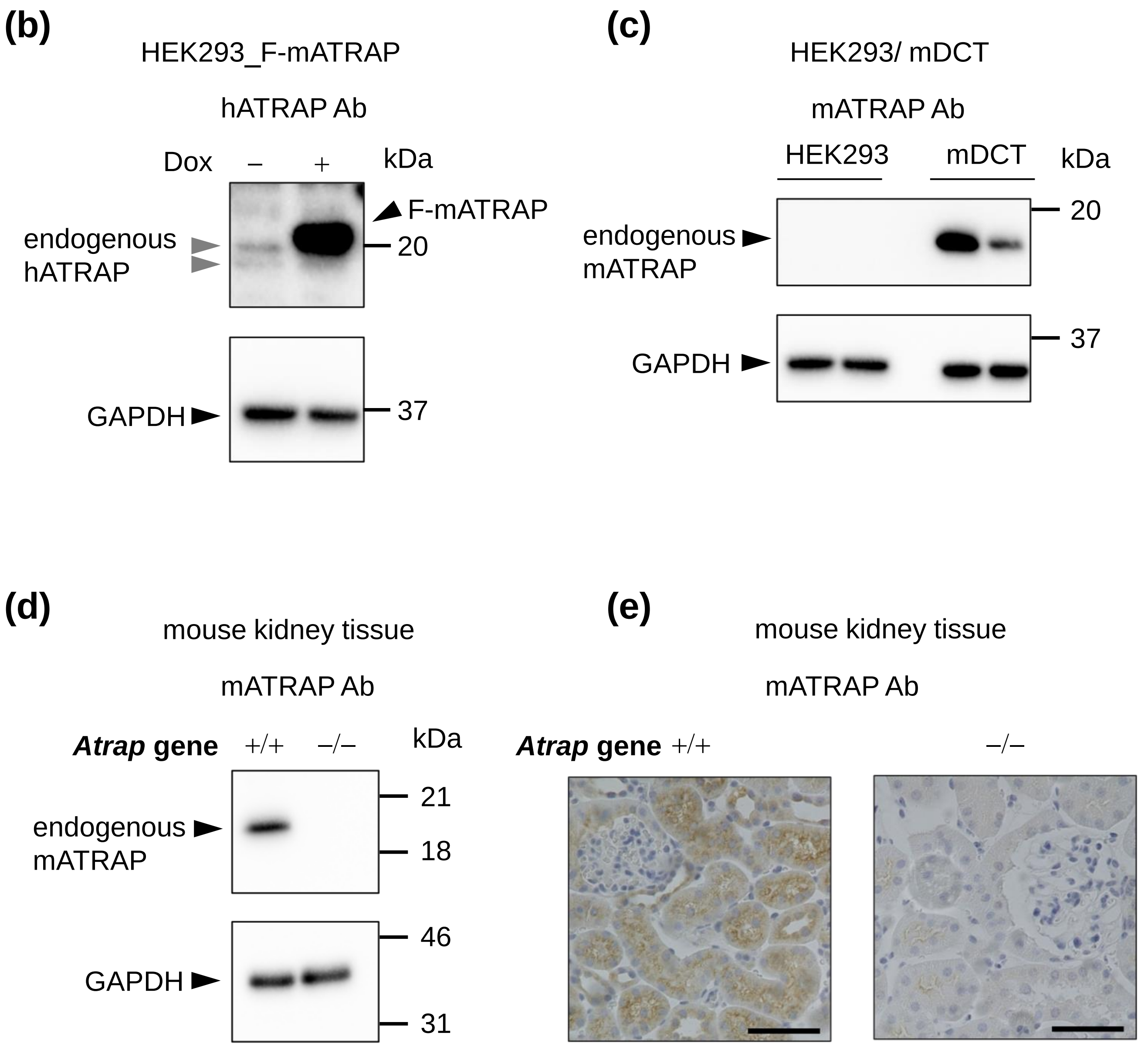

Supplementary FigureS1

Specificity of an anti-mATRAP antibody developed in this study.

(**a**) Amino acid sequences of human and mouse ATRAP. The epitopes for the anti-hATRAP and anti-mATRAP antibodies are indicated with bold and underlined text. (**b**) Total cell lysates from HEK293_F-mATRAP cells, treated with or without Dox (Dox+/-), were analyzed with an anti-hATRAP antibody. The anti-hATRAP antibody detected both endogenous hATRAP and exogenous F-mATRAP. (**c**) Total cell lysates from human (HEK293) and mouse (mDCT) cells were analyzed with an anti-mATRAP antibody. (**d**) Total extracts from mouse kidneys of *ATRAP*-wild type (+/+) and systemic *ATRAP*-KO (-/-) mice were probed with an anti-mATRAP antibody. The membrane of mATRAP was stripped and reprobed for GAPDH. The membrane was incubated in reblot solution for 20 min and blocked for 30 min at room temperature with TBST-containing skim milk (5%) and probed overnight at 4°C with anti-GAPDH antibody (Reblot plus mild solution, 2502, Millipore). (e) Immunohistochemical analysis of mATRAP expression in kidney tissues from *Atrap*-wild type (+/+) and systemic *Atrap*-KO (-/-) mice. Positive mATRAP staining was evidenced by the presence of brown dots. Scale bars: 50 μm

Supplementary FigureS2

(a)

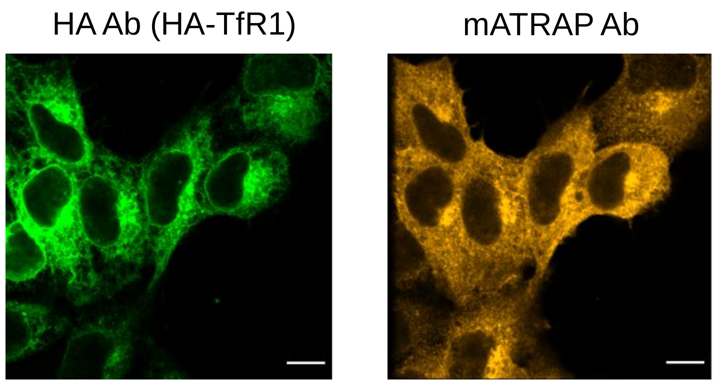

(b)

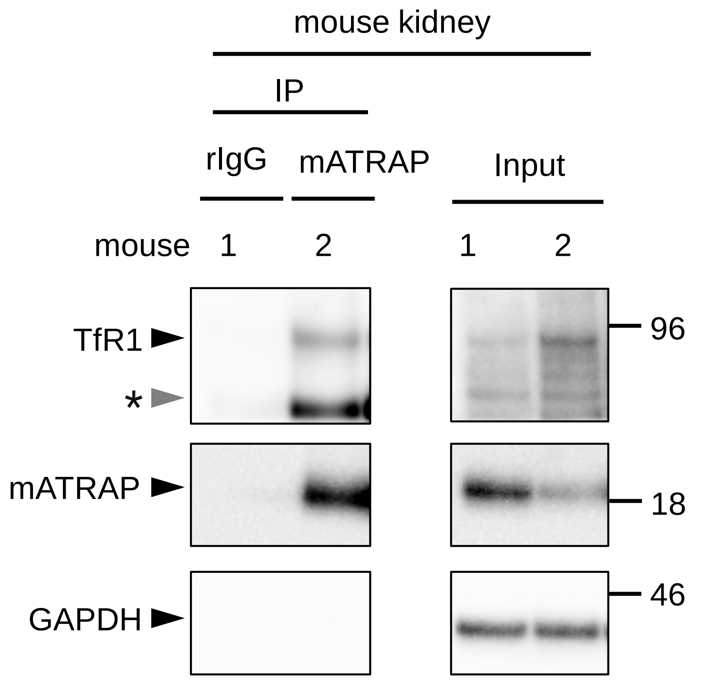

Interaction between ATRAP and TfR1. Related to figure 3

(**a**) Immunofluorescence staining analysis in Dox-inducible HEK293_F-mATRAP cells using an anti-mATRAP antibody and an anti-HA antibody with Dox treatment. Scale bars: 10 µm. Green: aniti-HA (HA-TfR1); Orange: anti-mATRAP. Ab; antibody

(**b**) Western blotting with an anti-mATRAP antibody immunoprecipitates and normal rabbit IgG (rIgG) from mice kidney. The immunoprecipitates were analyzed using the indicated antibodies. Asterisk (*) indicates unexpected signal.

Supplementary FigureS3

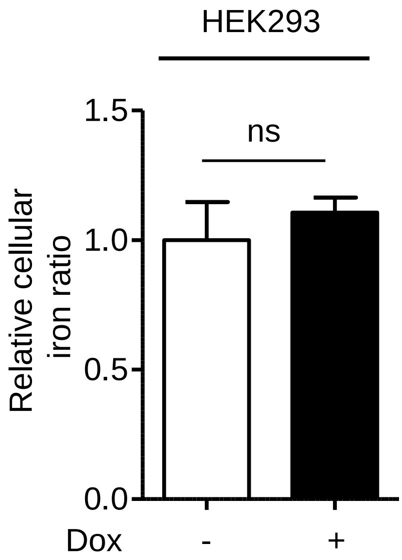

Cellular iron levels in Dox-treated original HEK293 cells. Related to Figure 4.

Cellular iron levels was assessed in original (non-transgene) HEK293 cells, with or without Dox treatment (Dox+/-). Unpaired *t*-test (n = 3). The data are shown as mean ± SEM.

Supplementary FigureS4

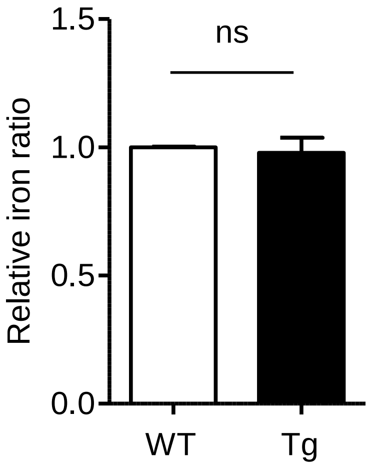

Iron levels in mouse kidney. Related to Figure 4.

Iron levels of ATRAP-wild type (WT) and ATRAP-transgenic (Tg) mice kidney were determined by measuring absorbance using iron assay kit. Unpaired *t*-test (n = 3). The data shown are presented as the mean ± SEM.

Supplementary FigureS5

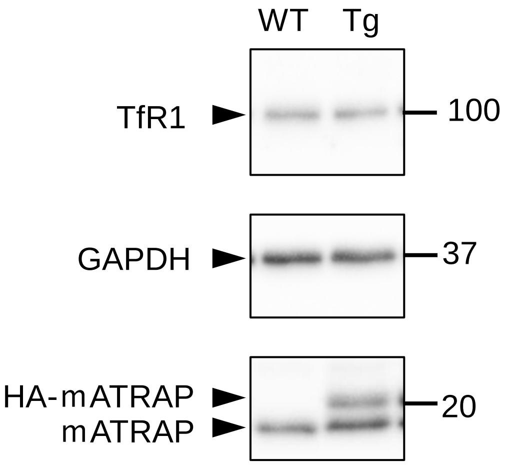

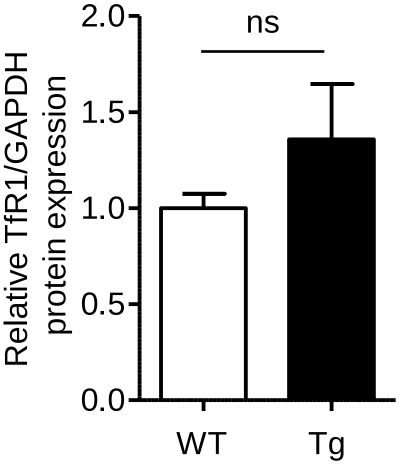

TfR1 expression in ATRAP-wild type (WT) and ATRAP-transgenic (Tg) mouse kidney. Related to Figure 5.

Western blot analysis of TfR1 protein expression in WT and Tg mouse kidney. Representative western blot results are shown in the top panel. Quantitative results are plotted in the bottom panel. Unpaired *t*-test (n = 4). The data shown are presented as the mean ± SEM.

Supplementary FigureS6

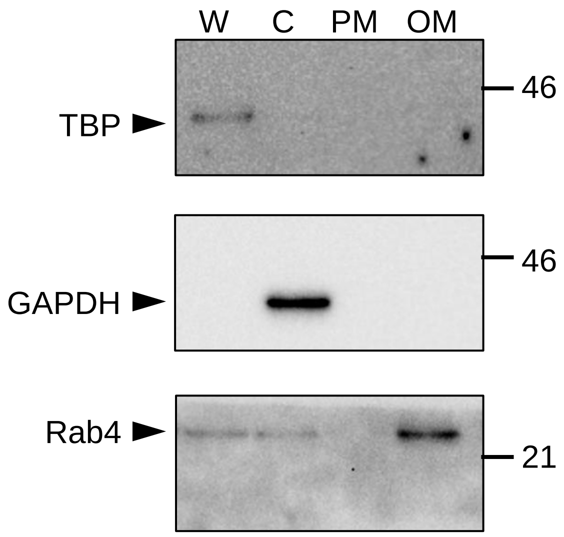

Supplementary Figure S6. Western blotting from cell fractionation assay .Related to Figure 7a.

Western blotting of whole cell lysate (W), cytoplasmic fraction (C), plasma membrane protein fraction (PM), and organelle membrane protein fraction (OM) from HEK293_F-mATRAP cells. The samples were probed with anti-TBP (nuclear marker protein), GAPDH (cytoplasmic marker protein), and Rab4 (early endosome marker protein) antibodies.

Supplementary FigureS7

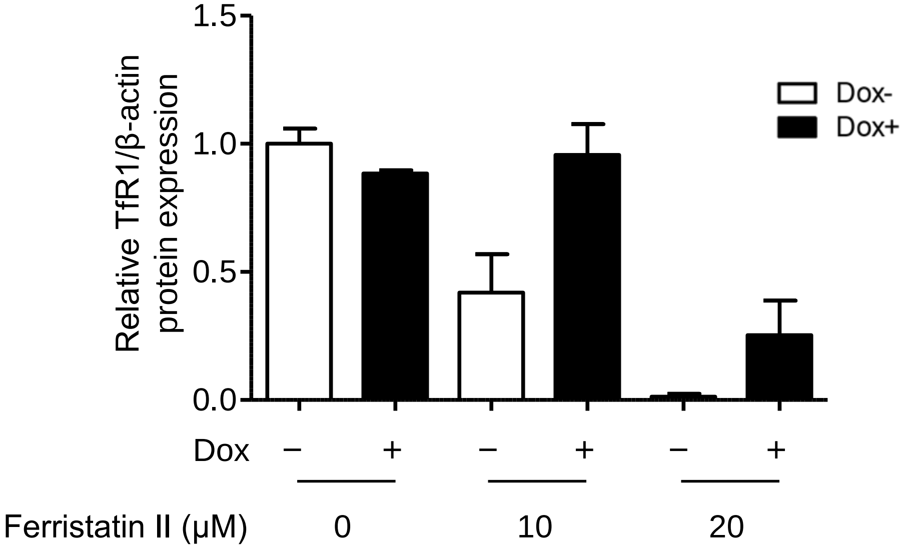

Effects of enhanced ATRAP expression on ferristatin II-induced TfR1 degradation. Related to Figure 7b.

Total cell lysates from F-mATRAP HEK293, treated with or without Dox (Dox+/-), following ferristatin II treatment (0, 10, or 20 μM) for 24 h were probed with an anti-mATRAP antibody. The western blot results for TfR1 protein expression were quantified and plotted (n = 2).

Supplementary FigureS8

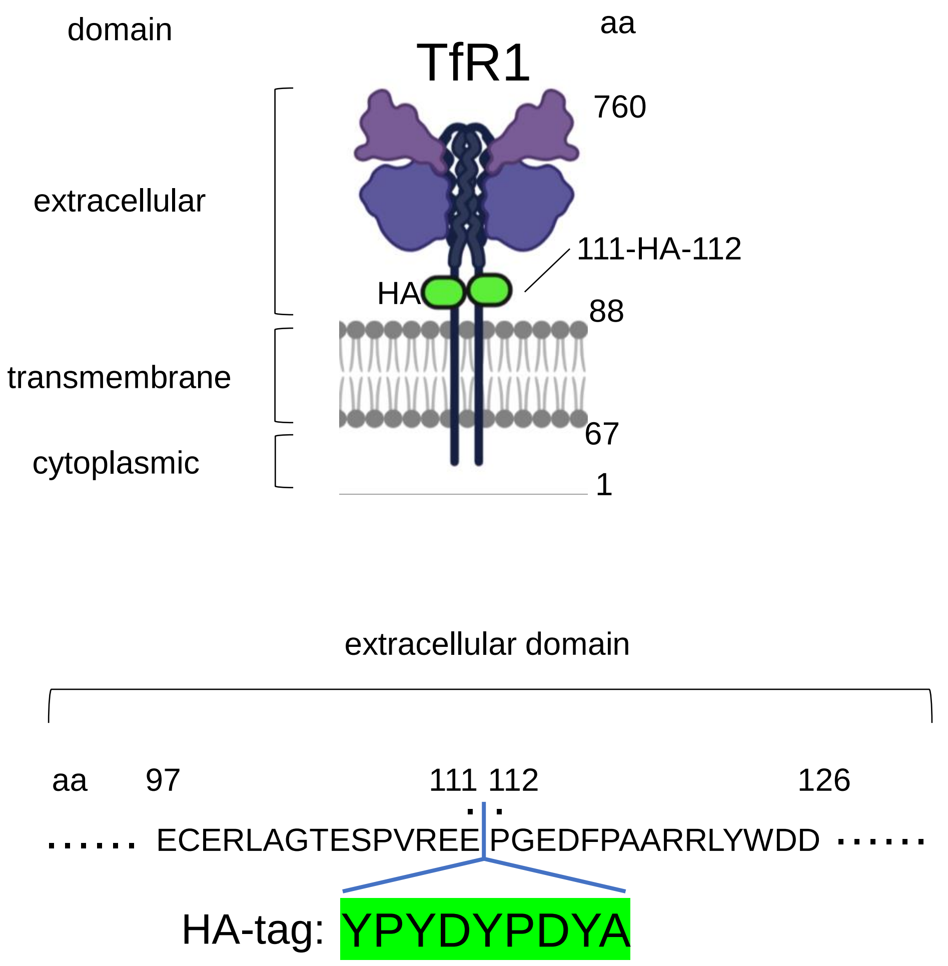

Schema of HA-tagged-TfR1.

Schematic replantation of HA-TfR1 (top) and detailed sequence of HA tag-inserted region (bottom). Amino acid (aa) numbers 1-67 and 88-760 indicate cytoplasmic region and extracellular region of TfR1, respectively. HA-tag was inserted between aa 111 and 112. This figure was created at biorender.com.

Supplementary data

A whole gel of Figure1a

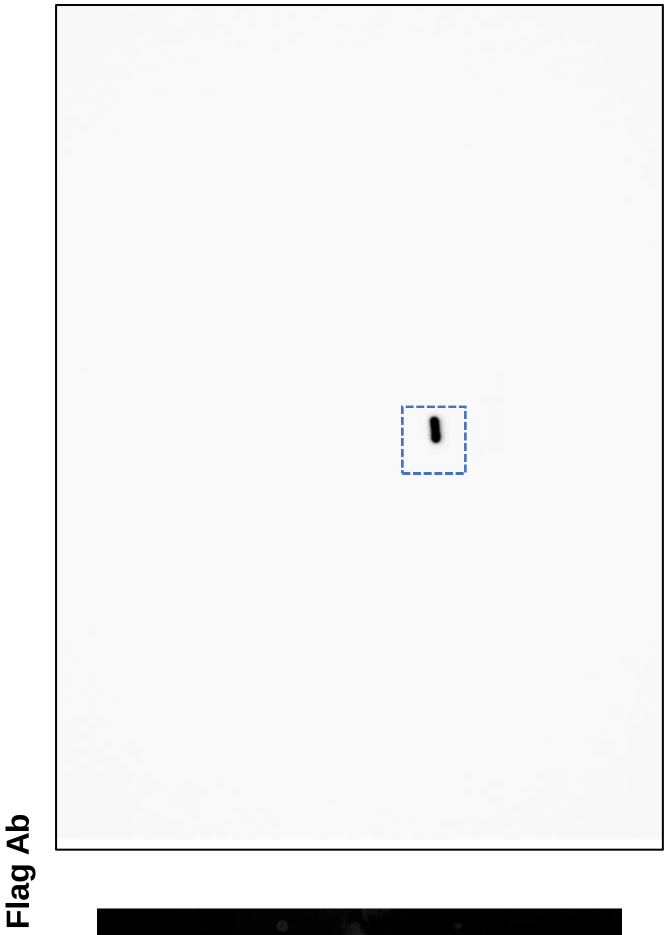

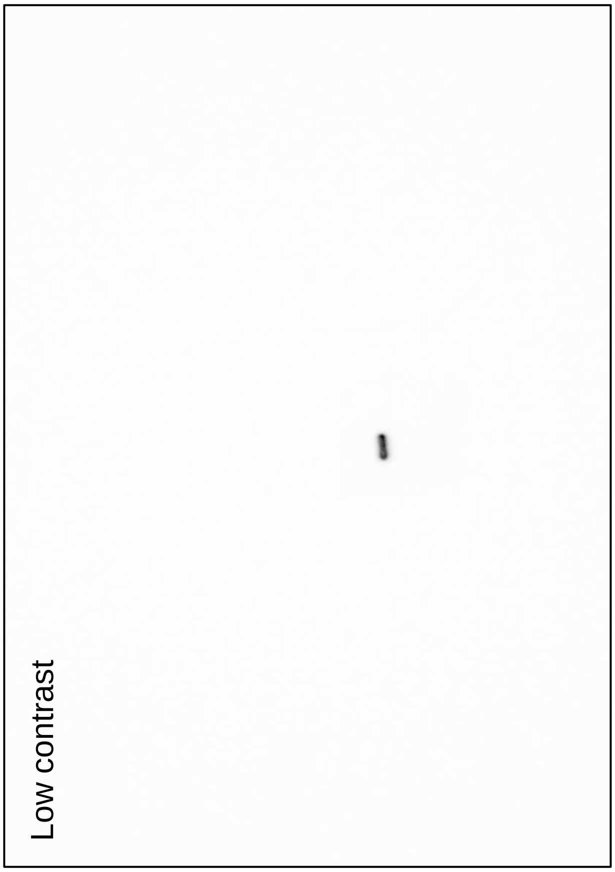

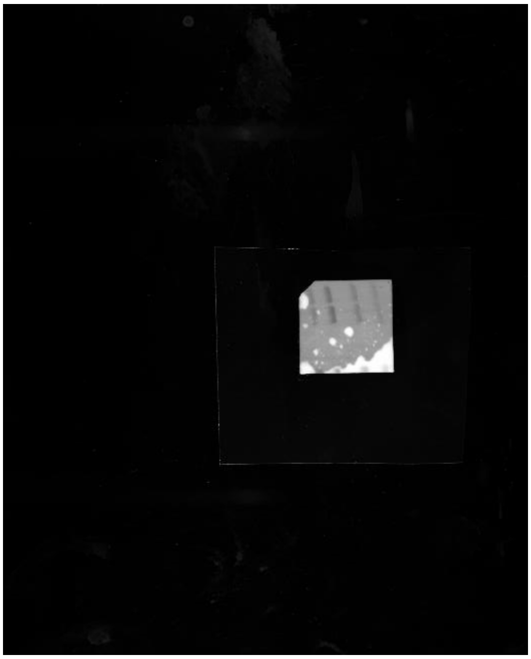

Supplementary data

A whole gel of Figure1a

mATRAP Ab

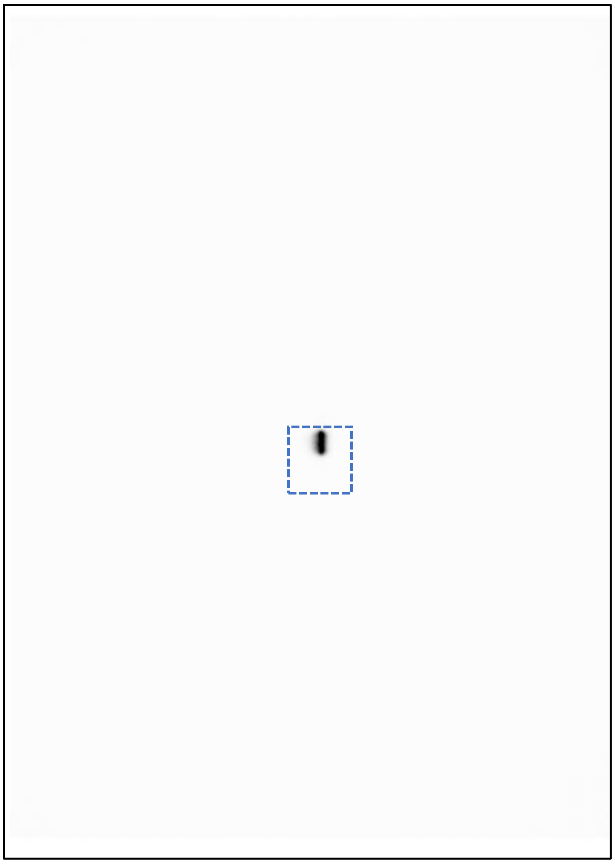

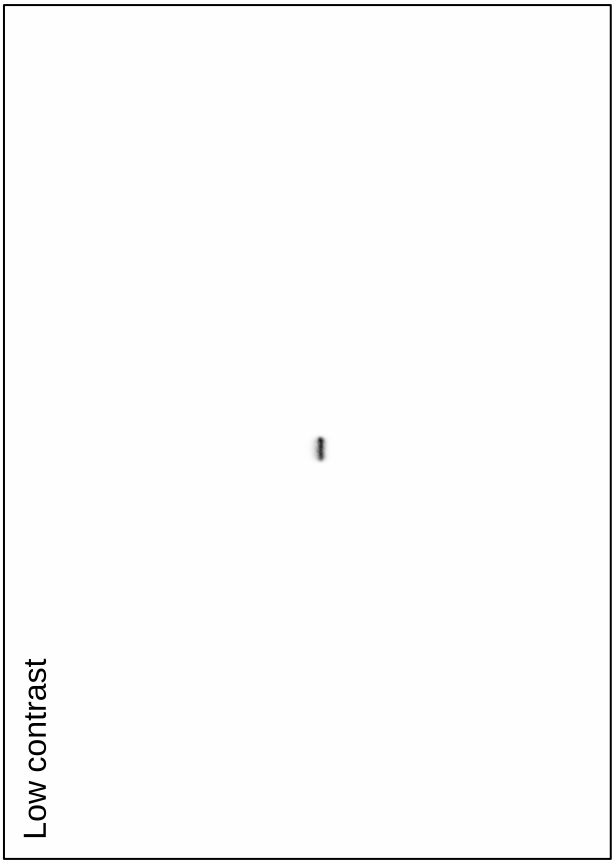

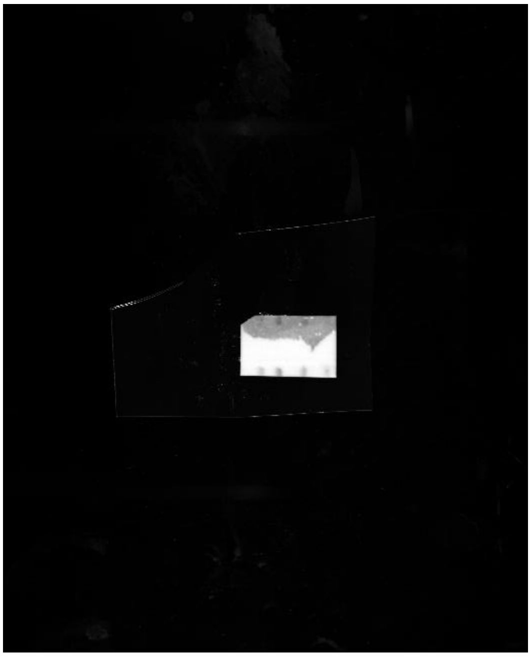

Supplementary data

A whole gel of Figure1a

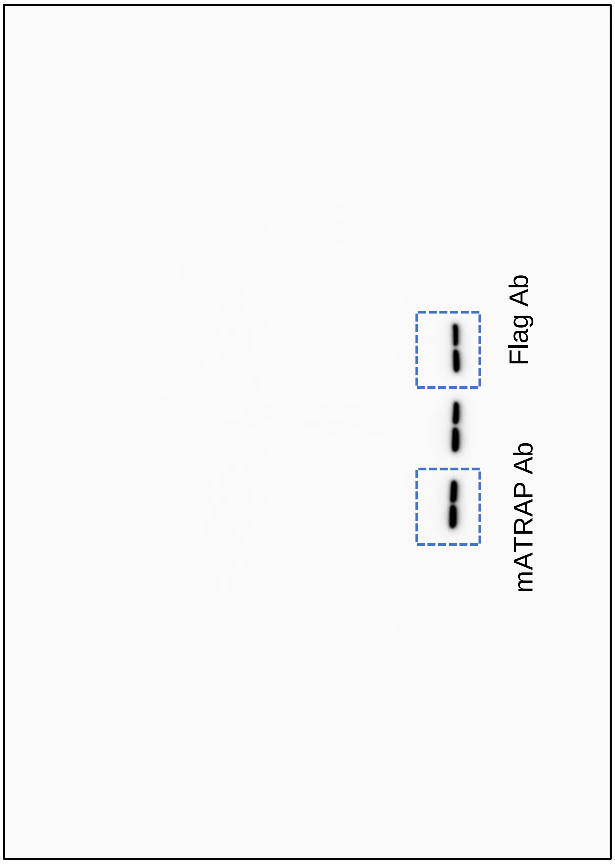

GAPDH

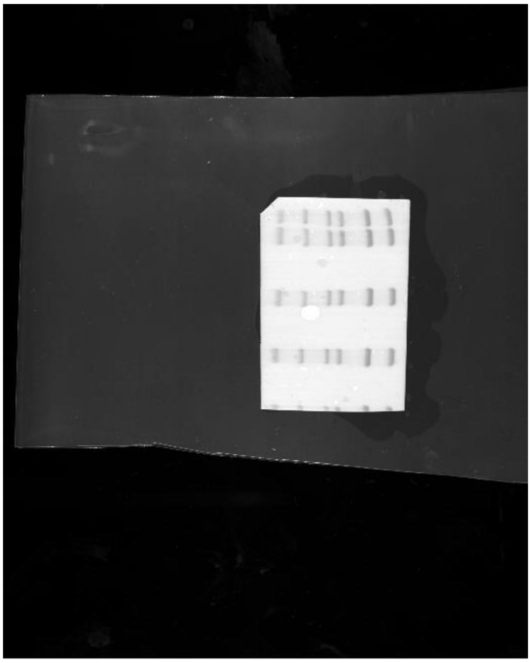

Supplementary data

A whole gel of Figure3a

TfR1

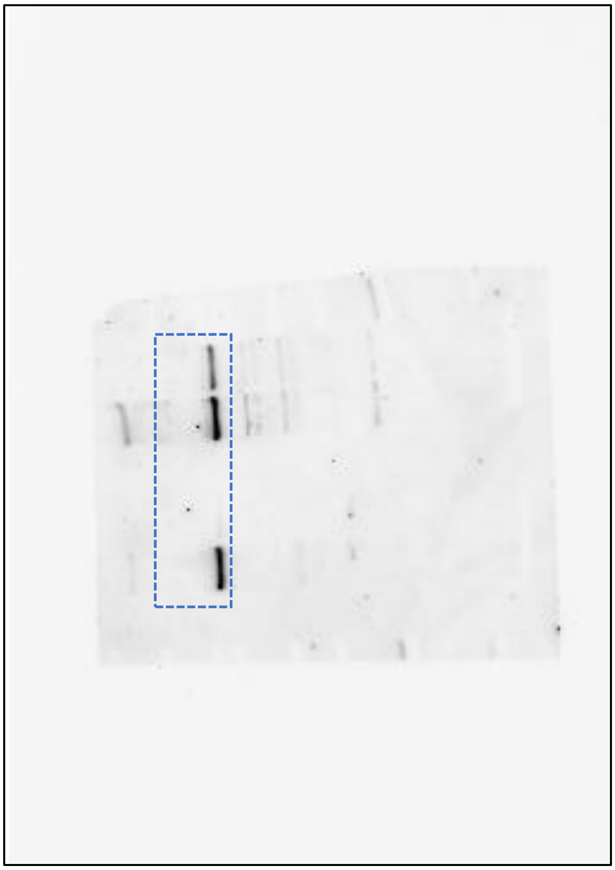

F-mATRAP

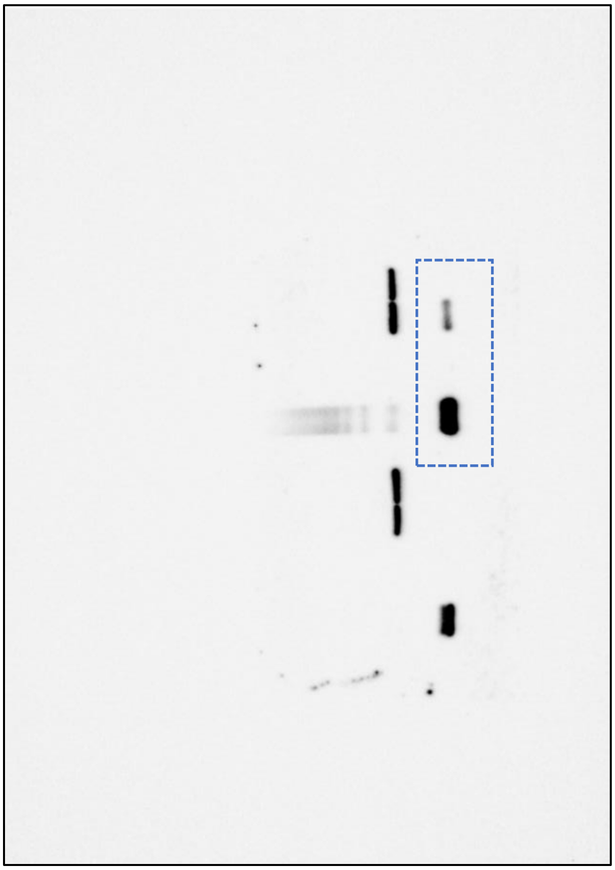

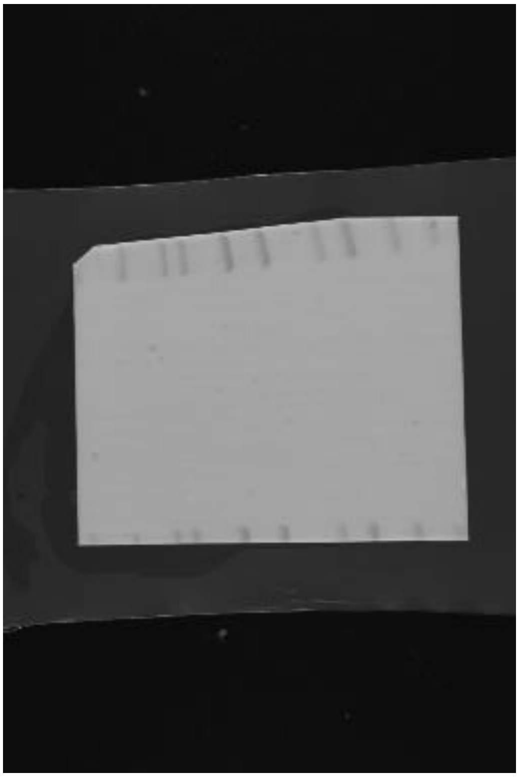

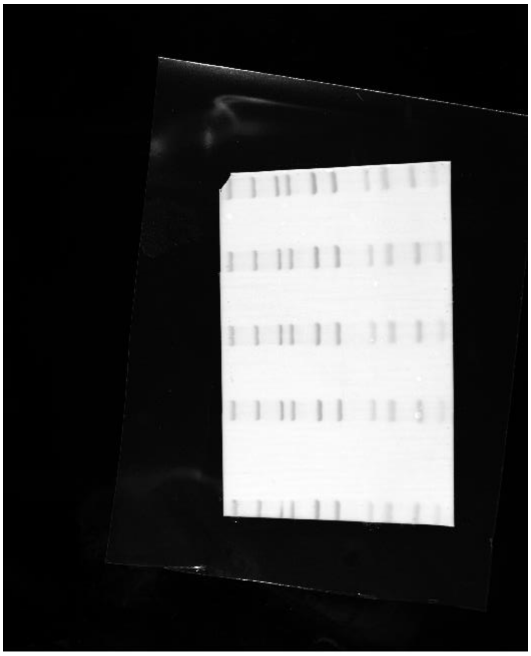

Supplementary data

A whole gel of Figure3a

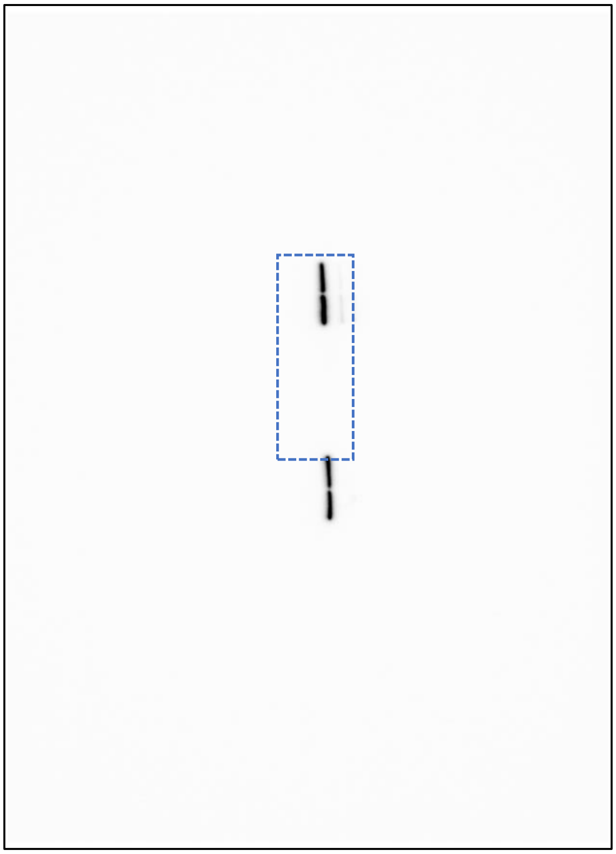

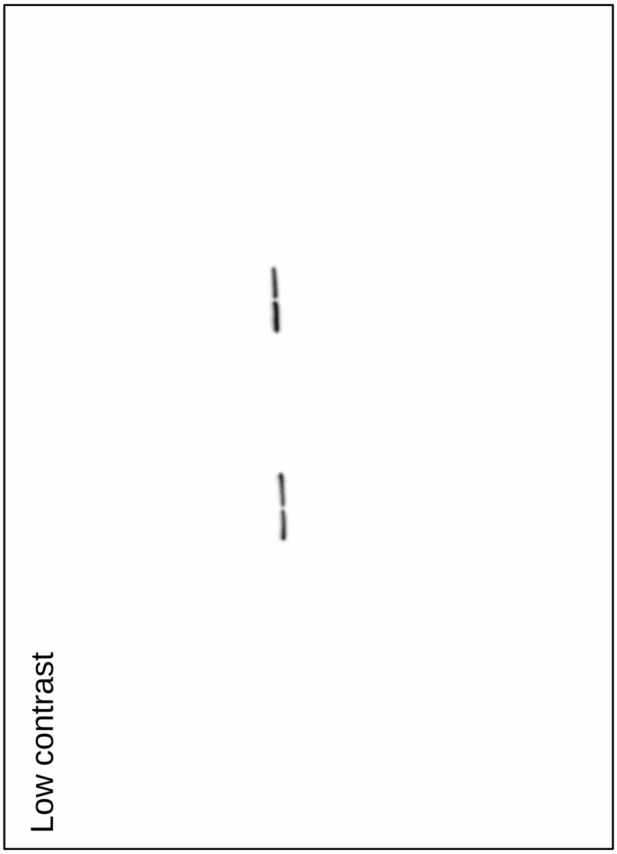

GAPDH

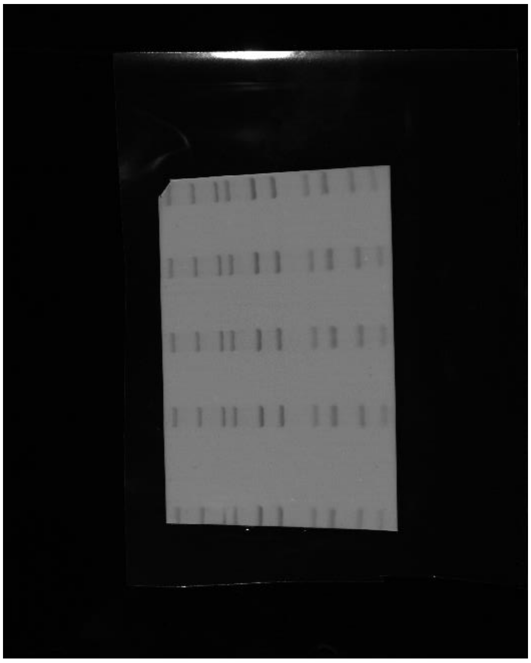

Supplementary data

A whole gel of Figure3b

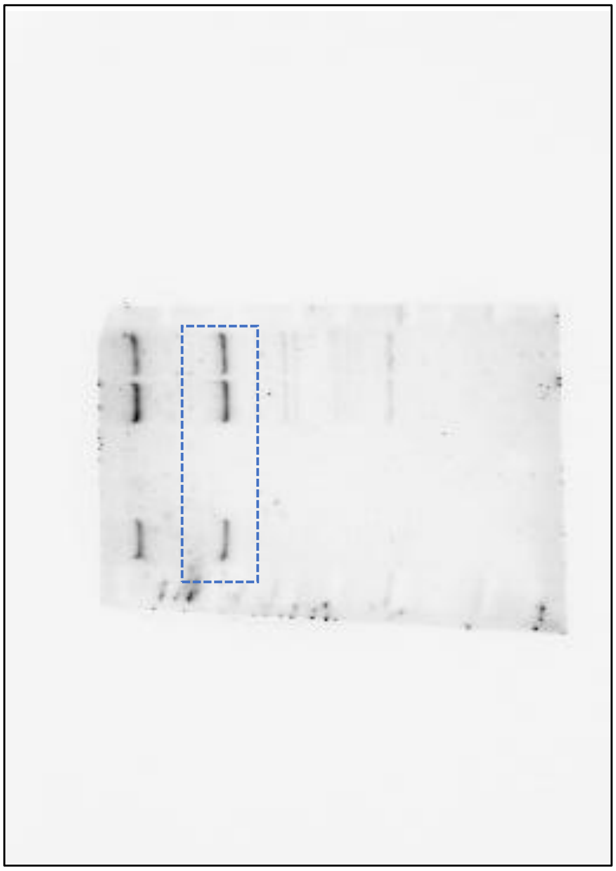

TfR1

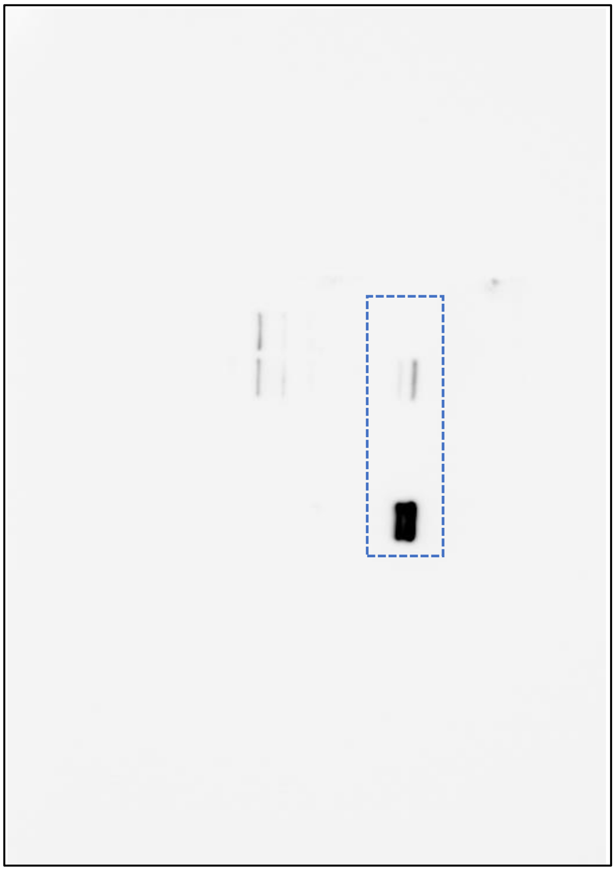

F-hATRAP

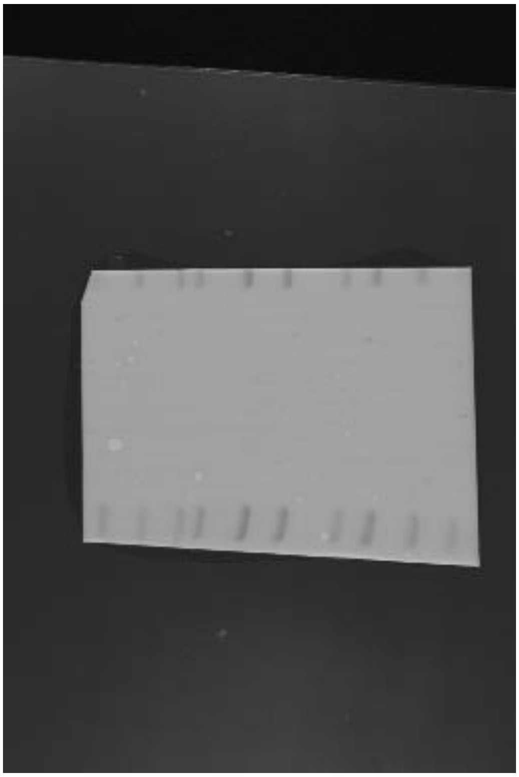

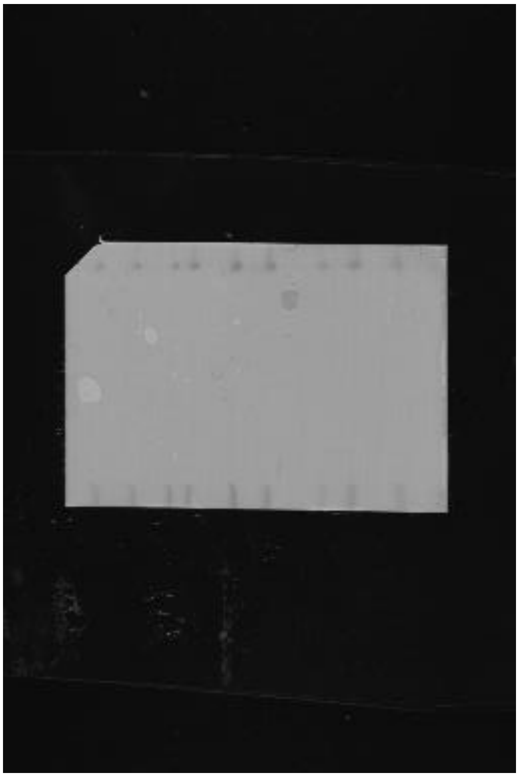

Supplementary data

A whole gel of Figure3b

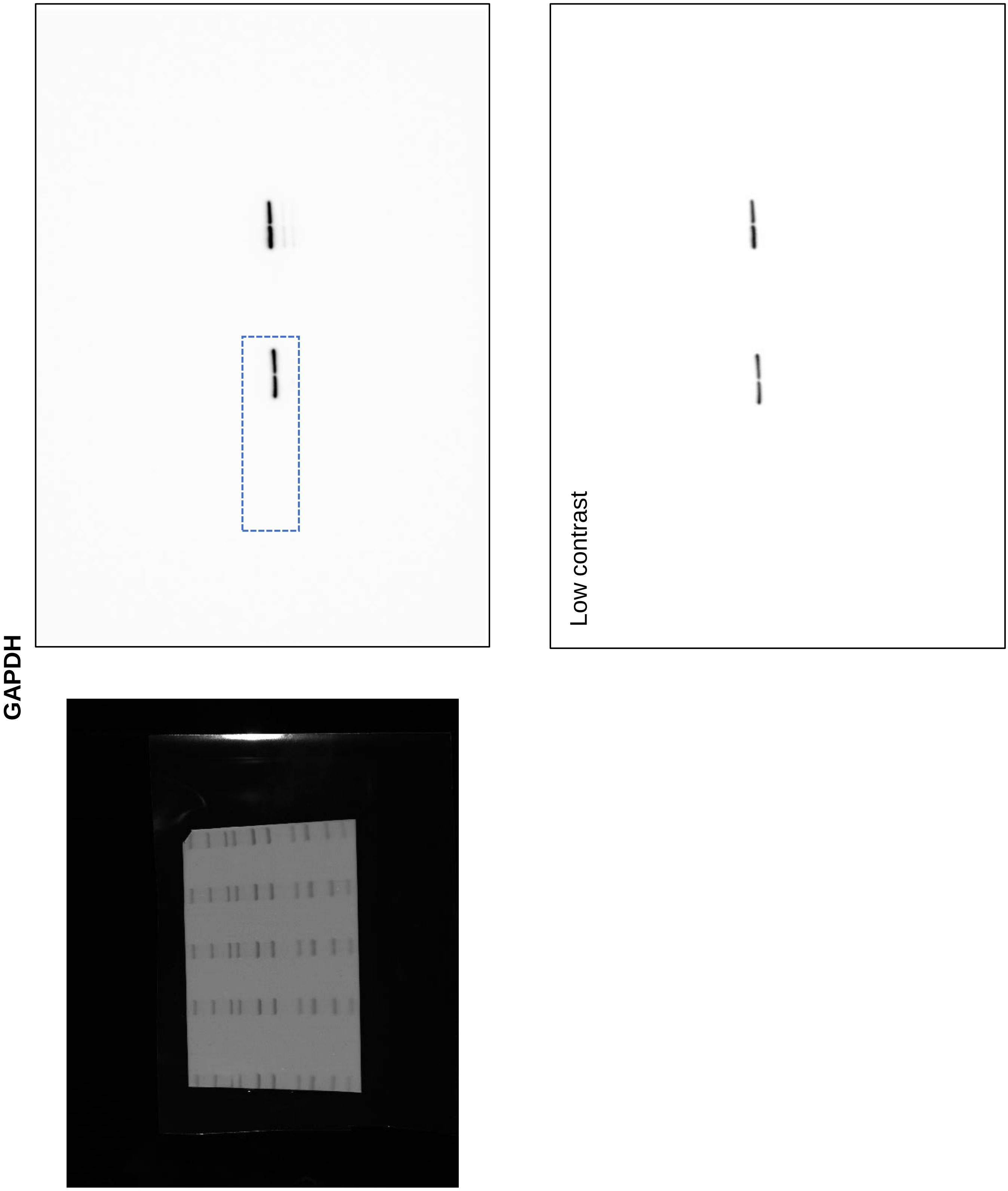

Supplementary data

A whole gel of Figure3c

TfR1

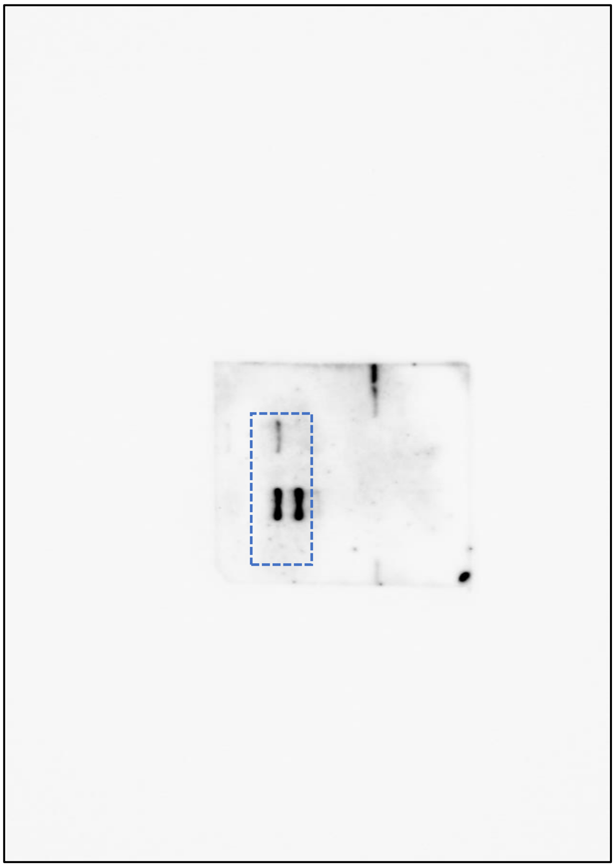

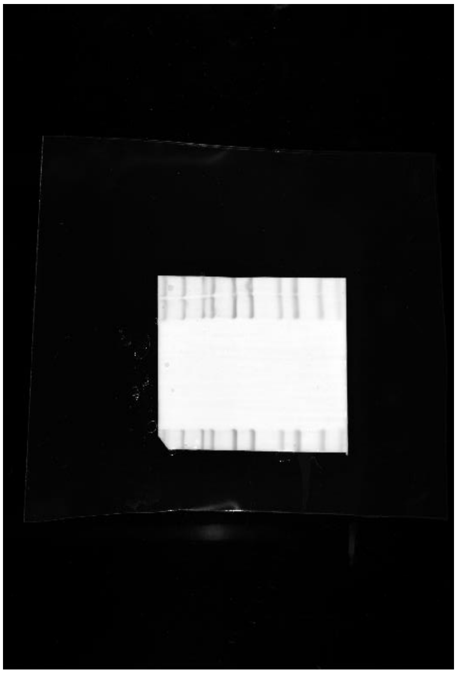

F-mATRAP

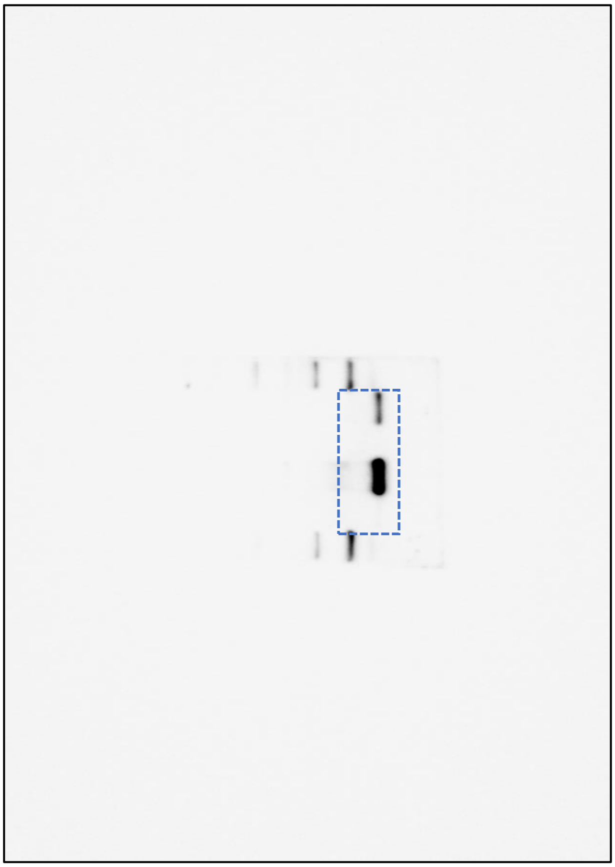

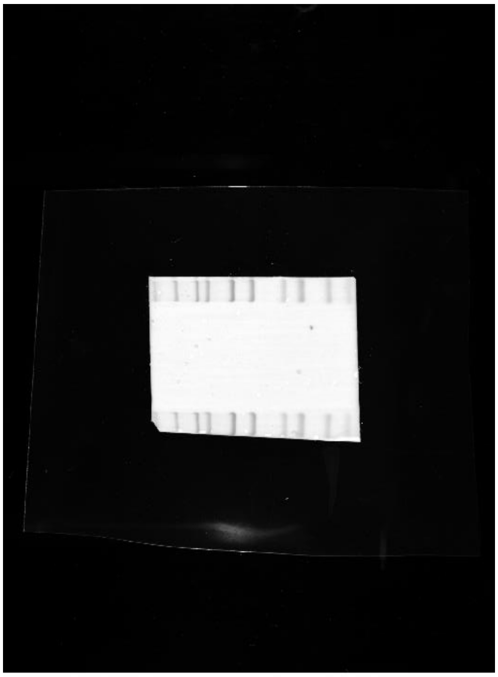

Supplementary data

A whole gel of Figure3c

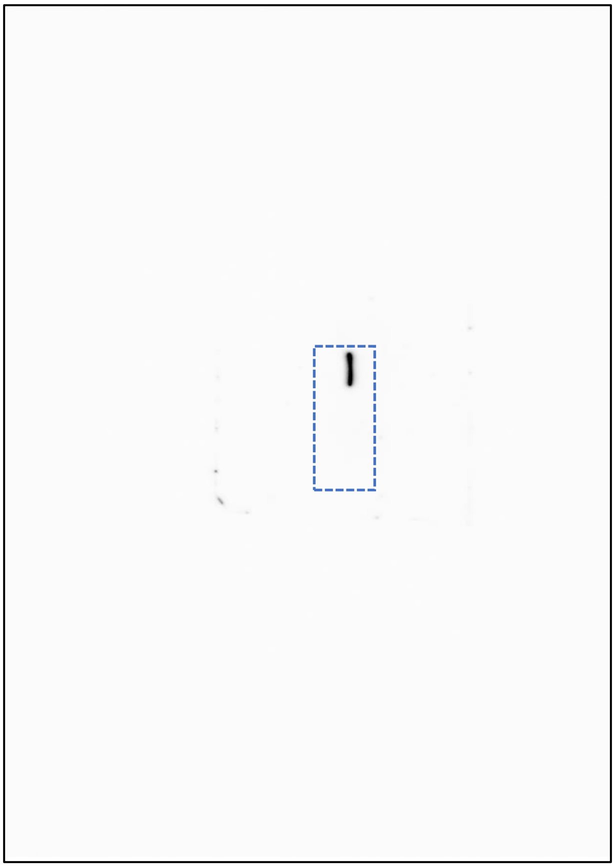

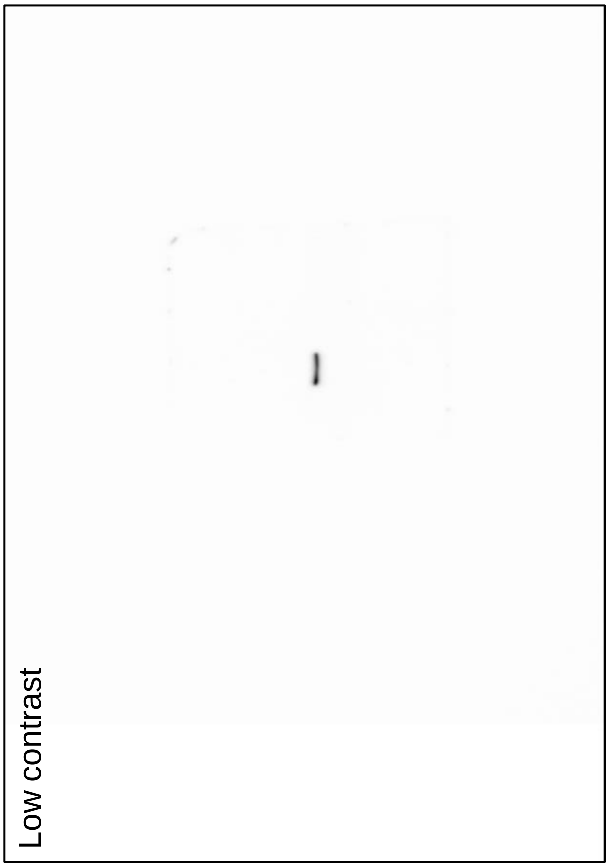

Low contrast

GAPDH

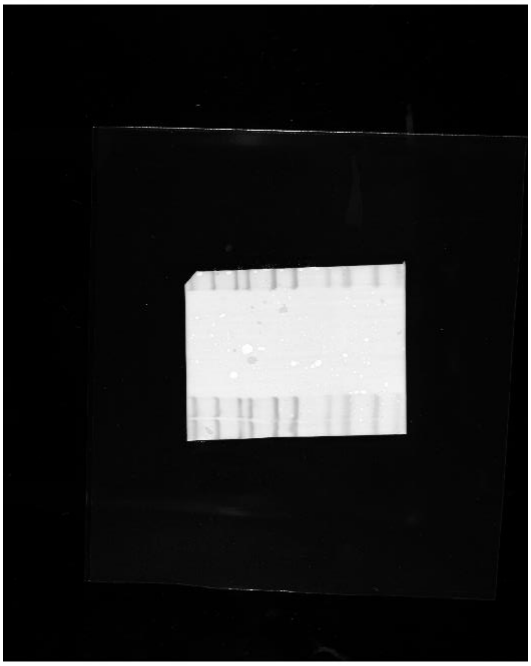

Supplementary data

A whole gel of Figure3d

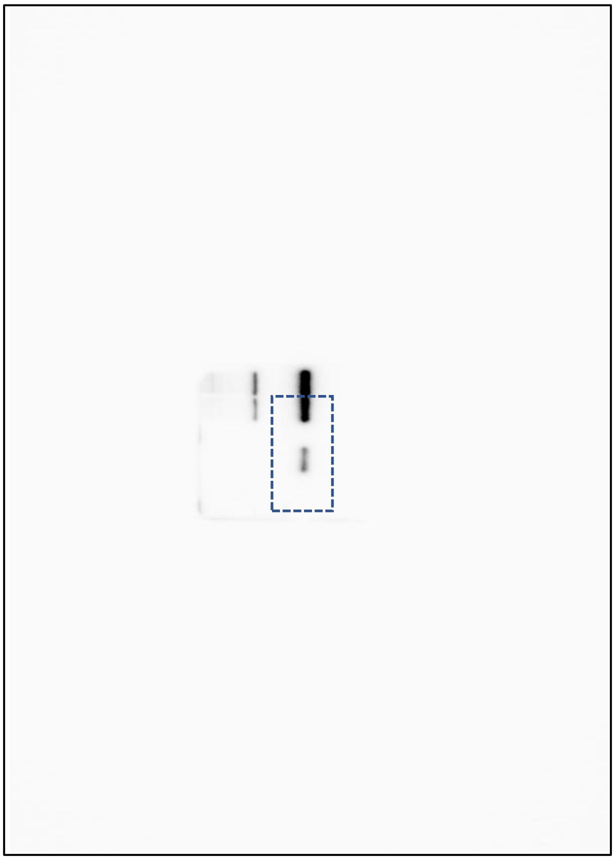

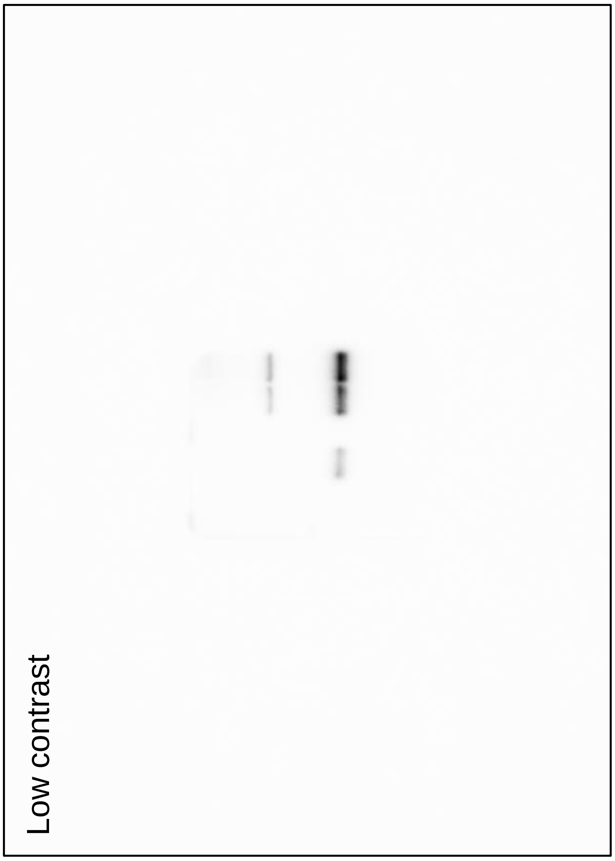

F-mATRAP

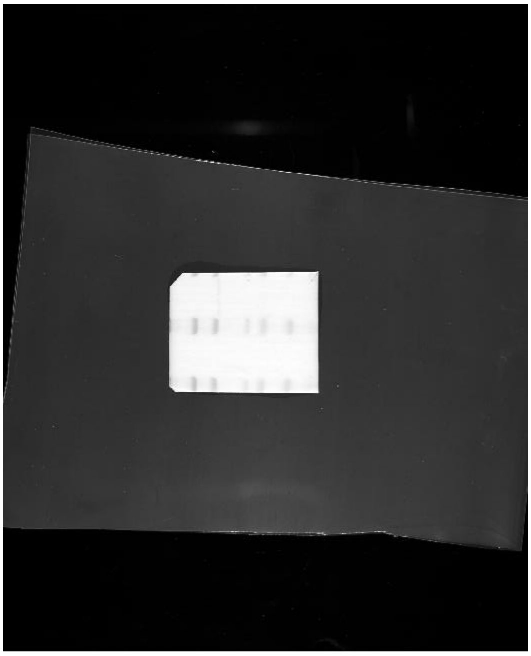

Supplementary data

A whole gel of Figure3d

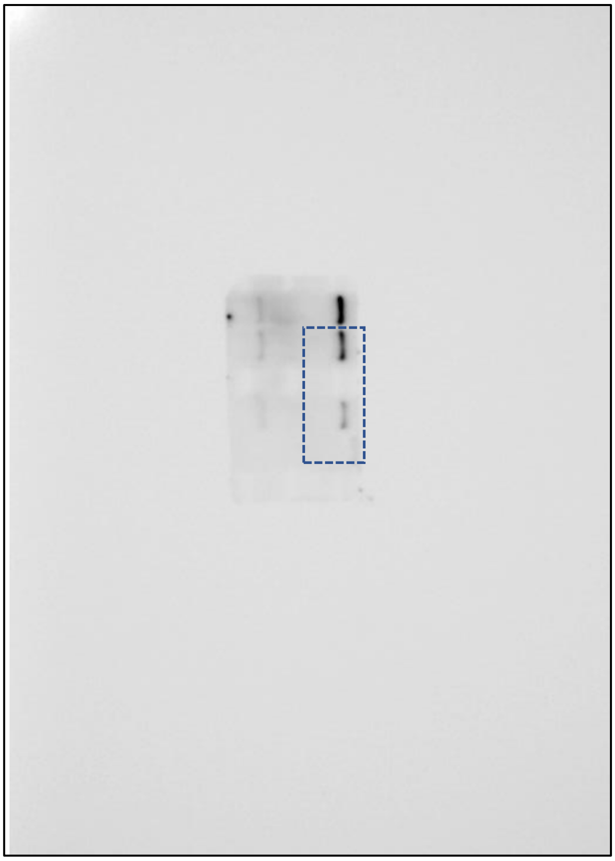

TfR1

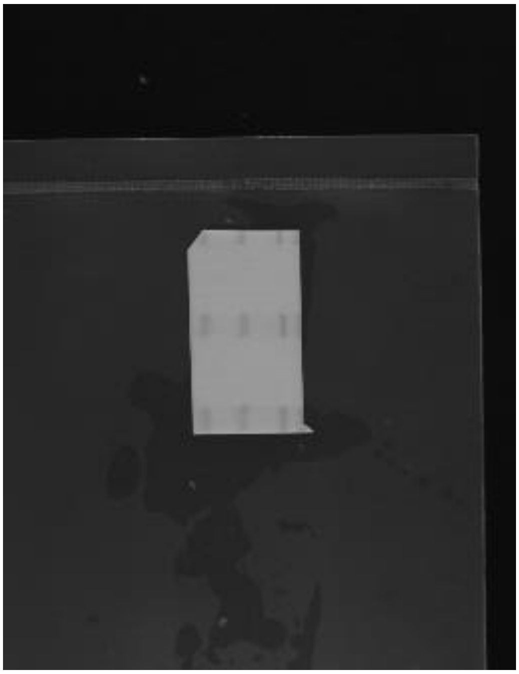

Supplementary data

A whole gel of Figure3d

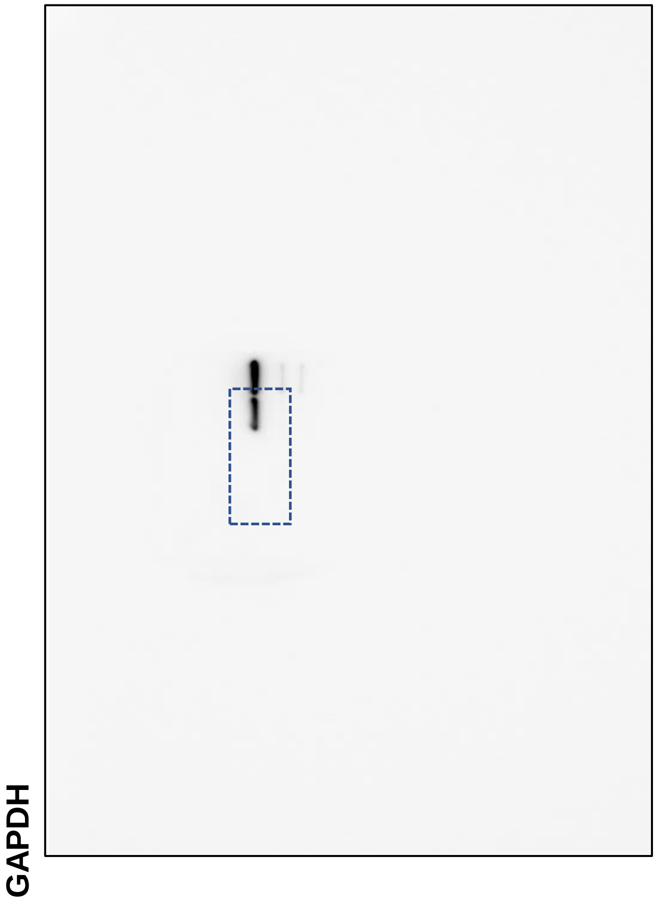

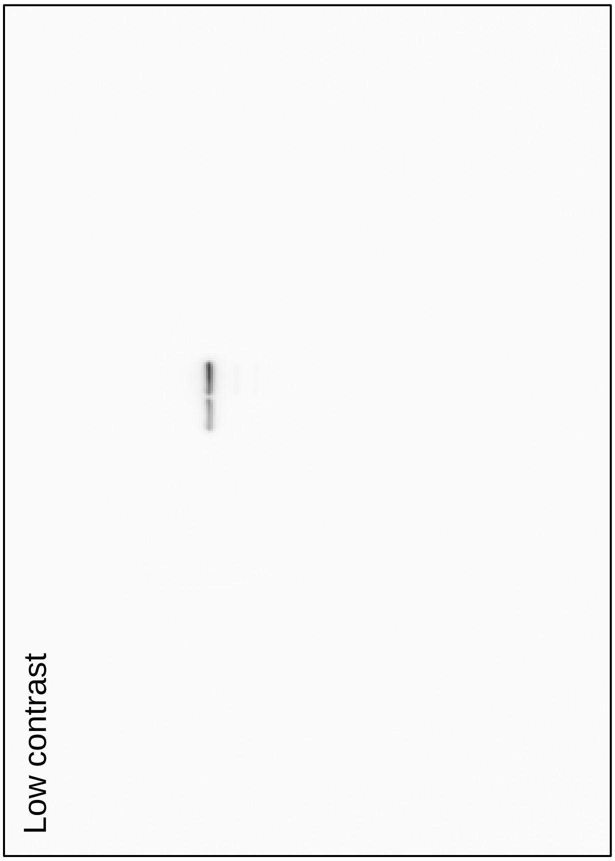

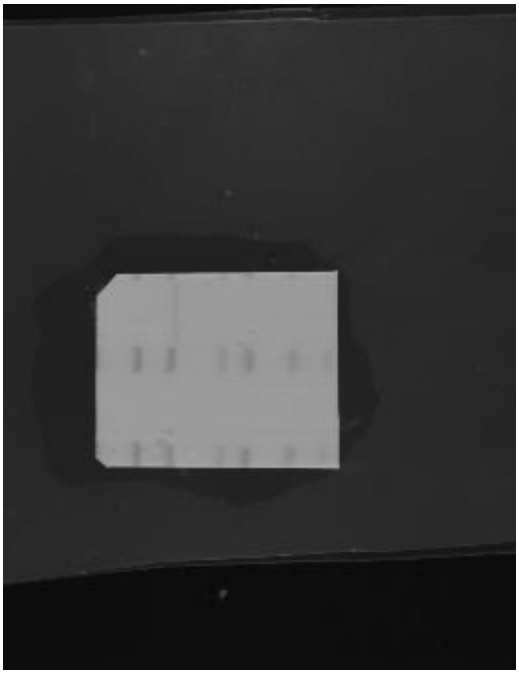

Supplementary data

A whole gel of Figure5a

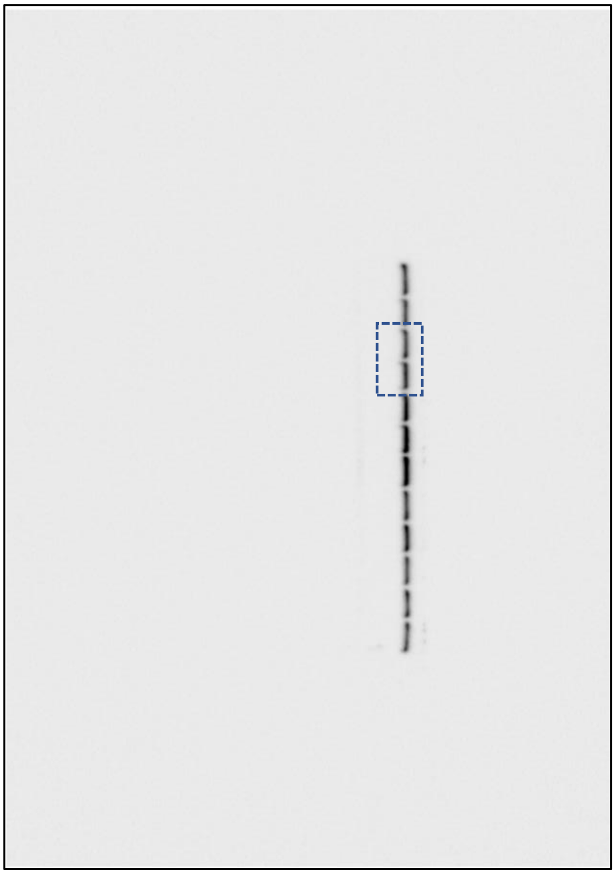

TfR1

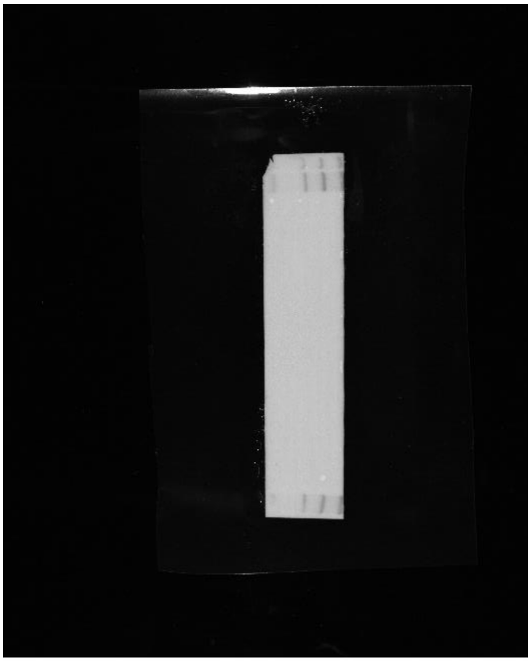

Supplementary data

A whole gel of Figure5a

β-actin

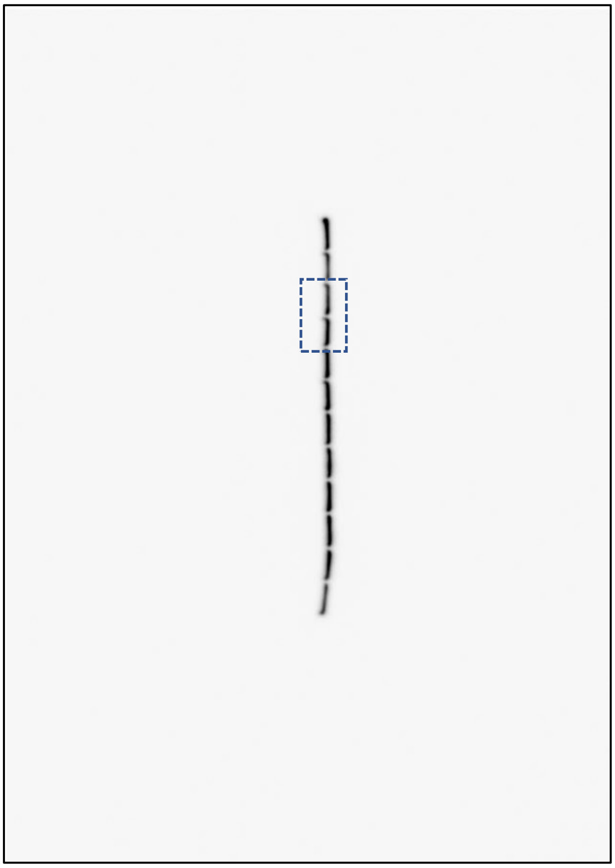

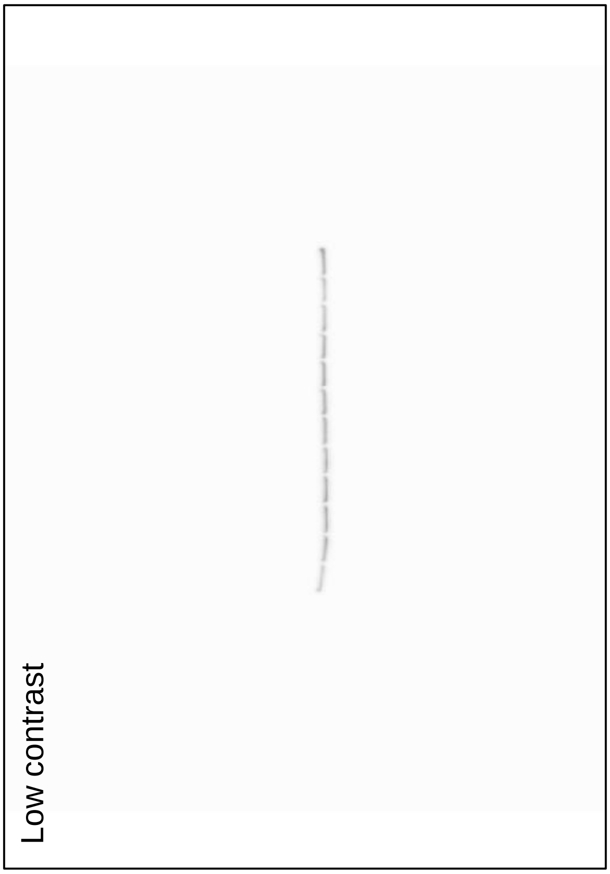

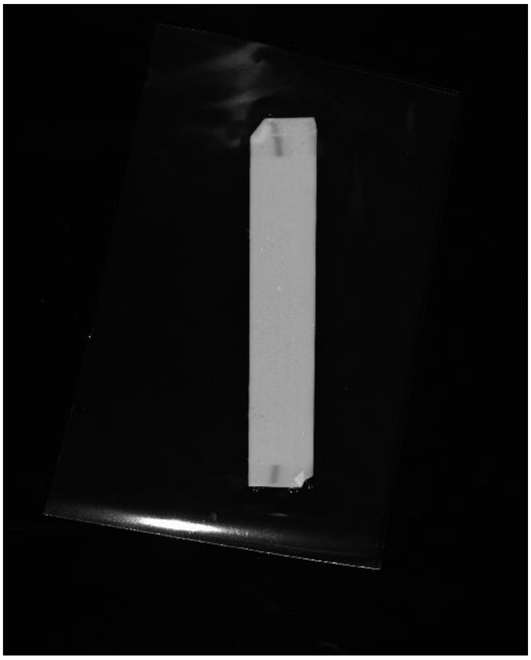

Supplementary data

A whole gel of Figure5a

F-mATRAP

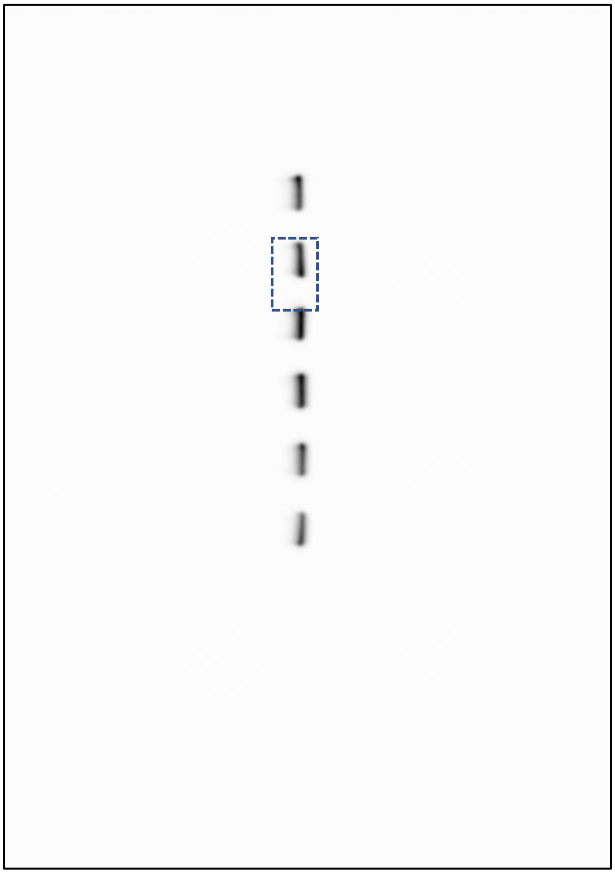

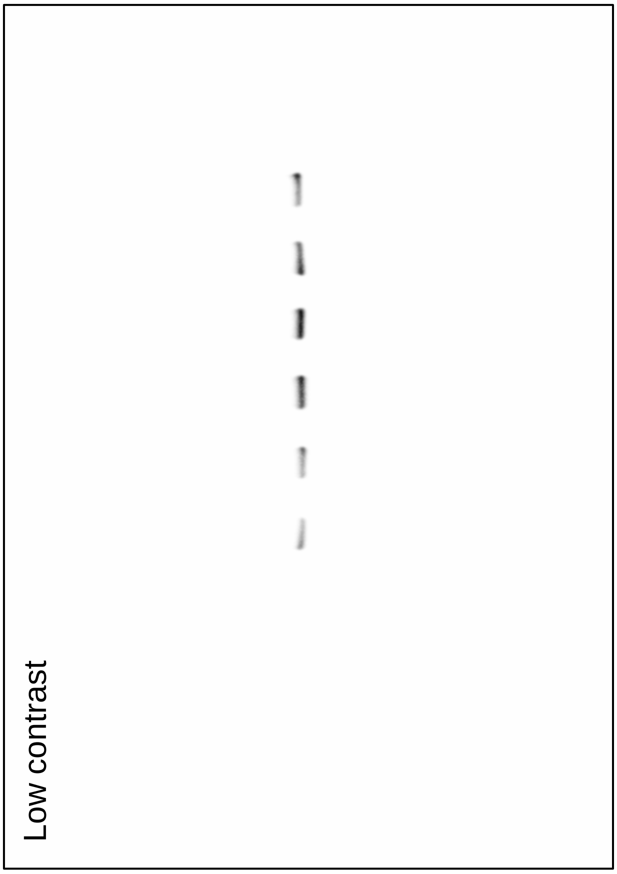

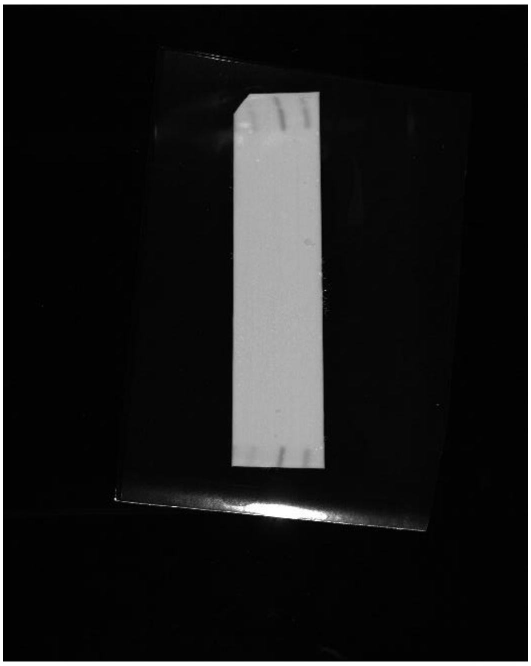

Supplementary data

A whole gel of Figure5c

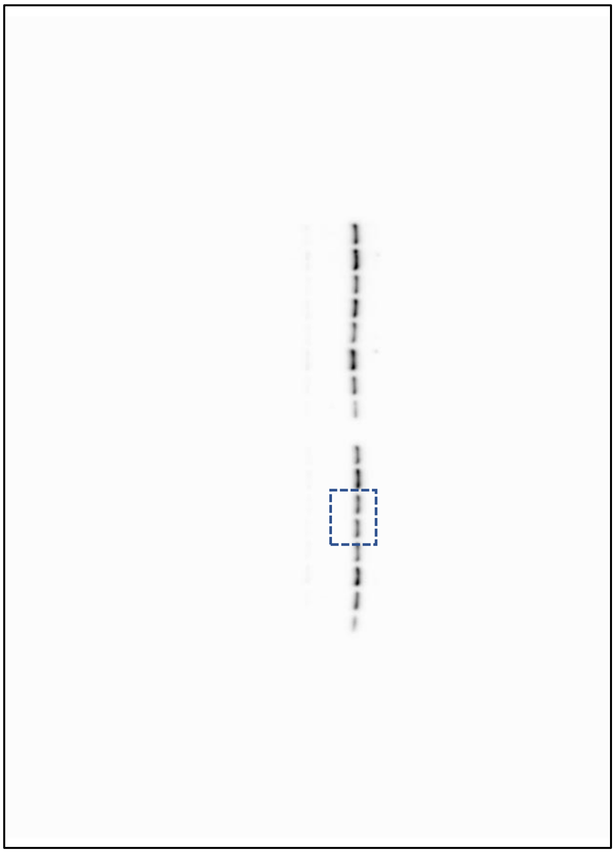

TfR1

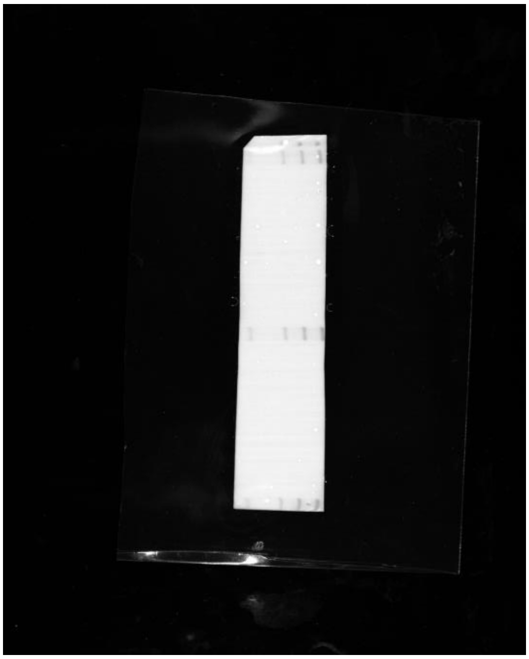

Supplementary data

A whole gel of Figure5c

hATRAP

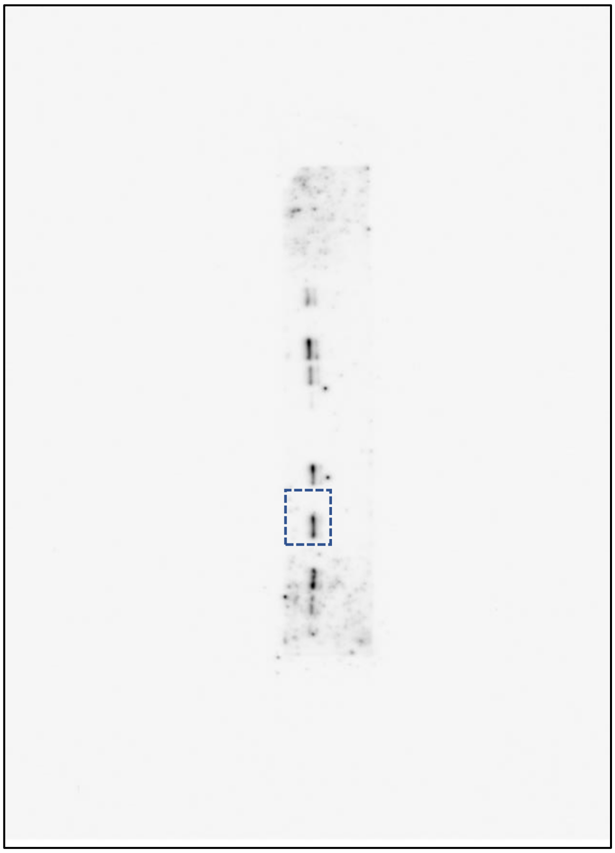

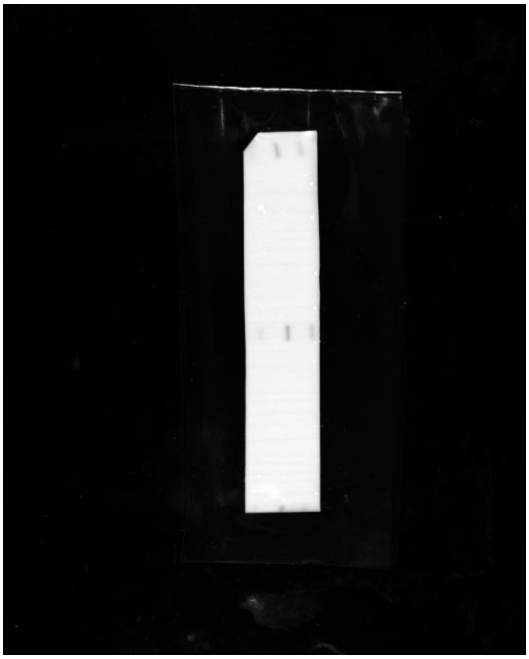

β-actin

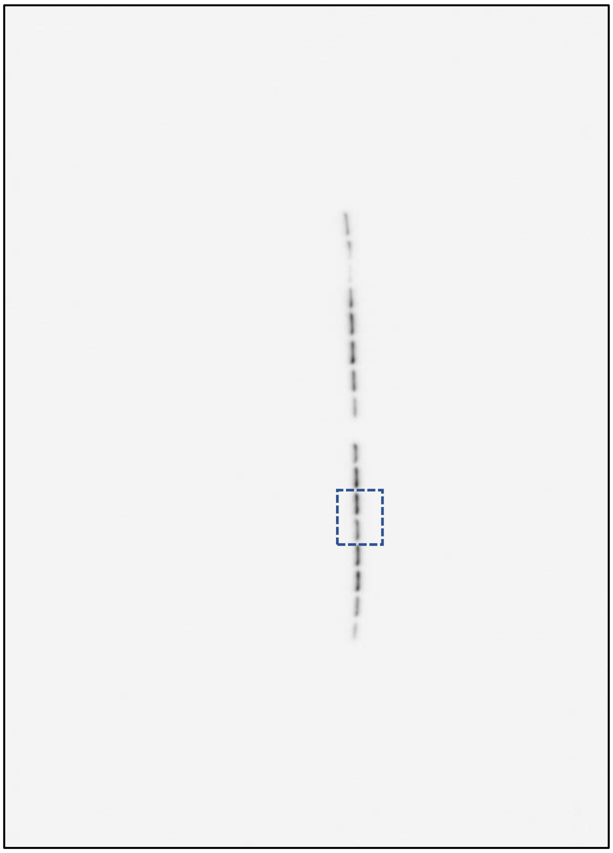

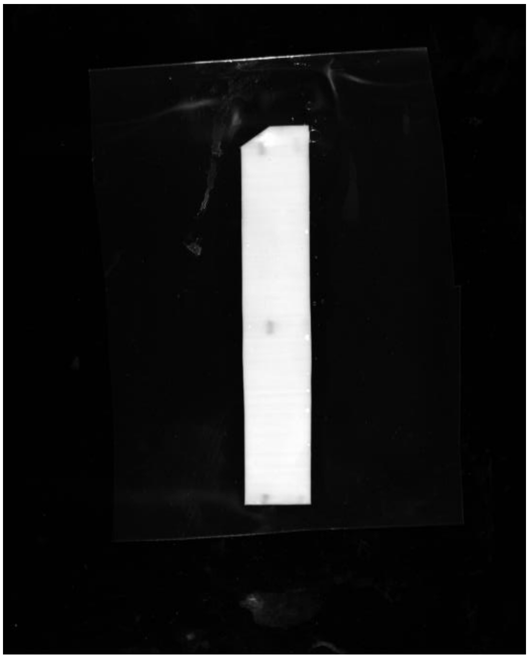

Supplementary data

A whole gel of Figure7a

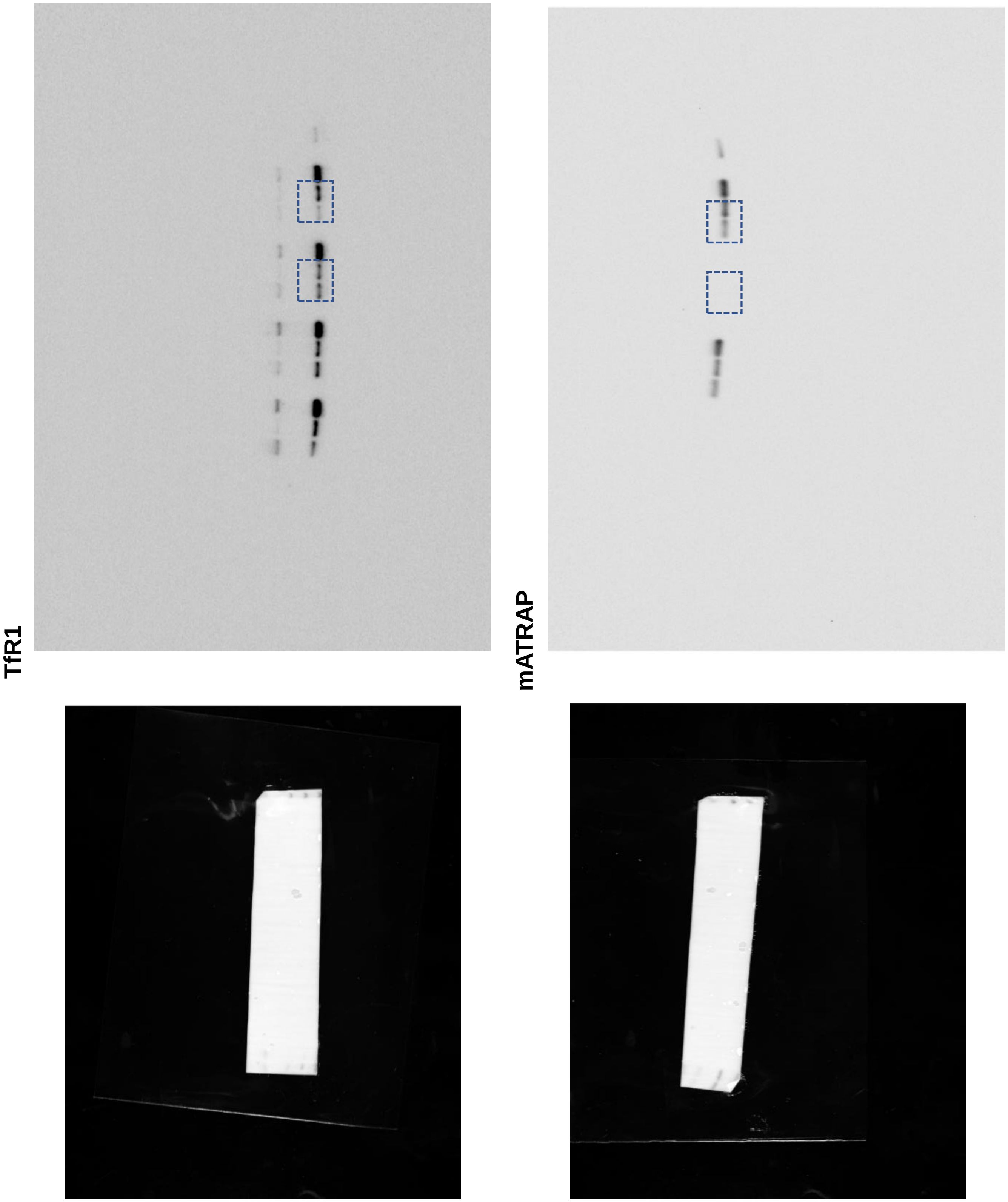

Supplementary data

A whole gel of Figure7b

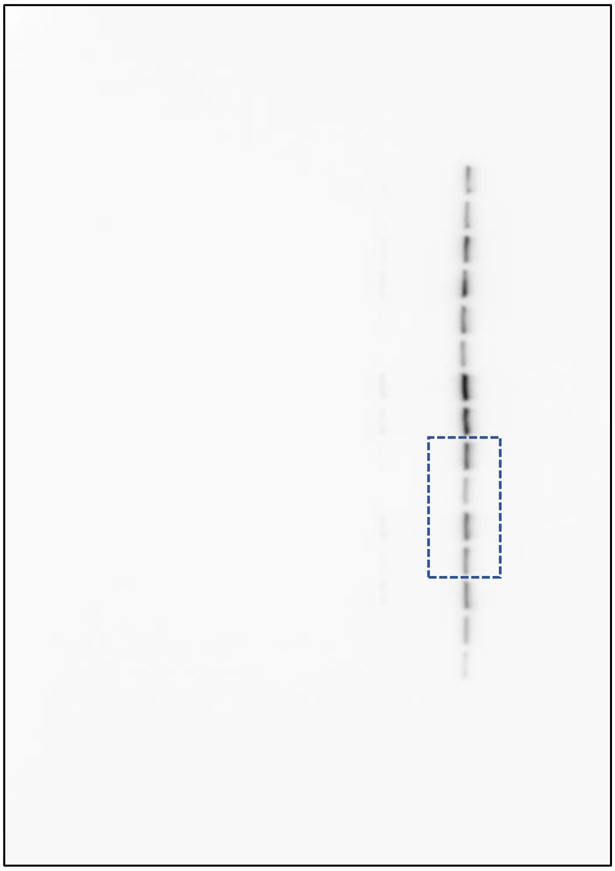

TfR1

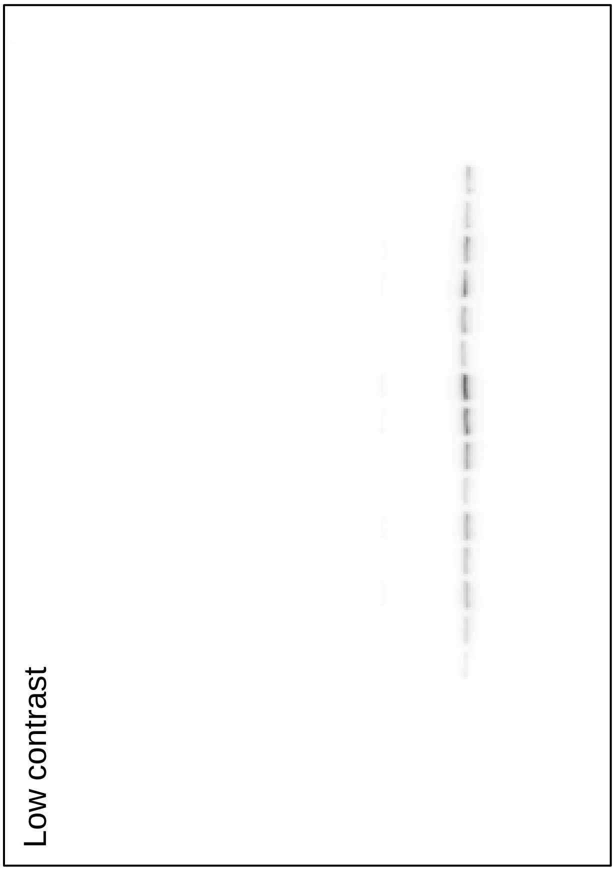

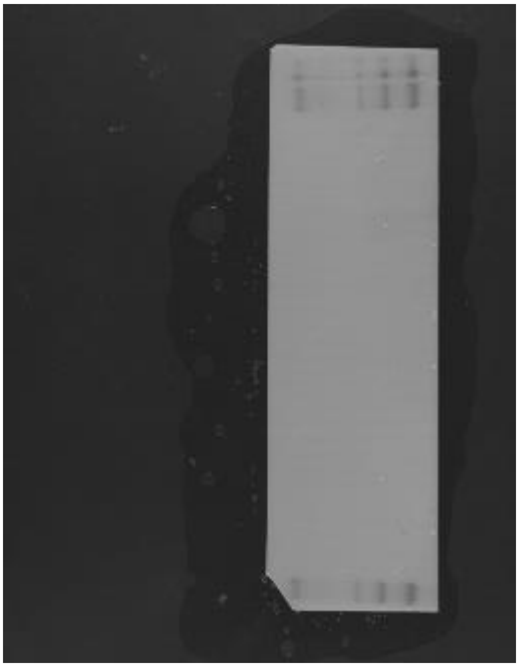

Supplementary data

A whole gel of Figure7b

β-actin

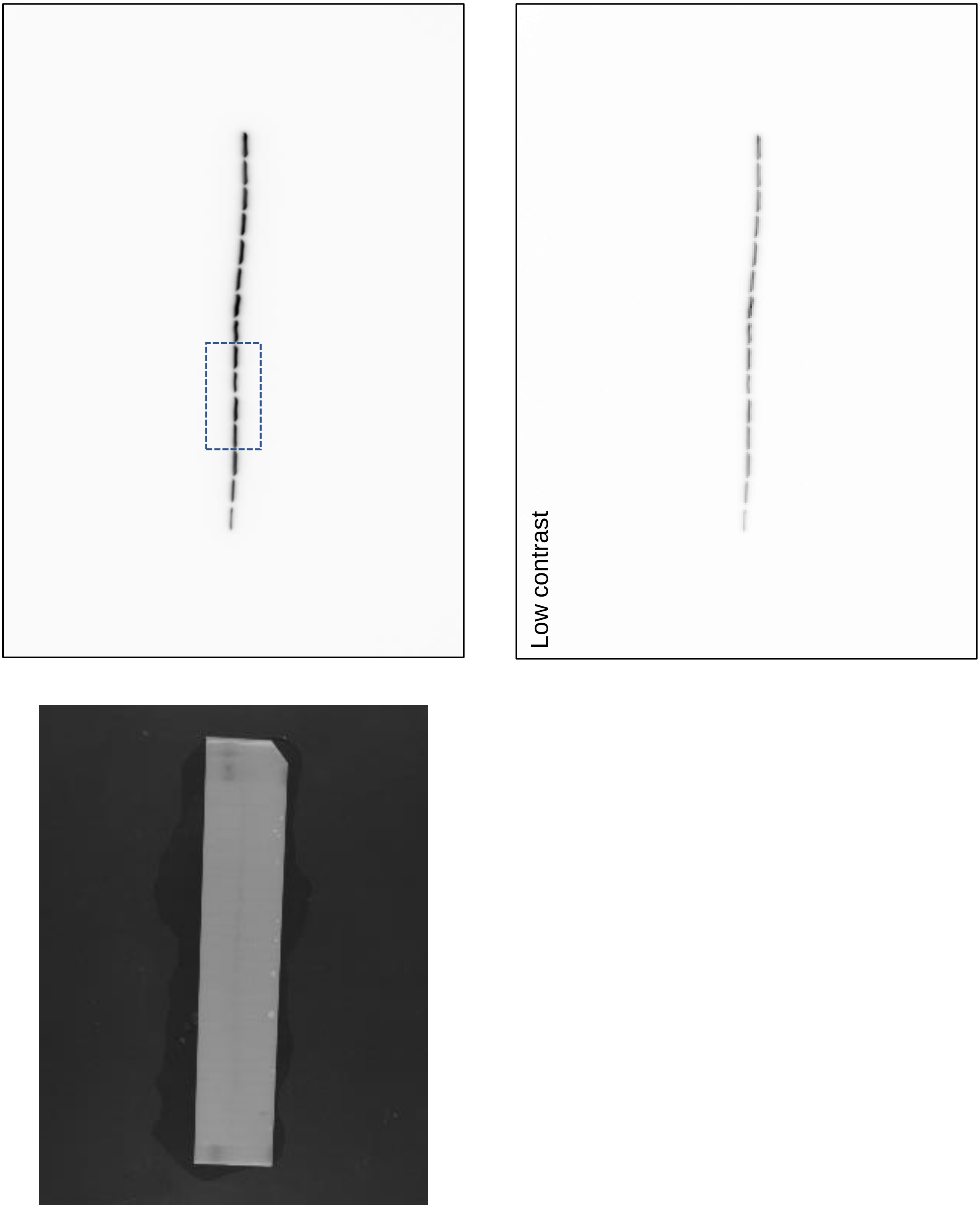

Supplementary data

A whole gel of Figure7b

F-m ATRAP

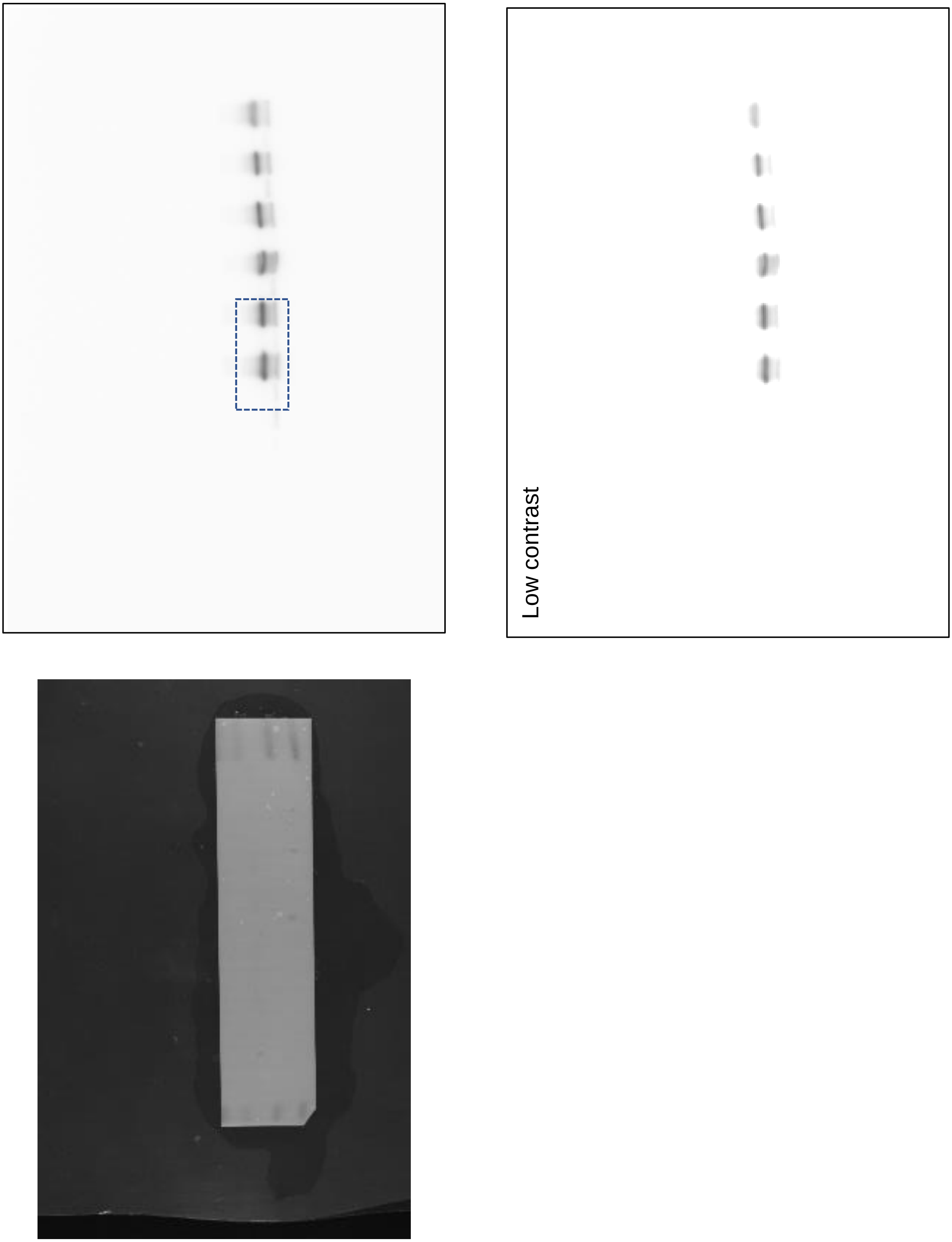

Supplementary data

A whole gel of Figure8b

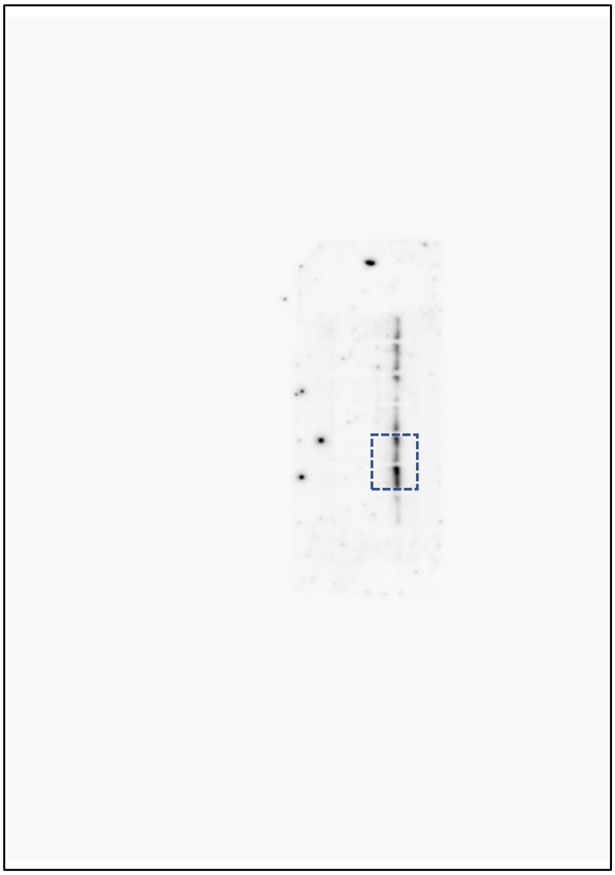

NRF2

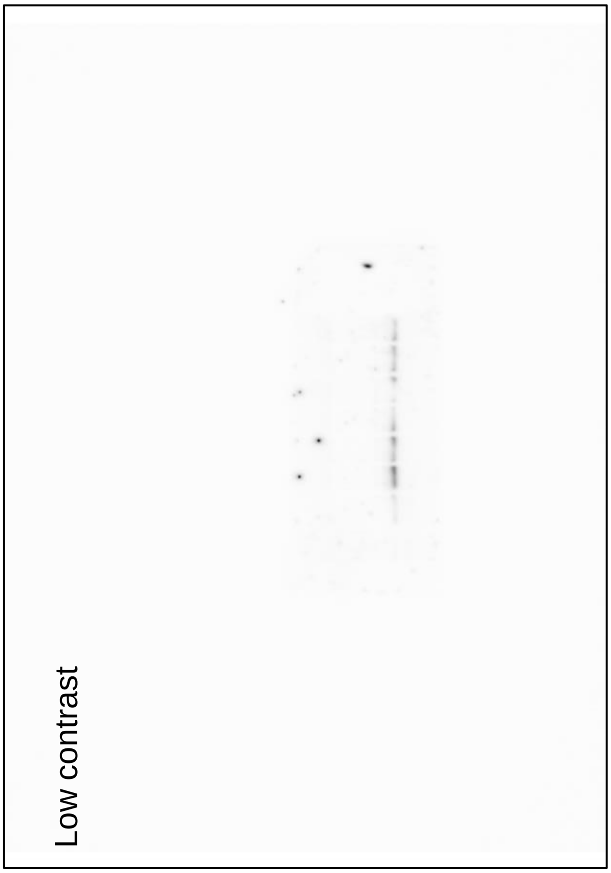

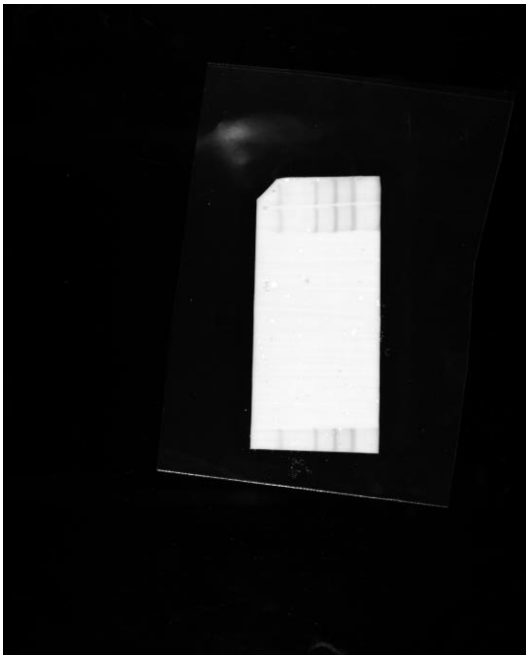

Supplementary data

A whole gel of Figure8b

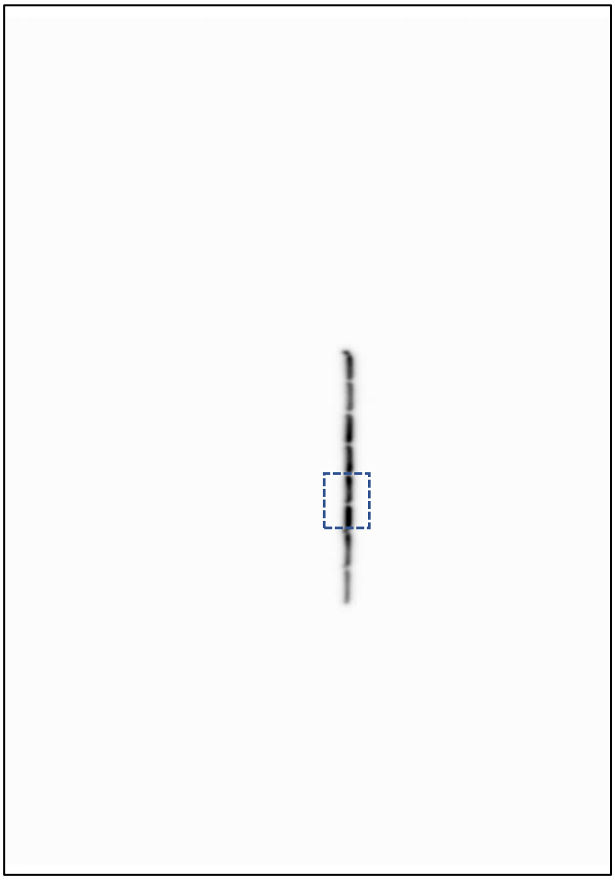

GAPDH

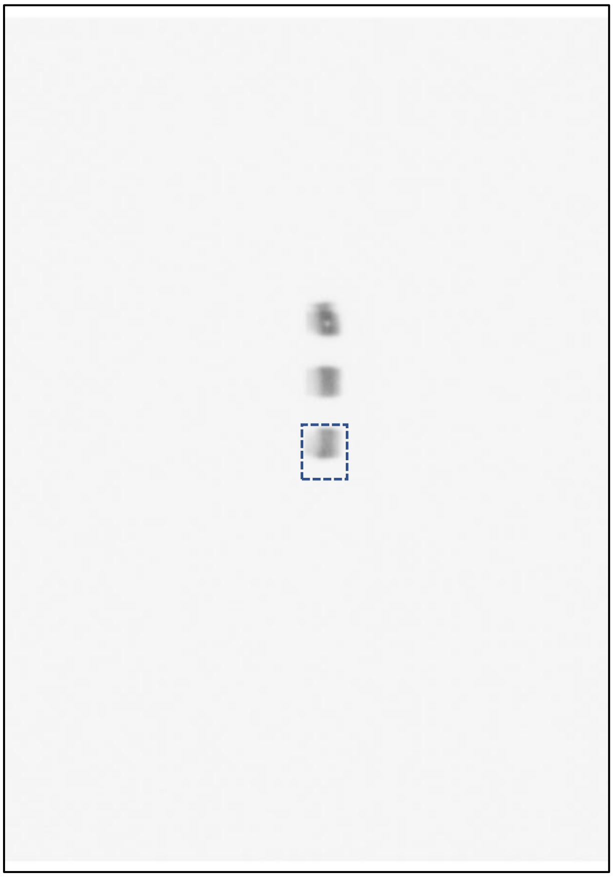

mATRAP

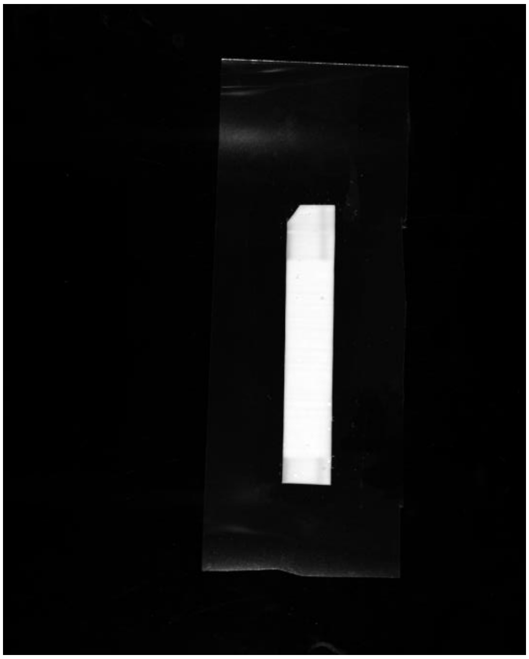

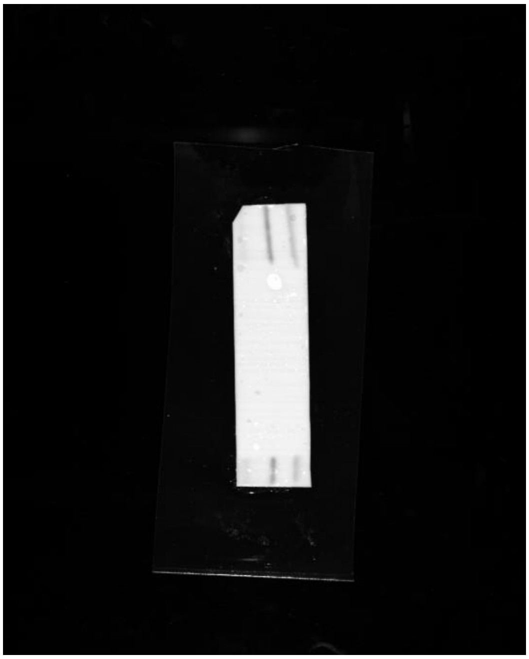

Supplementary data

A whole gel of FigureS1b

endogenous hATRAP and F-mATRAP

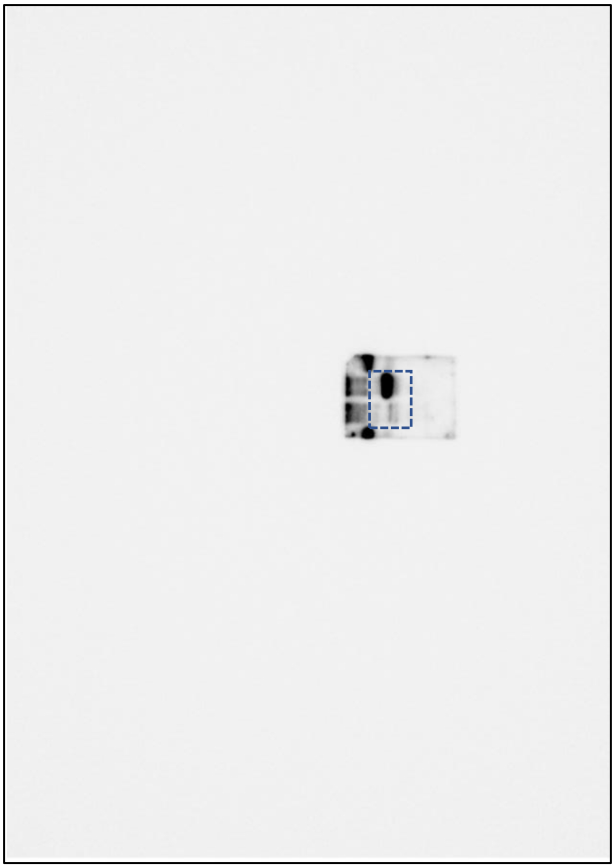

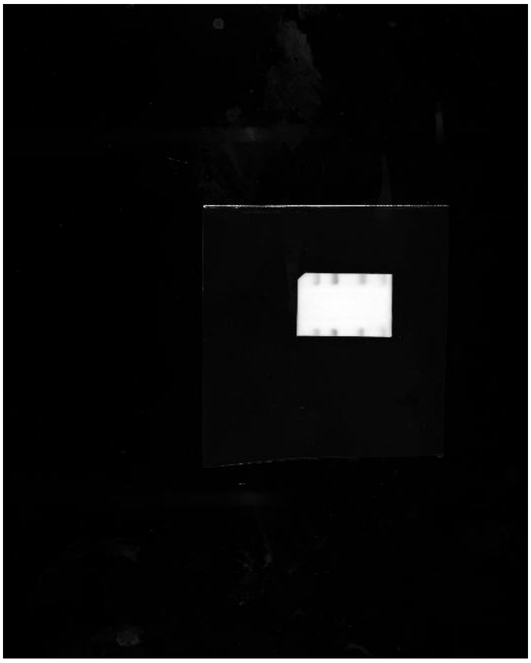

Supplementary data

A whole gel of FigureS1b

GAPDH

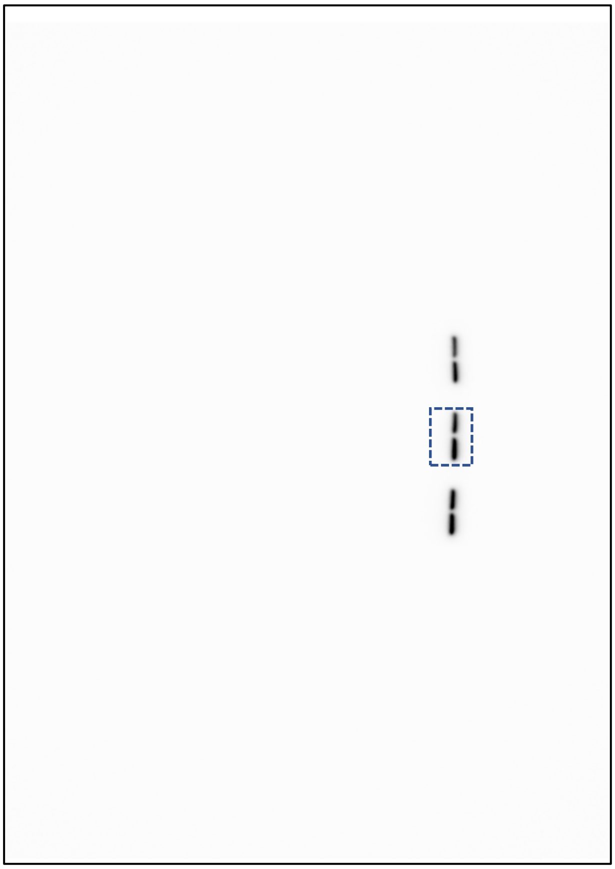

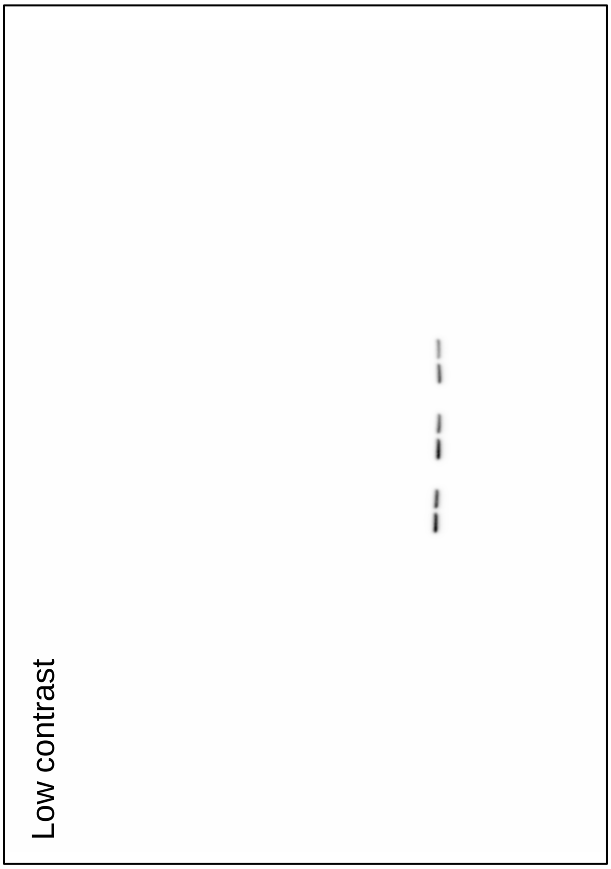

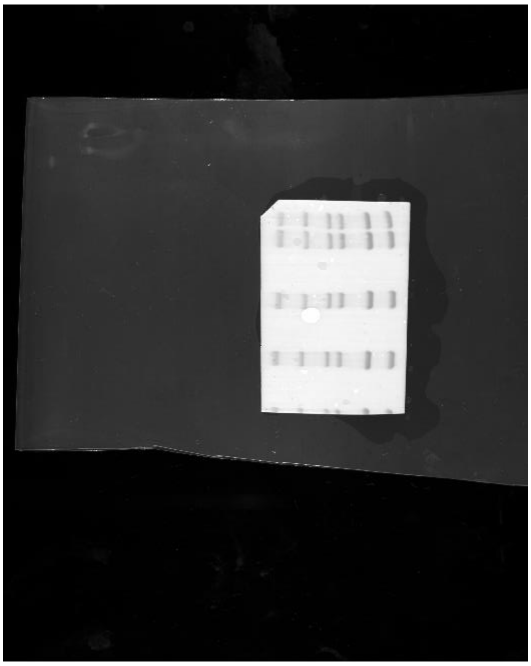

Supplementary data

A whole gel of FigureS1c

endogenous mATRAP

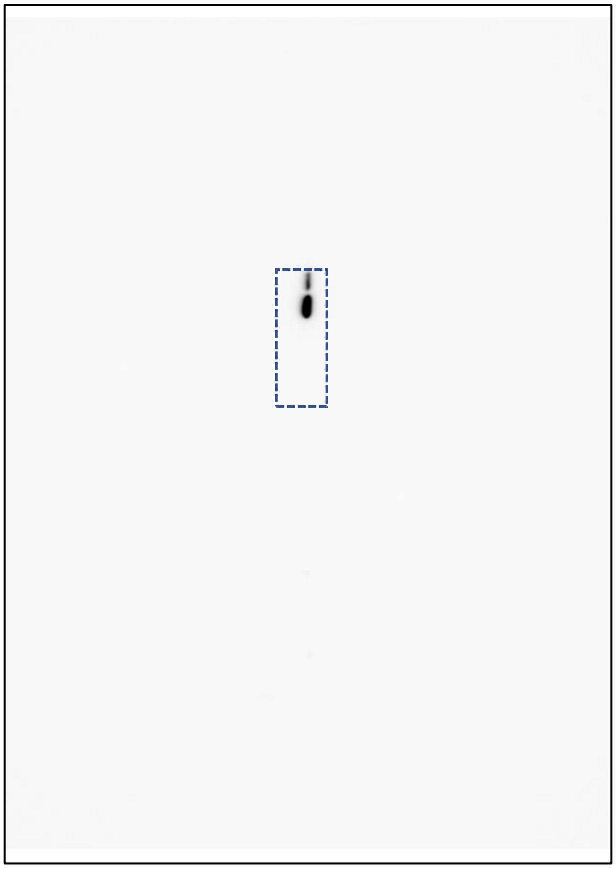

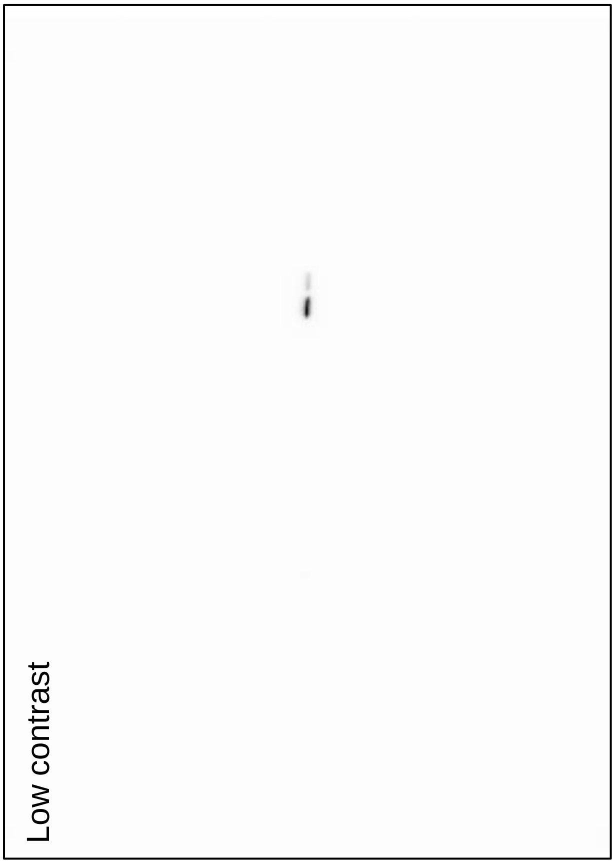

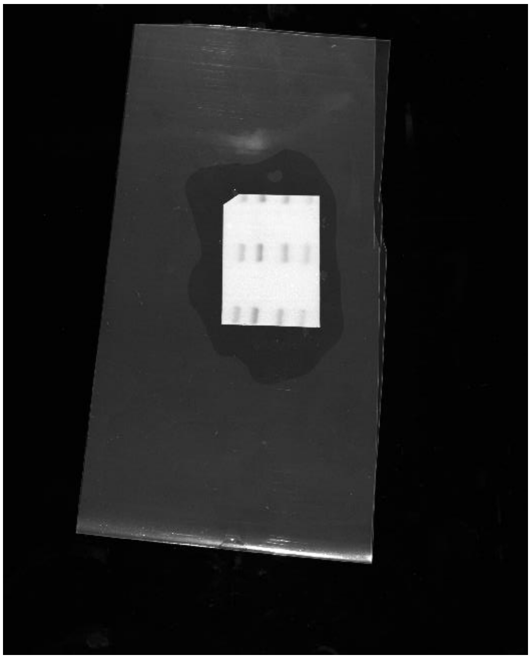

Supplementary data

A whole gel of FigureS1c

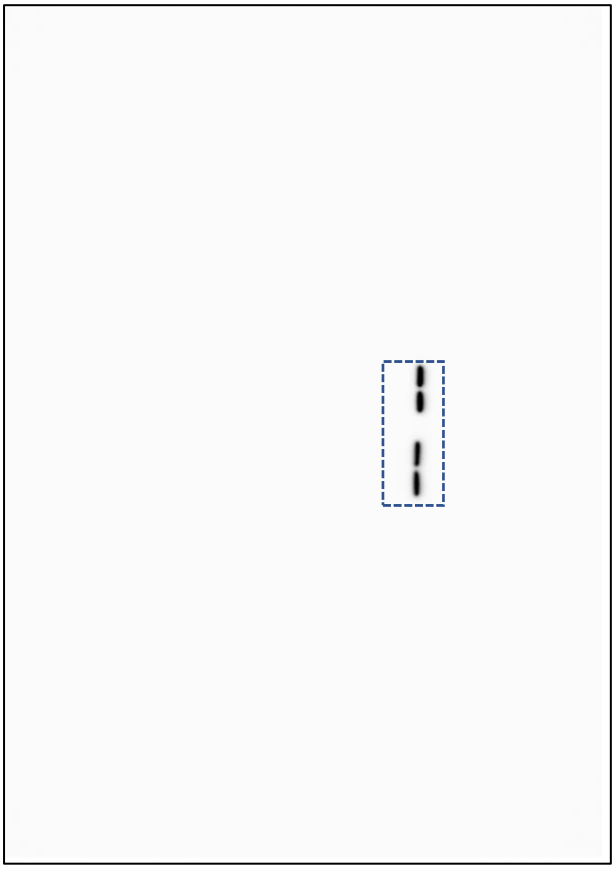

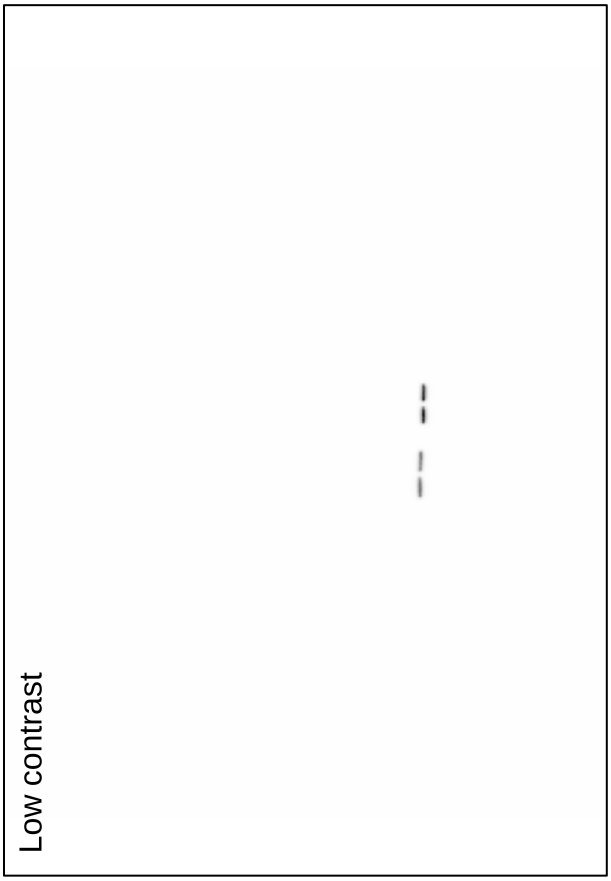

GAPDH

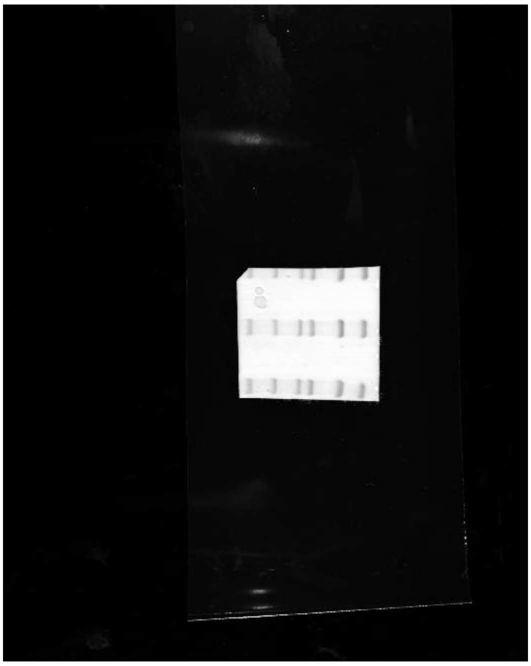

Supplementary data

A whole gel of FigureS1d

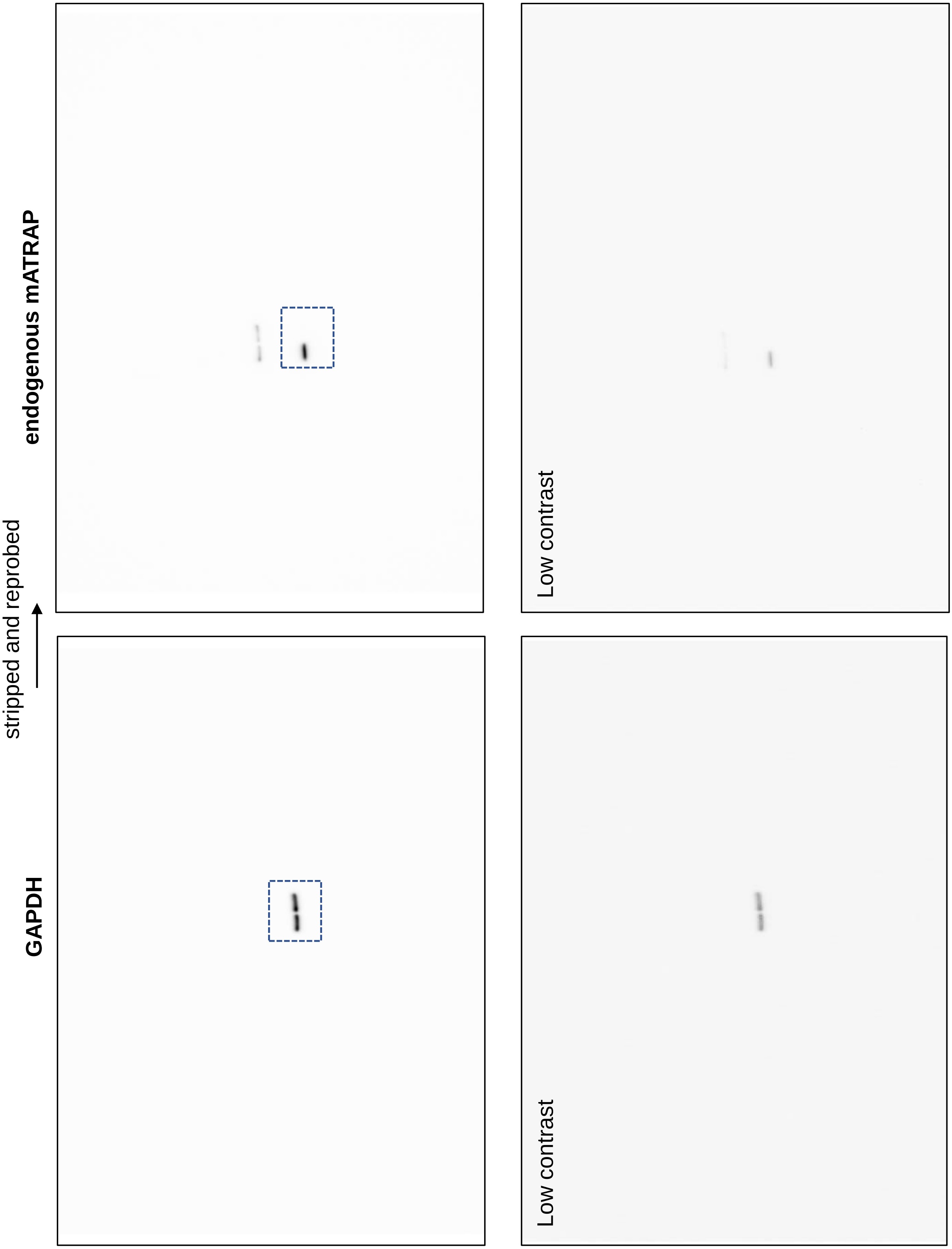

Supplementary data

A whole gel of FigureS1d

endogenous mATRAP
and
GAPDH

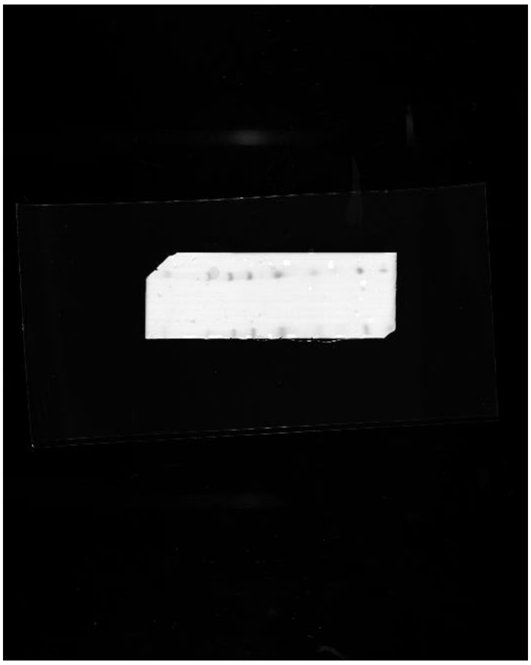

Supplementary data

A whole gel of FigureS2b

TfR1_IP

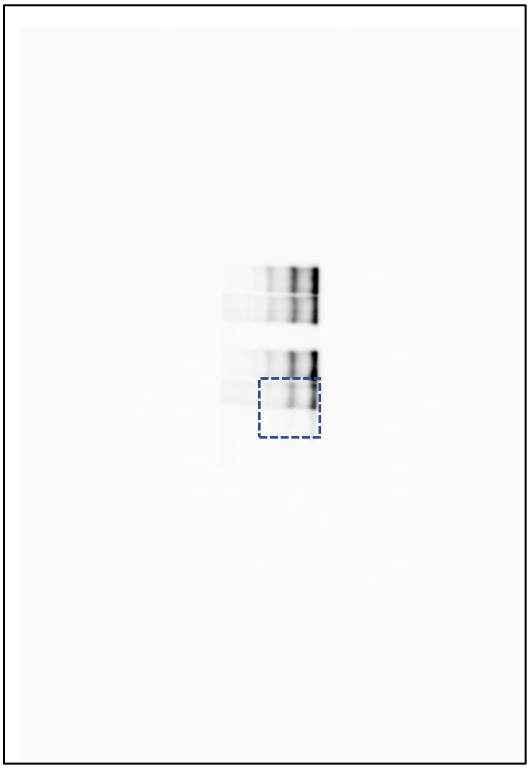

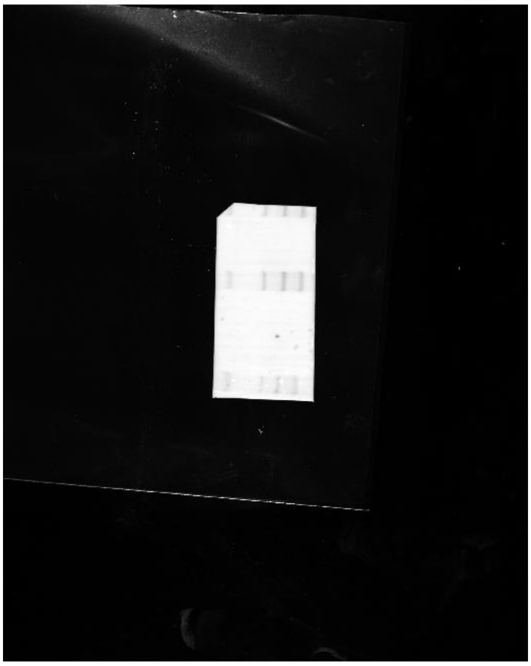

TfR1_Input

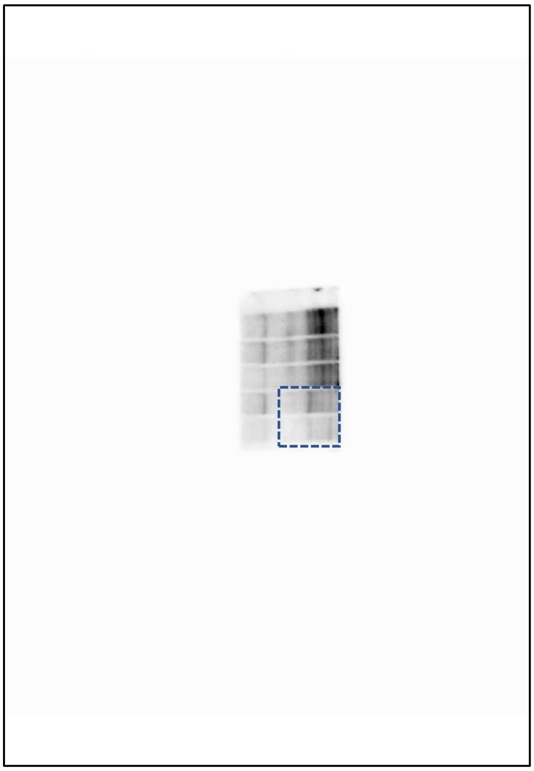

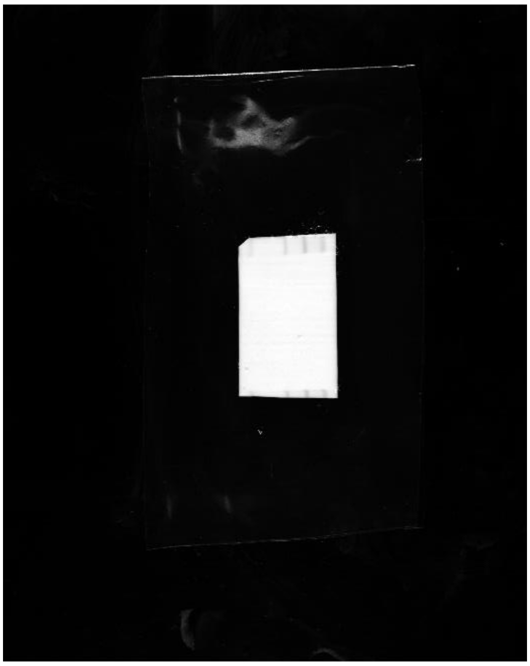

Supplementary data

A whole gel of FigureS2b

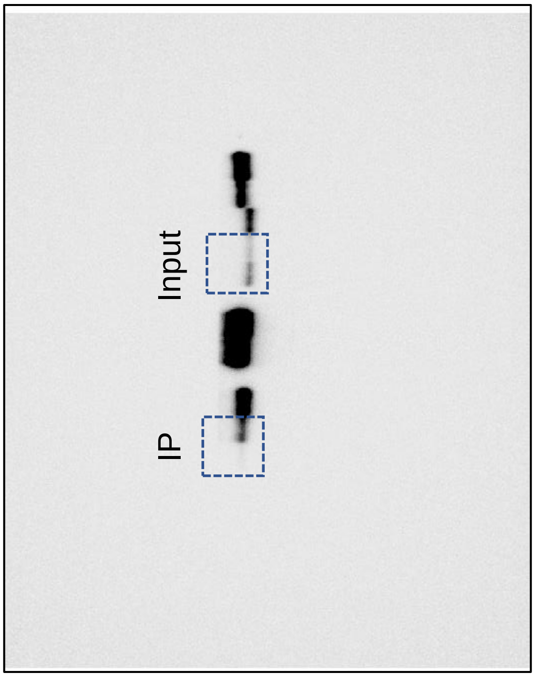

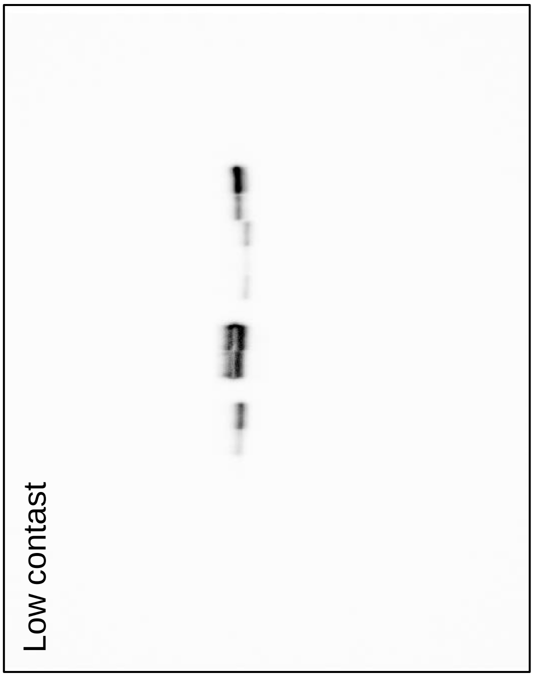

mATRAP

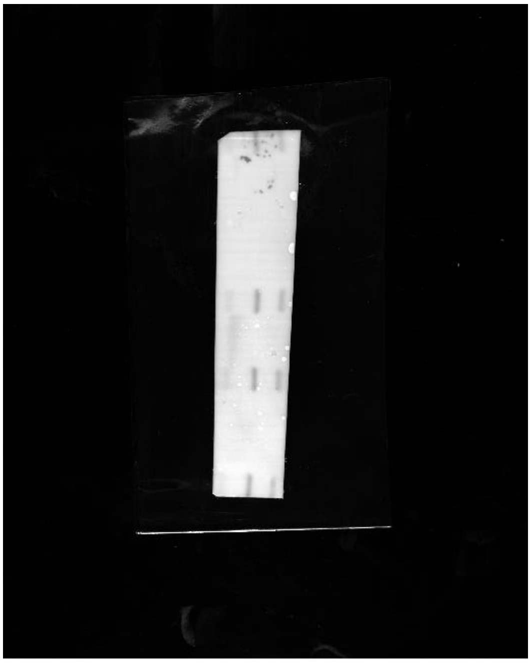

Supplementary data

A whole gel of FigureS2b

GAPDH

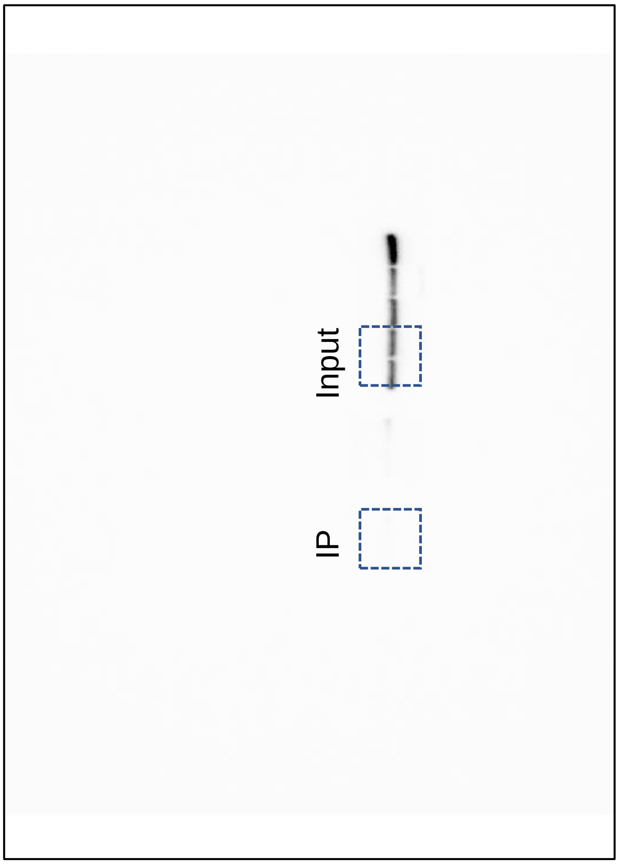

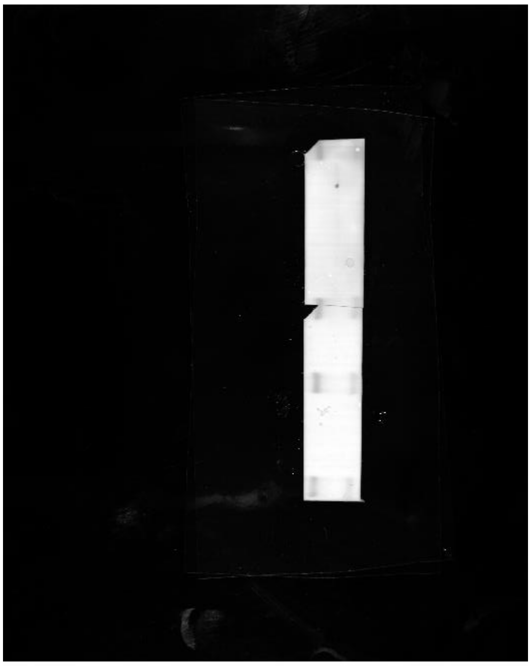

Supplementary data

A whole gel of FigureS5

TfR1

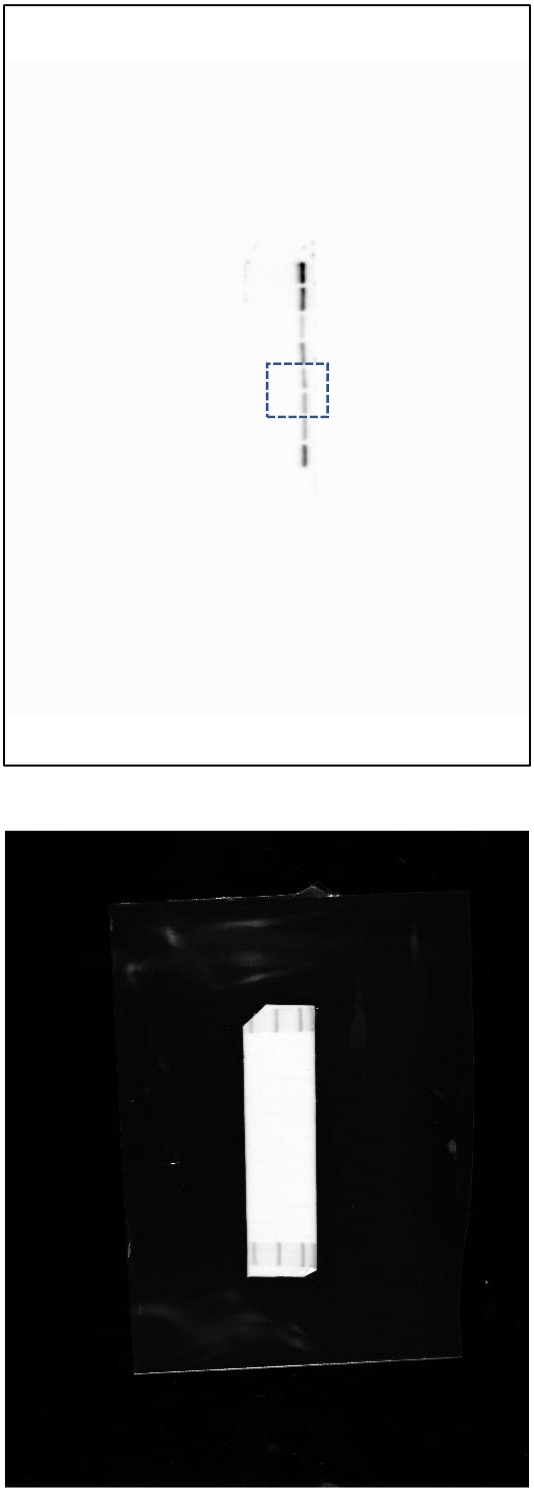

mATRAP

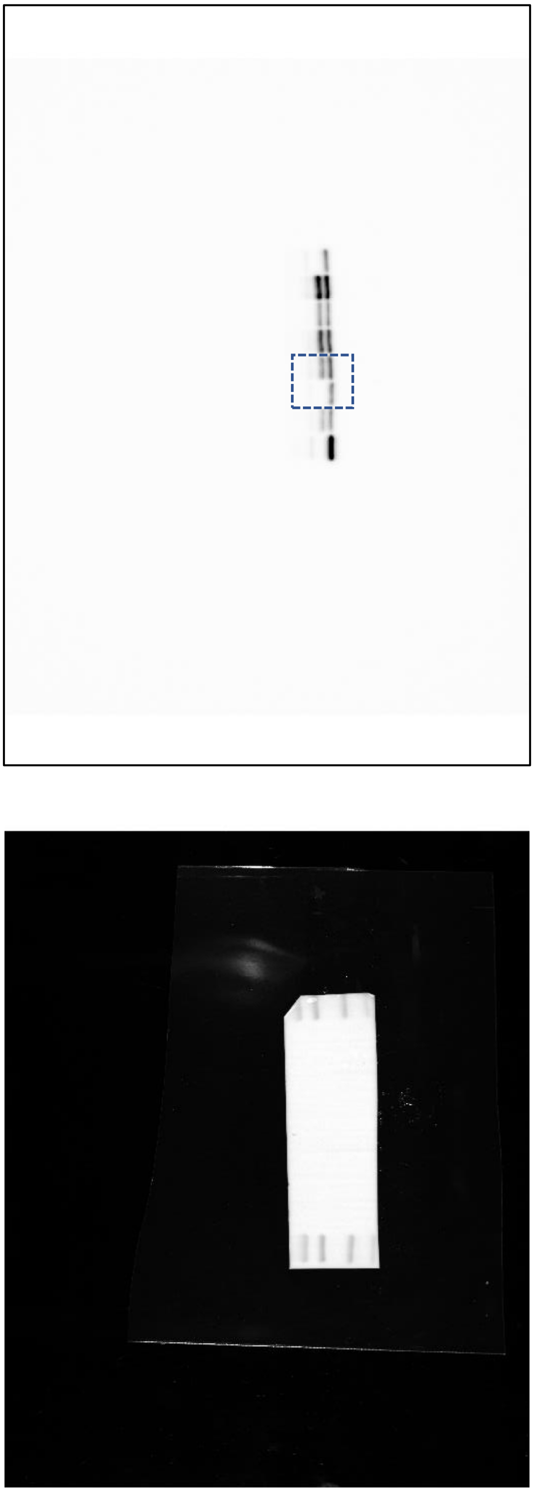

Supplementary data

A whole gel of FigureS5

GAPDH

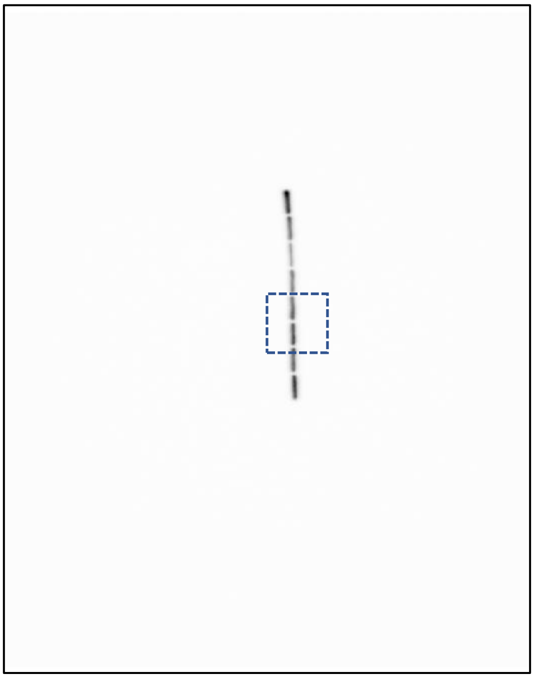

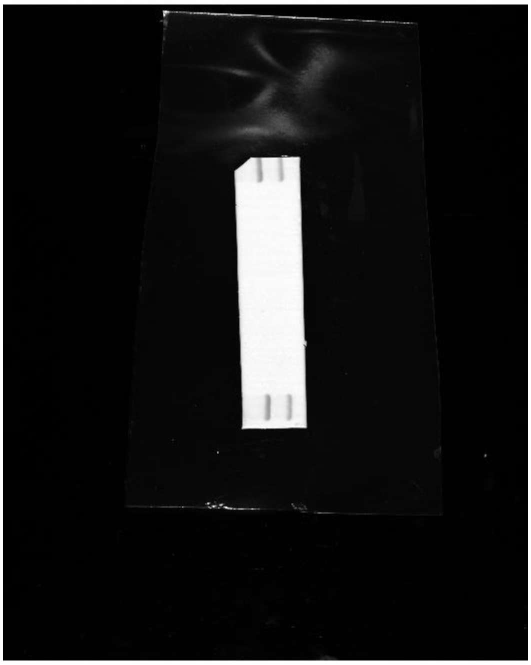

Supplementary data

A whole gel of FigureS6

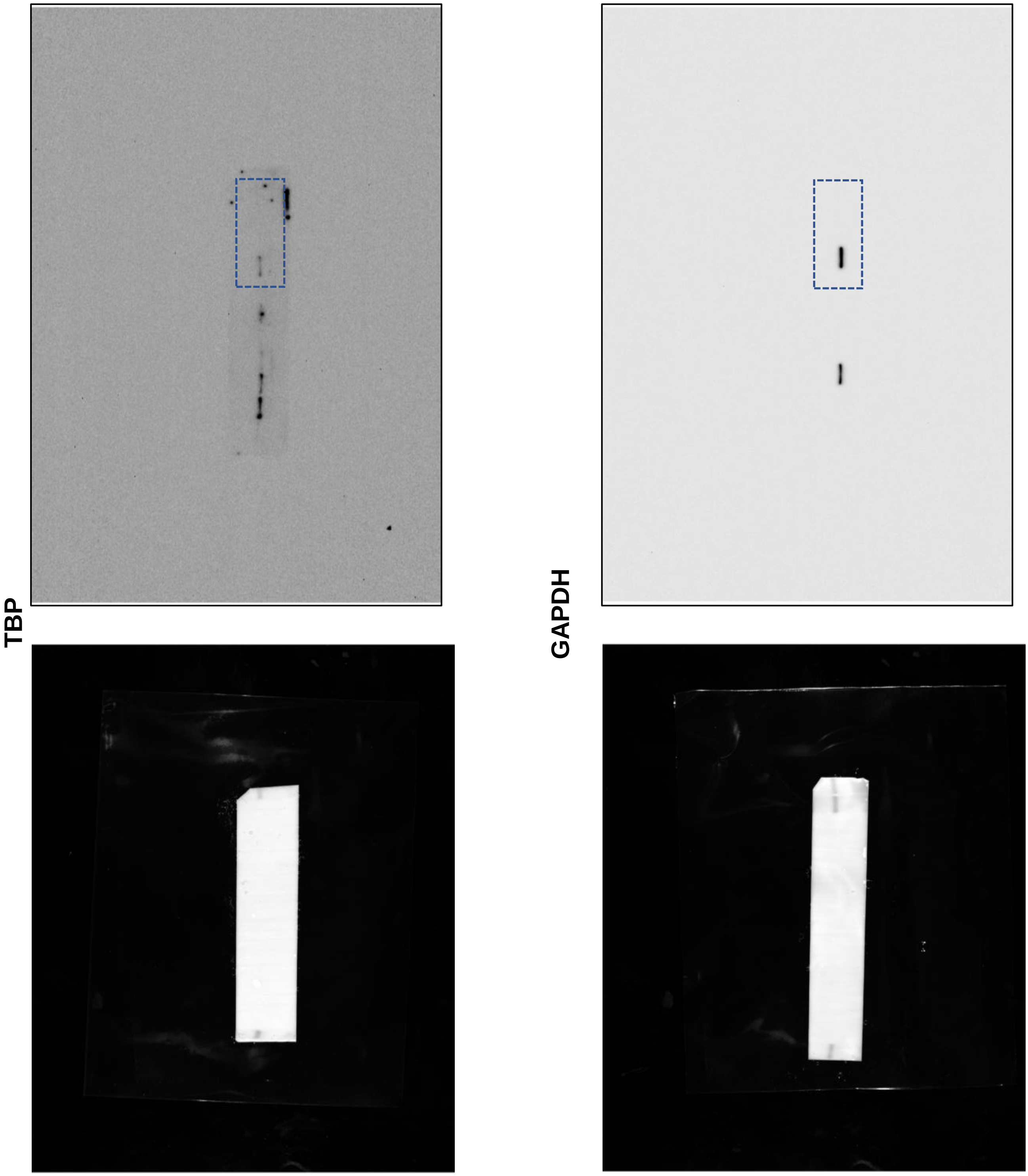

Supplementary data

A whole gel of FigureS6

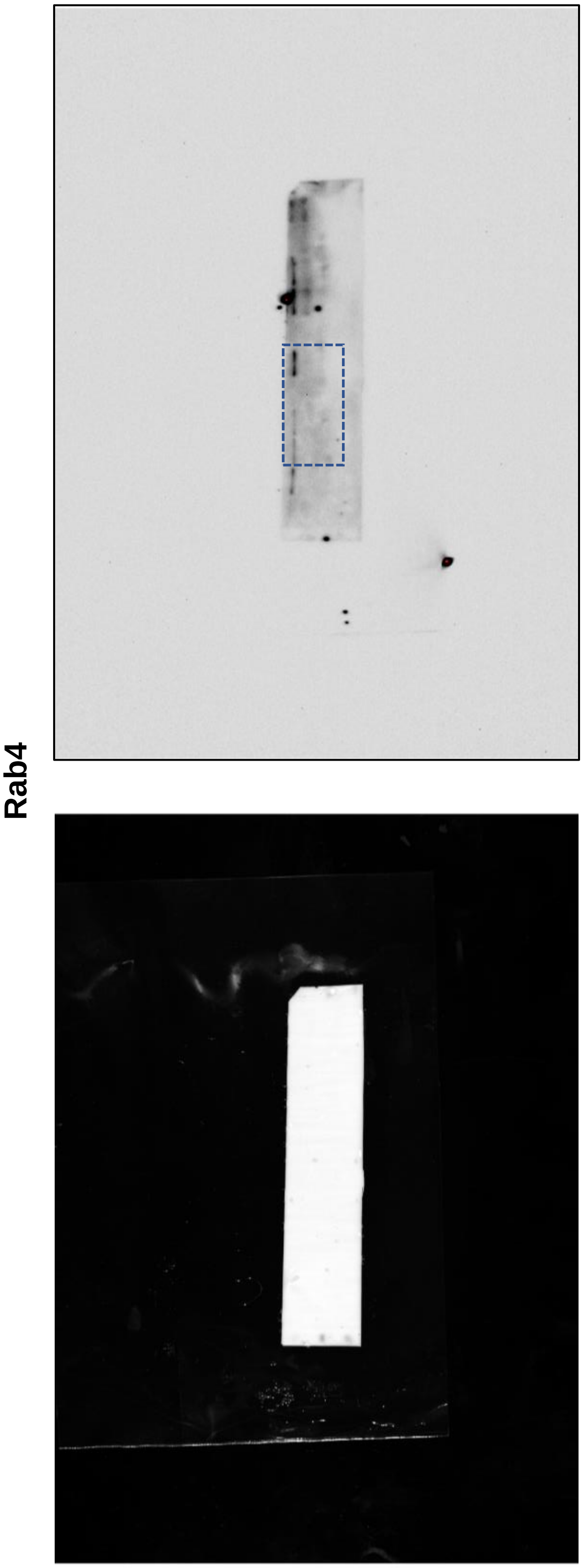

Supplement: Supplementary file 2 — Supplementary Information. [file 41598_2022_22343_MOESM2_ESM.pdf]
